# Integrated analysis of the aqueous humor microbiome and lens capsule transcriptome in high myopia cataract: a pilot study

**DOI:** 10.3389/fmed.2026.1845205

**Published:** 2026-06-16

**Authors:** Yaoyan Qiu, Wenting Cai, Zihan Xie, Chenxi Li, Fan Yang, Yiyong Qian, Jingwu Song, Tianyu Zheng

**Affiliations:** 1 Department of Ophthalmology, Shanghai Tenth People’s Hospital, Tongji University School of Medicine, Shanghai, China; 2 Visual Rehabilitation Center, Tongji University School of Medicine, Shanghai, China; 3 Tongji Eye Institute, Tongji University School of Medicine, Shanghai, China; 4 Department of Ophthalmology, Eye and ENT Hospital, Fudan University, Shanghai, China; 5 Eye Institute, Eye and ENT Hospital, Fudan University, Shanghai, China; 6 NHC Key Laboratory of Myopia and Related Eye Diseases, Shanghai, China; 7 Shanghai Key Laboratory of Visual Impairment and Restoration, Shanghai, China; 8 Department of Functional Intestinal Diseases, General Surgery of Shanghai Tenth People’s Hospital, Tongji University School of Medicine, Shanghai, China; 9 Shen 1001 Life Institute, Shanghai, China

**Keywords:** apoptosis, aqueous humor microbiome, cataract, high myopia, lens capsule, multi-omics integration, transcriptome

## Abstract

**Introduction:**

High myopia is a critical risk factor that accelerates cataract onset and increases surgical complexity, yet the underlying mechanisms remain incompletely understood.

**Methods:**

In this study, we collected aqueous humor samples (6 from high myopia cataract patients and 6 from age-related cataract controls) and lens capsule samples (3 per group). 2bRAD-M gene sequencing was used to characterize the aqueous humor microbiome, while RNA-seq was performed to profile transcriptomic changes in the lens capsule.

**Results:**

Patients with high myopia cataract exhibited significant alterations in aqueous humor microbial diversity, with decreased abundance of *Escherichia* and increased abundance of *Neisseria*, *Capnocytophaga*, *Veillonella*, *Rhodococcus*, *Jensenia*, and *Corynebacterium*. Functional predictions suggested shifts in local metabolic pathways. Transcriptomic analysis revealed reprogramming of metabolism-related genes in the lens capsule and activation of downstream signaling pathways including ErbB and HIF-1, which are associated with lens epithelial cell apoptosis.

**Discussion:**

These findings suggest that alterations in the aqueous humor microbial community may be associated with the lens metabolic microenvironment and apoptotic pathway activation, thereby linking to cataract progression in high myopia. This study provides a novel perspective on the pathogenesis of high myopia-associated cataract.

## 1 Introduction

Cataract, as the leading cause of blindness worldwide, poses a significant challenge to public vision health (1, 2). Notably, high myopia is a critical risk factor that significantly advances the age of cataract onset and increases surgical difficulty and postoperative complication rates (3–5). With the rising global prevalence of myopia, the clinical management of high myopia complicated by cataract has become an increasingly formidable challenge in ophthalmology, imposing not only a substantial vision burden on patients but also tremendous pressure on healthcare resources (6). Therefore, an in-depth investigation into the unique pathological mechanisms of cataract development in the context of high myopia is urgently needed to develop targeted early intervention strategies and improve patient outcomes.

Although cataract surgical techniques have matured, patients with high myopia present specific anatomical alterations, such as significant axial elongation, zonular laxity (7, 8), and increased capsular bag volume, which complicate the surgical procedure and elevate the risk of postoperative complications, including capsular contraction syndrome and intraocular lens dislocation (9, 10). These clinical features strongly suggest that the pathophysiology of high myopia-related cataract may differ considerably from that of typical age-related cataract. However, the molecular mechanisms driving this process remain poorly understood. Traditional research perspectives have predominantly focused on host genetic, metabolic, or oxidative stress factors (11–15), which fail to fully explain its distinct clinical progression.

In recent years, the rise of microbiome research has offered a novel perspective for understanding the pathogenesis of complex diseases (16–18). Accumulating evidence links microbial communities in sites such as the gut and oral cavity to various systemic diseases (19, 20). However, research on the microbial composition and function in the traditionally considered “sterile” intraocular environment, particularly the aqueous humor—a key intraocular fluid—remains in its infancy. Meanwhile, axial elongation and scleral remodeling associated with high myopia, along with potential changes in blood-aqueous barrier function, could theoretically create a unique microenvironment conducive to microbial colonization or functional alteration (21). Studies have already observed altered pro-inflammatory cytokine profiles in the aqueous humor of patients with high myopia and cataract, suggesting an imbalance in intraocular immune and inflammatory status (21, 22). Potential interactions may exist between these factors and the lens’s own microenvironment and cellular function, yet research integrating the intraocular microbiome with host local tissue response is currently lacking.

To address this gap, our study employs a multi-omics integration strategy to elucidate the potential mechanisms of high myopia-associated cataract. We simultaneously applied 2bRAD-M gene sequencing and transcriptome sequencing (RNA-seq). 2bRAD-M sequencing enables unbiased, high-throughput characterization of the overall structure, diversity, and species composition of microbial community (23). Conversely, RNA-seq comprehensively reveals the genome-wide expression profile changes in lens capsule tissue, providing a molecular map of host cellular functional reprogramming in the disease state (24). By integrating these datasets for correlation analysis, we aim to construct a potential “aqueous humor microbiome-lens transcriptome” interaction network, thereby opening new avenues for elucidating how microbial communities correlate with local tissue homeostasis.

This exploratory pilot study aims to identify correlational patterns between aqueous humor microbiome and HMC progress by achieving the following core objectives: First, to systematically compare the differences in diversity, community structure, and abundance of specific genera in the aqueous humor microbiome between patients with high myopia and cataract and those with age-related cataract. Second, through transcriptomic analysis, to reveal the genome-wide expression landscape of lens capsule tissue in the context of high myopia, particularly pathways related to metabolism, stress, and cell fate determination, and to explore potential associations between specific microbial features and host gene expression patterns, thereby suggesting a possible mechanistic chain of microbial influence on lens function. This study not only promises to deepen the understanding of the pathogenesis of high myopia-related cataract but may also provide a theoretical basis for future development of novel intervention strategies based on microbiome modulation or targeting specific host signaling pathways.

## 2 Materials and methods

### 2.1 Sample collection

This study was a prospective cohort study approved by the Ethics Committee of Shanghai Tenth People’s Hospital, with all patients providing informed consent. Inclusion criteria for the high myopia cataract (HMC) group: age < 80 years, axial length ≥ 26.5 mm, undergoing cataract surgery at our hospital. Inclusion criteria for the age-related cataract (ARC) group: age < 80 years, axial length 22.0–24.5 mm, no myopia or myopia ≤ −3.00 D, no systemic or ocular comorbidities, undergoing cataract surgery at our hospital. Patients with recent ocular surgery, inflammation, trauma, systemic autoimmune diseases, or long-term use of antibiotics/immunosuppressants were strictly excluded. Before cataract surgery, 100–150 μl of aqueous humor was collected via corneal paracentesis under sterile conditions, immediately placed in sterile, enzyme-free Eppendorf tubes, and stored at −80 °C. Simultaneously, the anterior lens capsule was collected during continuous curvilinear capsulorhexis, rinsed with PBS, and stored at −80°C. A total of 12 aqueous humor samples were collected: six from the high myopia cataract group and six from the ARC group; six lens capsule samples were collected: three from the high myopia group and 3 from the ARC group.

### 2.2 Aqueous humor microbiota library construction and sequencing

Library construction and sequencing were performed following the original 2bRAD-M protocol developed by Wang et al. (3) with minor modifications. DNA used in this study was extracted using the MagPure Soil DNA KF Kit under P2 laboratory conditions. DNA (1 pg–200 ng) was digested with 4 U of the enzyme BcgI (NEB) for 3 h at 37 °C. Subsequently, adapters were ligated to the DNA fragments. The ligation reaction was performed by combining 10 μl of digested DNA with 10 μl of a ligation master mix containing 0.2 μM each of two adapters and 800 U T4 DNA ligase (NEB). Ligation was carried out at 4 °C for 12 h. Then, ligation products were amplified, and PCR products were subjected to 8% polyacrylamide gel electrophoresis. Bands of approximately 100 bp were excised from the polyacrylamide gel, and DNA was eluted from the gel in nuclease-free water for 6–12 h at 4 °C. Sample-specific barcodes were introduced by PCR with platform-specific barcode-bearing primers. Each 20 μl PCR contained 6 μl of gel-extracted PCR product, 0.2 μM of each primer, 0.3 mM dNTPs, 1 × Phusion HF buffer, and 0.4 U Phusion high-fidelity DNA polymerase (NEB). PCR products were purified using the QIAquick PCR purification kit (Qiagen) and then sequenced on the Illumina Nova PE150 platform. 2bRAD-M was performed at Qingdao Oebiotech Co., Ltd. (Qingdao, China).

### 2.3 Identification of species-specific 2bRAD-M markers from a comprehensive genome database

First, a total of 404,199 microbial genomes (including bacteria, fungi, and archaea) were downloaded from the GTDB and Ensembl databases. Then, built-in Perl scripts were used to sample restriction fragments from microbial genomes using each of 16 type 2B restriction enzymes, forming a comprehensive 2bRAD microbial genome database. The set of 2bRAD tags sampled from each genome was assigned under the corresponding GCF number, along with the taxonomic information of the GCF corresponding to the whole genome. Finally, all 2bRAD tags from each GCF occurring once within the genome were compared against all others. Those 2bRAD tags specific to a species-level taxon (having no overlap with other species) were developed as species-specific 2bRAD markers, collectively forming the 2bRAD marker database.

### 2.4 Calculation of relative abundance and quality control

First, to identify microbial species within each sample, all sequenced 2bRAD tags after quality control were mapped (using a built-in Perl script) against the 2bRAD marker database containing all theoretically unique 2bRAD tags for each of 86,022 microbial species in the database. To control for false positives in species identification, a G score was derived for each species identified within a sample as follows, representing the harmonic mean of read coverage of 2bRAD markers belonging to a species and the number of all possible 2bRAD markers for that species. The G score threshold for false positive discovery of a microbial species was set at 5.


$${\mathrm{Gscore}}_{\mathrm{species}\,i}=\sqrt{S_{i}\times t_{i}}$$

S: number of reads assigned to all 2bRAD markers belonging to species i within a sample.

t: number of all 2bRAD markers of species i sequenced within a sample.

Then, the average read coverage of all 2bRAD markers for each species was calculated, representing the number of individuals belonging to a species present in the sample at a given sequencing depth. The relative abundance of a given species is then calculated as the ratio of the number of microbial individuals belonging to that species to the total number of individuals from known detectable species within the sample.


$$\mathrm{Relative}\,{\mathrm{abundance}}_{\mathrm{species}\,i}=\frac{S_{i}/T_{i}}{\sum_{i=1}^{n}S_{i}/T_{i}}$$

S: number of reads assigned to all 2bRAD markers of species i within a sample.

T: number of all theoretical 2bRAD markers for species i.

Given the low-biomass nature of aqueous humor, we set up negative controls for identifying and removing contaminating sequences from the experimental samples. This process employed a sampling-ratio-based method to balance the quantitative relationship between the samples and the controls. Specifically, based on the ratio of the number of clean reads in a sample to the number of clean reads in the negative control, a certain number of sequences were randomly drawn from the negative control pool. For each sequence in the sample, we checked whether the sequence itself or its reverse complement was present in the sampled negative control sequences. If a match was found, the sequence was considered a contaminant and removed; otherwise, it was retained, ultimately yielding the contamination-removed sample sequences.


$$P=\frac{N_{S}}{N_{C}+N_{S}}$$

P: sampling ratio.

N_*S*_: number of clean reads in the sample.

N_*C*_: number of clean reads in the negative control.

### 2.5 Microbial diversity and functional prediction analysis

Assessments included alpha diversity, beta diversity, and species composition differences. Alpha diversity (Chao1) were calculated. Beta diversity was explored using PCA on the normalized/rarefied species abundance matrix. Global between-group differences were evaluated using PERMANOVA. Functional prediction was performed using PICRUSt2 (version 2.3.0b0), a software tool that predicts functional abundances based on marker gene sequences. The abundances of COG (Clusters of Orthologous Groups) and KO (KEGG Orthology) functional genes were predicted for each sample using default parameters.

### 2.6 RNA isolation and library preparation

Total RNA from patients’ lens capsule was extracted using TRIzol reagent (Invitrogen, CA, United States) according to the manufacturer’s protocol. RNA purity and quantification were evaluated using the NanoDrop 2000 spectrophotometer (Thermo Scientific, United States). RNA integrity was assessed using the Agilent 2100 Bioanalyzer (Agilent Technologies, Santa Clara, CA, United States). Libraries were constructed using the VAHTS Universal V6 RNA-seq Library Prep Kit according to the manufacturer’s instructions. Transcriptome sequencing and analysis were conducted by OE Biotech Co., Ltd. (Shanghai, China).

### 2.7 RNA sequencing and differentially expressed genes analysis

Libraries were sequenced on the Illumina NovaSeq 6000 platform, generating 150 bp paired-end reads. Raw reads in FASTQ format were first processed using fastp to remove low-quality reads and obtain clean reads. Clean reads were mapped to the reference genome using HISAT2. FPKM for each gene was calculated, and read counts for each gene were obtained using HTSeq-count. PCA was performed using R (v 3.2.0) to evaluate sample biological duplication.

Differential expression analysis was performed using DESeq2. A Q value < 0.05 and fold change > 2 or < 0.5 were set as the thresholds for significantly differentially expressed genes (DEGs). Hierarchical cluster analysis of DEGs was performed using R (v 3.2.0) to demonstrate gene expression patterns across groups and samples. A radar map of the top 30 genes was drawn using the R package ggradar to show expression of up- or down-regulated DEGs. Based on the hypergeometric distribution, GO, KEGG, Reactome, and WikiPathways enrichment analyses of DEGs were performed to screen for significantly enriched terms using R (v 3.2.0). R (v 3.2.0) was used to draw column diagrams, chord diagrams, and bubble diagrams of significant enrichment terms.

### 2.8 Statistical analysis

Differences between the two groups were compared using the Wilcoxon rank-sum test. For functional predictions using PICRUSt2, including KEGG and COG pathway analyses, intergroup comparisons were also performed using the Wilcoxon test. All tests were two-sided, and a *P*-value < 0.05 was considered statistically significant.

## 3 Results

### 3.1 Microbial community diversity and composition in HMC and ARC groups

To investigate microbial community structure alterations associated with pathological myopia (PM), we performed high-throughput sequencing and analyzed the diversity and composition of the microbiota in HMC patients and matched ARC patients.

To evaluate the sampling adequacy of the current study, we first performed a species accumulation curve analysis. We observed that as the sample size increased, the curve showed an initial upward trend and then gradually leveled off, reaching a plateau (Supplementary Figure 1), meaning that the current sampling has essentially covered the major species in the aqueous humor microbial community.

**FIGURE 1:**
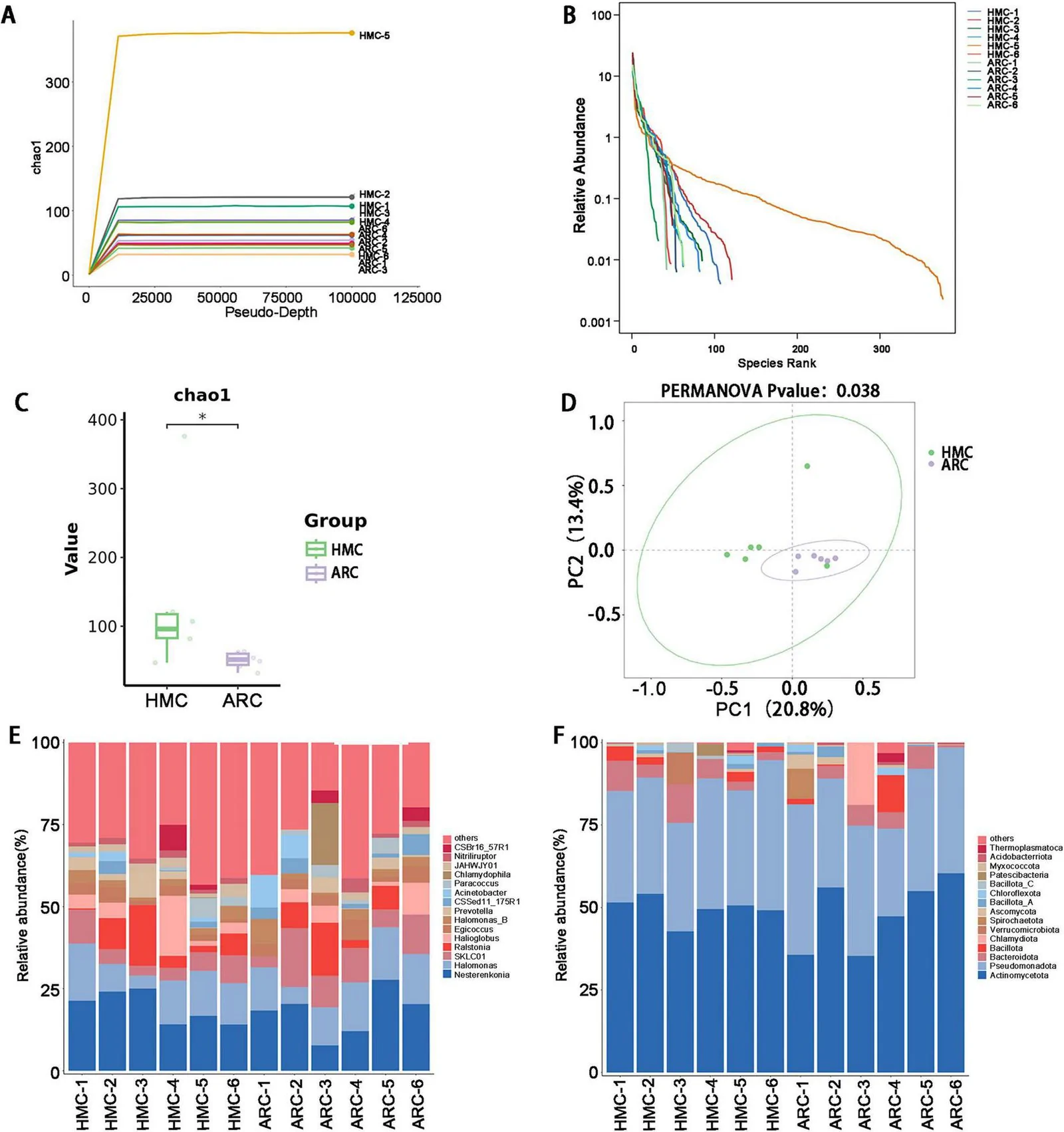
Microbial community diversity and composition in high myopia cataract (HMC) and age-related cataract (ARC) groups. **(A)** Rarefaction curves showing the observed number of operational taxonomic units (OTUs) as a function of sequencing depth (pseudo-depth) for each sample. **(B)** Rank-abundance curves illustrating the relative abundance distribution of OTUs across samples. **(C)** Alpha diversity comparison based on Chao1 richness estimator between HMC and ARC groups (*P* = 0.026). Box plots display the median, quartiles, and range. **(D)** Beta diversity analysis via principal coordinate analysis (PCoA) based on Bray-Curtis dissimilarity. Each point represents a sample; percentages on axes indicate the variation explained. PERMANOVA test revealed a significant difference between groups (*p* = 0.038). **(E)** Relative abundance of major bacterial in genus level in each sample. **(F)** Relative abundance of major bacterial in phylum level in each sample. Samples are ordered by group: ARC-1 to ARC-6 (*n* = 6) and HMC-1 to HMC-6 (*n* = 6). *Stands for significant difference existed between groups; *P< 0.05.

Rarefaction curves were generated to assess sequencing depth saturation. As shown in Figure 1A, the curves for all samples approached a plateau, indicating sufficient sequencing depth to capture the majority of microbial diversity. The rank-abundance curves (Figure 1B) reflected species richness and evenness across samples, revealing differences in community structure between the two groups.

Alpha diversity was evaluate using the Chao1 richness index. The HMC group exhibited a significant increase in microbial richness compared to the ARC group (Figures 1C, *P* = 0.026). Beta diversity was evaluated by principal coordinate analysis (PCoA) based on Bray-Curtis dissimilarity. The PCoA plot showed a clear separation between HMC and ARC samples (Figure 1D), with the first two principal components explaining 20.8% and 13.4% of the total variation, respectively. PERMANOVA analysis confirmed that microbial community composition differed significantly between the two groups (*p* = 0.038).

At the taxonomic level, the relative abundances of major bacterial genera and phyla were examined. Figure 1E illustrates the distribution of predominant genera in each sample, while Figure 1F shows the community composition at the phylum level. Notable shifts in specific taxa were observed between the HMC and ARC groups, suggesting that HMC is associated with distinct alterations in microbial community structure.

### 3.2 Relative abundance of the bacterial community in HMC and ARC groups

**FIGURE 2:**
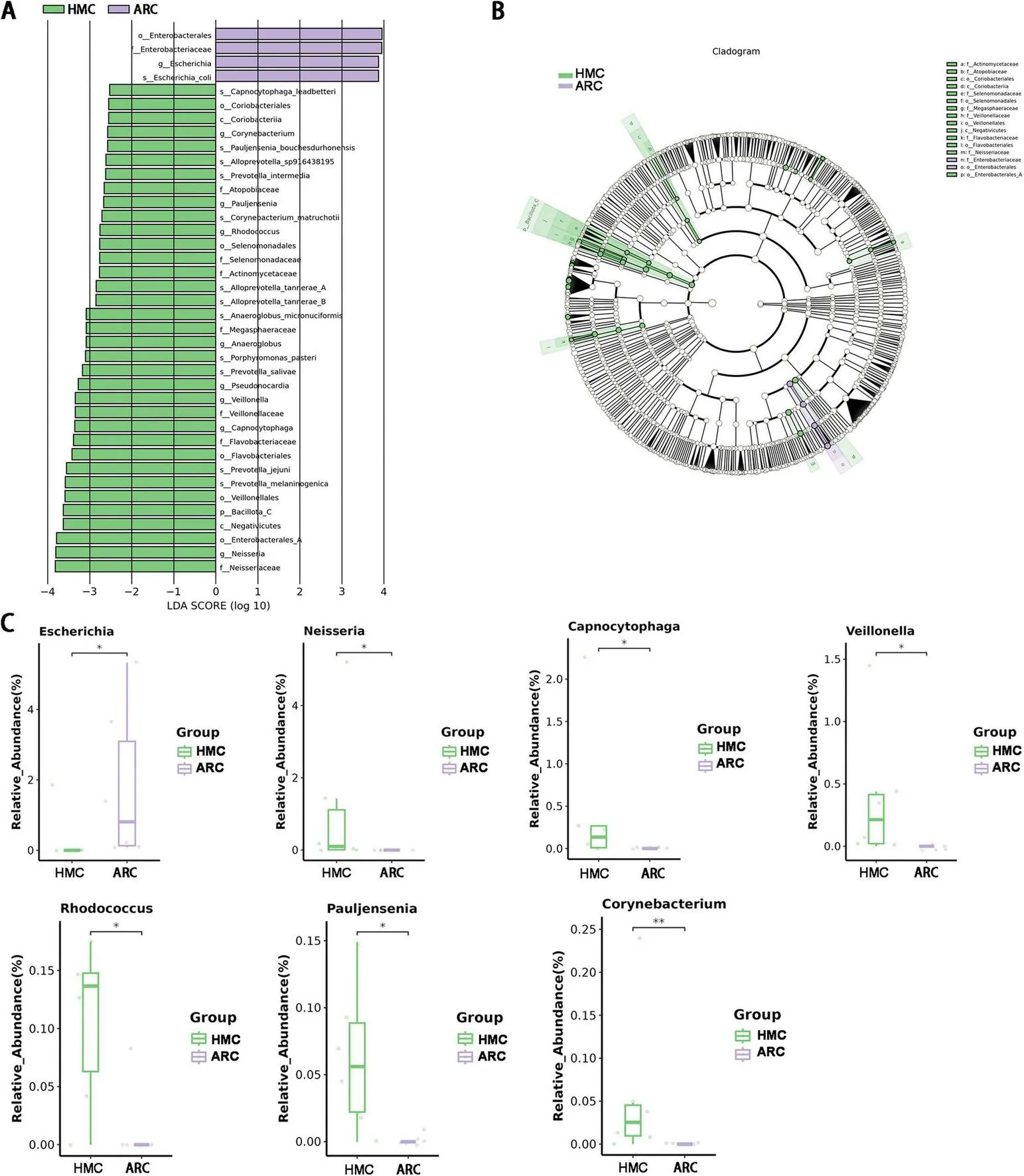
Relative abundance of the bacterial community in patients with high myopia cataract (HMC) and age-related cataract (ARC). **(A)**. LEfSe analysis showed the relative abundance of genera in the HMC and ARC groups. A total of 35 bacterial taxa showed significant differences in relative abundance, with 4 and 35 distinct microbial taxa in the HC and DR groups, respectively (LDA score > 2.5, *P* < 0.05, Kruskal–Wallis test). **(B)** LEfSe analysis of the aqueous humor microbiota composition from the phylum to the genus level in the two groups. The cladogram displayed the correlations between taxa at different taxonomic levels. Each circle represents a hierarchy, followed by phylum, class, order, family, and genus. **(C)** Comparison of differentially abundant bacterial genera between groups. *Stands for significant difference existed between groups; *P < 0.05, **P < 0.01.

LEfSe analysis (LDA score > 2.5, *P* < 0.05, Kruskal-Wallis test) was performed to assess differences in the aqueous humor microbiota between the HMC and ARC groups. A total of 35 bacterial taxa showed significant differences in relative abundance between the two groups. Among them, 4 distinct microbial taxa were enriched in the ARC group, while 35 were enriched in the HMC group (Figure 2A). The cladogram illustrated the taxonomic relationships from the phylum to the genus level, revealing the phylogenetic distribution and group-specific differences across taxonomic hierarchies (Figure 2B). Comparative analysis at the genus level further highlighted significant alterations in the abundance of multiple bacterial genera between the HMC and ARC groups, including *Escherichla, Neisseria, Capnocytophaga, Veillonella, Rhodococcus, Pauljensenia*, and *Corynebacterium* (Figure 2C). Collectively, these findings indicate a marked remodeling of the aqueous humor microbiota in patients with pathological myopia, which may be closely associated with changes in the local microenvironment.

### 3.3 Functional predictions of the microbial community in pathological myopia and ARC groups

**FIGURE 3:**
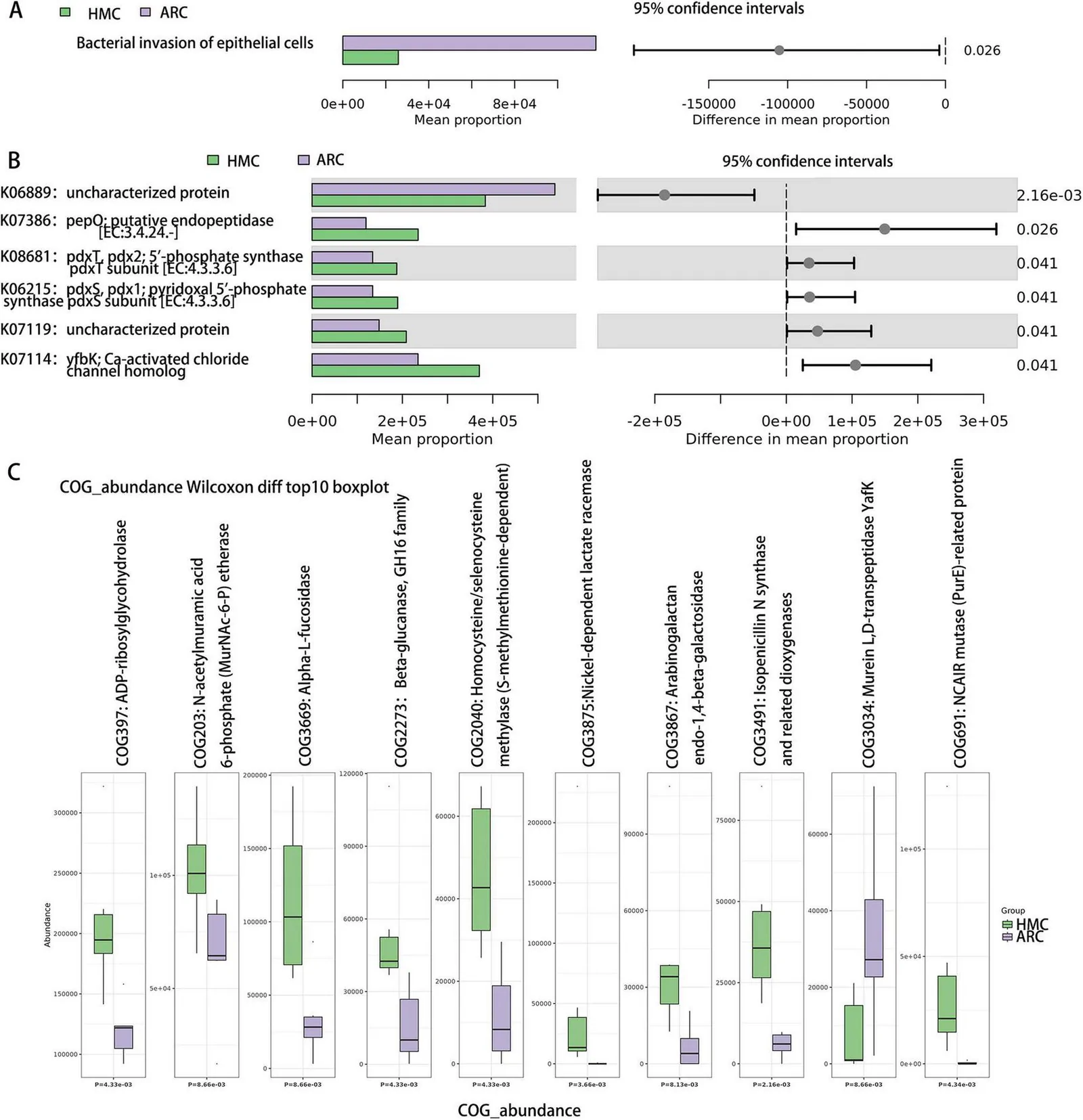
Functional prediction of microbial communities based on Kyoto Encyclopedia of Genes and Genomes (KEGG) and Clusters of Orthologous Groups (COG) analyses. **(A)** The significantly differential pathway at KEGG level 3 between two groups. **(B)** Predicted changes in functional genes based on KEGG orthology analysis. **(C)** Predicted changes in protein functions based on COG analysis.

To explore potential functional implications of the altered microbial community associated with HMC, we performed Phylogenetic Investigation of Communities by Reconstruction of Unobserved States (PICRUSt2) to predict functional profiles based on 2bRAD-M sequencing data. Predicted functions were annotated using the Kyoto Encyclopedia of Genes and Genomes (KEGG) and Clusters of Orthologous Groups (COG) databases. KEGG enrichment analysis revealed significant differences mainly related to microbial energy metabolism between the HMC and ARC groups (Figures 3A, B). COG analysis was performed to predict changes in protein functions (Figure 3C). Several COG categories, including amino acid transport and metabolism, carbohydrate transport and metabolism, and translation, ribosomal structure, and biogenesis, showed significant differences between HMC and ARC patients.

### 3.4 Metabolic alterations in lens capsules of pathological myopia and age-related cataract patients

To investigate metabolic changes associated with HMC in the context of cataract formation, we performed RNA-seq analysis on lens capsule tissues from patients with HMC and ARC. Functional enrichment and expression profiling were conducted to identify differentially regulated metabolic pathways and genes.

**FIGURE 4:**
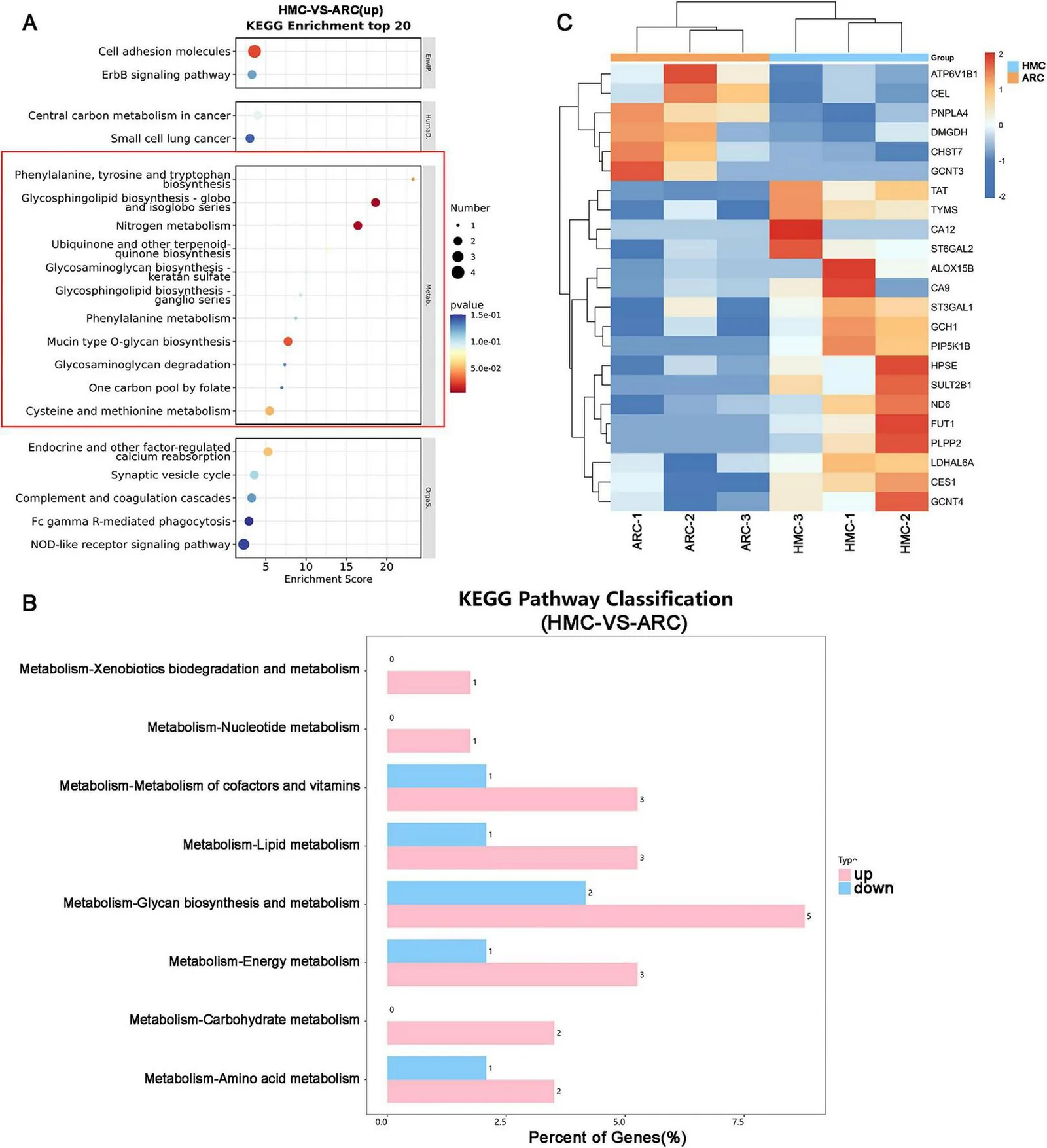
Metabolic alterations in the lens capsules of patients with pathological myopia and age-related cataracts. **(A)** Kyoto Encyclopedia of Genes and Genomes (KEGG) pathway enrichment analysis based on RNA-seq data from cataract lens capsules. Upregulated differentially expressed pathways are shown; metabolism-related pathways are highlighted with red squares. **(B)** Changes in the number of genes associated with metabolic pathways between groups. **(C)** Heatmap displaying the expression profiles of metabolism-related genes, illustrating the specific changes between pathological myopia and age-related cataract samples.

KEGG pathway enrichment analysis revealed that multiple biological pathways were upregulated in the HMC group compared to the ARC group (Figure 4A). Among these, metabolism-related pathways—including those involved in carbohydrate, lipid, and amino acid metabolism—were prominently enriched (highlighted with red squares), suggesting enhanced metabolic activity in the lens capsules of HMC patients.

Consistent with this observation, the number of genes associated with metabolic pathways was significantly altered between the two groups (Figure 4B). The HMC group exhibited a greater number of differentially expressed genes involved in metabolic processes, indicating a broad shift in the metabolic landscape of the lens capsule in pathological myopia.

To further characterize these changes, we generated a heatmap displaying the expression profiles of metabolism-related genes (Figure 4C). Unsupervised clustering based on these genes clearly separated HMC samples from ARC samples, demonstrating distinct metabolic gene signatures between the two groups. Several key metabolic enzymes and regulators showed marked upregulation or downregulation in the HMC group, underscoring the metabolic reprogramming occurring in the lens capsule under pathological myopic conditions.

### 3.5 Cell death-related pathway alterations and gene interaction networks in lens capsules

To explore the potential involvement of cell death mechanisms in HMC, we analyzed transcriptomic data from lens capsules of HMC and ARC patients, focusing on cell death-related pathways and gene interactions.

**FIGURE 5:**
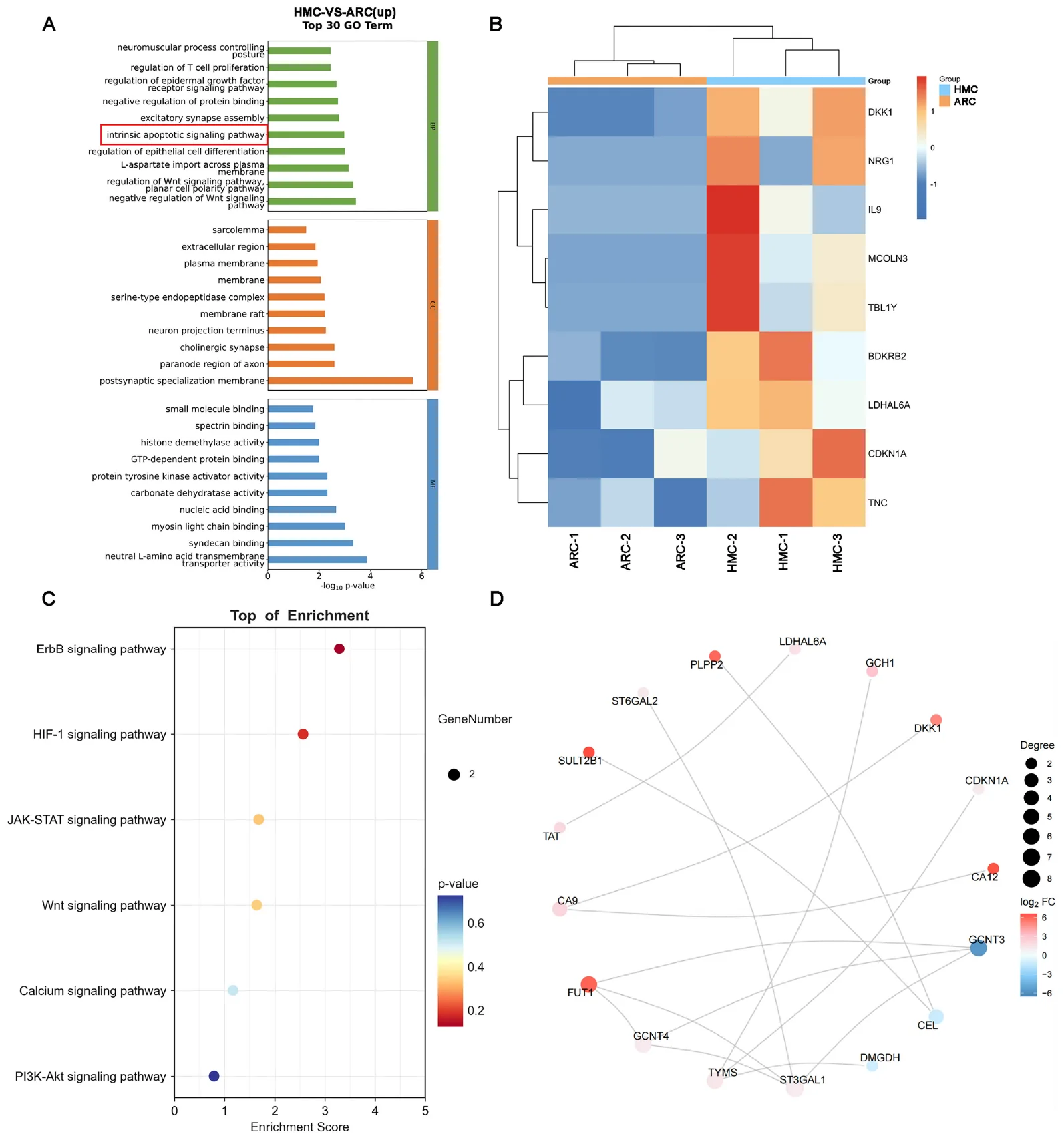
Pathway analysis and gene interaction networks associated with cell death in lens capsules. **(A)** Gene Ontology (GO) enrichment analysis showing upregulated pathways in cataract lens capsules, with the intrinsic apoptotic pathway potentially significantly altered. **(B)** Heatmap depicting the expression changes of cell death-related genes between the two patient groups. **(C)** Bubble plot illustrating the alterations of signaling pathways in lens capsules. **(D)** Protein-protein interaction (PPI) network of differentially expressed genes potentially involved in relevant pathways.

Gene Ontology (GO) enrichment analysis revealed that multiple biological processes were upregulated in the HMC group, with the intrinsic apoptotic pathway showing potentially significant alterations (Figure 5A). Differential expression patterns of cell death-related genes were visualized using a heatmap, which demonstrated distinct clustering between HMC and ARC samples (Figure 5B). Bubble plot analysis of signaling pathways revealed significant alterations in pathways associated with apoptosis, including ErbB and HIF-1 signaling pathways (Figure 5C). These findings indicate that multiple interconnected signaling cascades may be connected with cell death regulation in the lens capsule.

Finally, protein-protein interaction (PPI) network analysis identified key hub genes and their interactions among differentially expressed genes potentially involved in relevant pathways (Figure 5D). The network highlighted central nodes, including FUT1, suggesting potential regulatory mechanisms underlying cell death in HMC formation.

## 4 Discussion

High myopia is a significant risk factor that advances the age of cataract onset and increases surgical difficulty and postoperative complication rates, posing a substantial burden on global public health (3). Although surgery is the primary treatment for high myopia complicated by cataract, understanding of its distinct pathological mechanisms compared to typical age-related cataract remains insufficient, limiting the development of early intervention or progression delay strategies for this condition. Therefore, exploring its pathogenesis from novel perspectives holds significant clinical importance for identifying new intervention targets.

This study integrated intraocular microbiome analysis with host local tissue transcriptome analysis to elucidate the pathological process of high myopia-associated cataract. Using 2bRAD-M sequencing and RNA-seq, we systematically compared the aqueous humor microbial community structure and lens capsule gene expression profiles between patients with high myopia and cataract and those with age-related cataract. Preliminary results indicate significant alterations in aqueous humor microbial diversity and specific genus composition in the high myopia group, alongside metabolic reprogramming and activation of apoptosis-related signaling pathways in the lens capsule tissue. Firstly, we observed significantly altered microbial diversity in the aqueous humor of patients with high myopia and cataract (Figure 1), a finding paralleling observations of microbial dysbiosis in other chronic eye diseases such as age-related macular degeneration (25). However, unlike studies on ocular surface sites like the conjunctiva, the origin and homeostasis maintenance mechanisms of the microbial community in the aqueous humor, as part of the intraocular microenvironment, are more complex (26). Notably, axial elongation and scleral remodeling accompanying high myopia may lead to blood-aqueous barrier dysfunction, potentially creating conditions for distal microbes from sites like the gut or oral cavity to colonize via hematogenous migration or reduced local immune surveillance (27). The markedly increased abundance of *Neisseria* and *Veillonella* in our study is particularly significant (Figure 2); these genera are common in oral and respiratory microbiomes (28–30), and their increase may reflect a breakdown of intraocular immune tolerance or altered local metabolic substrates (31). Conversely, the decrease in common commensals like *Escherichia* may weaken their competitive inhibition against opportunistic pathogens, collectively exacerbating intraocular microecological imbalance. This distinct microbial signature differs notably from patterns observed in simple dry eye disease, where the conjunctival microbiome is predominantly *Proteobacteria* and influenced by age, suggesting high myopia-associated cataract may have a specific intraocular dysbiosis pattern (32).

Furthermore, functional prediction of differential microbiota implied widespread alterations in the metabolic potential of the high myopia group’s microbial community, particularly in pathways related to energy metabolism, glucose metabolism, and amino acid metabolism (Figure 3). This advances our observation from species composition to functional activity, akin to associations between specific metabolic pathways and host phenotypes revealed by functional prediction in gut microbiome studies (33). Previous research indicates that gut microbes remotely regulate host physiology by producing metabolites like short-chain fatty acids (34, 35). In the current pilot study, we cannot directly establish a causal link between the microbiome and the transcriptome related pathways. Nevertheless, based on our findings and the aforementioned literature, we speculate that alterations in microbial metabolic pathways may interact with systemic metabolic abnormalities. This “remote correlation” between microbial function and host metabolic state, i.e., “aqueous humor microbiome-lens” interaction, may be mediated through metabolite exchange.

At the host level, transcriptomic analysis of the lens capsule confirmed significant expression changes in metabolism-related genes and activation of apoptotic processes in high myopia cataract (Figures 4, 5). This aligns with previous studies conclusively demonstrating oxidative stress-induced lens epithelial cell apoptosis as a core mechanism of cataractogenesis (36). In our high myopia cataract cohort, these changes may be linked to microenvironmental metabolic alterations caused by shifts in microbial community structure. For example, alterations in energy or amino acid metabolism induced by the aqueous humor microbiota might compromise lens epithelial cell energy supply, rendering them more susceptible to oxidative damage; or disturbances in lens epithelial cell lipid metabolism could directly affect lens cell membrane stability and transparency (37). These metabolic dysregulations collectively form the molecular basis predisposing lens epithelial cells to apoptosis, potentially partially explaining the clinically observed faster cataract progression in high myopia patients (3).

Importantly, KEGG pathway enrichment analysis revealed activation of ErbB and HIF-1 signaling pathways in the lens capsule of the high myopia group (Figure 5C). The ErbB pathway is typically involved in cell proliferation, differentiation, and survival; its aberrant activation in the cataract context may have dual implications: on one hand, it could represent a reparative cellular response to injury; on the other hand, persistent activation might ultimately lead to apoptosis (38). HIF-1 pathway activation strongly suggests the presence of hypoxia or a hypoxia-like metabolic state locally within the lens, consistent with the hypothesis of altered lens epithelial cell metabolism under high myopia conditions (21, 39, 40). Alterations in this pathway can also induce cell apoptosis(41, 42). This study links these pathways to high myopia cataract and speculate that their activation may arise not only from the host’s pathological changes (e.g., hypoxia) but also be associated with alterations in the aqueous humor microbiome. For instance, metabolites produced by certain bacteria, such as succinate, have been identified as HIF-1α stabilizers, providing a plausible mechanistic link for microbial metabolite-mediated cross-kingdom regulation of host cell signaling pathways (43).

This study has several limitations. First, the sample size was relatively limited, particularly for lens capsule samples, which may lead to insufficient statistical power and potentially mask intra-group heterogeneity or more subtle association signals, affecting the robustness and external generalizability of the findings. Thus, validation would be required in larger independent cohorts in the future research. Second, this study primarily reveals potential correlations between the microbiome and transcriptome. However, limited by its observational design and lack of functional validation, causal direction cannot be established, necessitating further experimental elucidation. Besides, the changes in metabolic pathways of the aqueous humor microenvironment caused by microorganisms, as predicted by PICRUSt2, require further direct metabolomic or experimental validation.

## 5 Conclusion

In summary, through multi-omics integrative analysis, this study reveals a potential pattern in the intraocular environment of patients with high myopia and cataract. This pattern includes aqueous humor dysbiosis and its predicted functional metabolic alterations, which potentially correlate with lens capsule metabolic reprogramming and the activation of pro-apoptotic signaling pathways such as ErbB/HIF-1. This finding provides a new perspective for understanding non-traditional pathogenic mechanisms of this disease. Future research should focus on validating key associations in expanded cohorts and utilizing co-culture systems or animal models to directly verify the impact of specific microbial genera or their metabolites on lens epithelial cell function, thereby advancing translation toward clinical biomarkers or intervention targets.

## Data Availability

The original contributions presented in the study are included in the article/Supplementary material, further inquiries can be directed to the corresponding author/s.
