# Correction: Integrated analysis of the aqueous humor microbiome and lens capsule transcriptome in high myopia cataract: a pilot study

**DOI:** 10.3389/fmed.2026.1939507

**Published:** 2026-08-06

**Authors:** Yaoyan Qiu, Wenting Cai, Zihan Xie, Chenxi Li, Fan Yang, Yiyong Qian, Jingwu Song, Tianyu Zheng

**Affiliations:** 1 Department of Ophthalmology, Shanghai Tenth People's Hospital, Tongji University School of Medicine, Shanghai, China; 2 Visual Rehabilitation Center, Tongji University School of Medicine, Shanghai, China; 3 Tongji Eye Institute, Tongji University School of Medicine, Shanghai, China; 4 Department of Ophthalmology, Eye and ENT Hospital, Fudan University, Shanghai, China; 5 Eye Institute, Eye and ENT Hospital, Fudan University, Shanghai, China; 6 NHC Key Laboratory of Myopia and Related Eye Diseases, Shanghai, China; 7 Shanghai Key Laboratory of Visual Impairment and Restoration, Shanghai, China; 8 Department of Functional Intestinal Diseases, General Surgery of Shanghai Tenth People's Hospital, Tongji University School of Medicine, Shanghai, China; 9 Shen 1001 Life Institute, Shanghai, China

**Keywords:** apoptosis, aqueous humor microbiome, cataract, high myopia, lens capsule, multi-omics integration, transcriptome

There was a mistake in Figure 5 as published. The heatmap in Figure 5B was inadvertently duplicated from Figure 4C and does not represent the intended experimental data. The corrected Figure 5 appears below.

**Figure 5:**
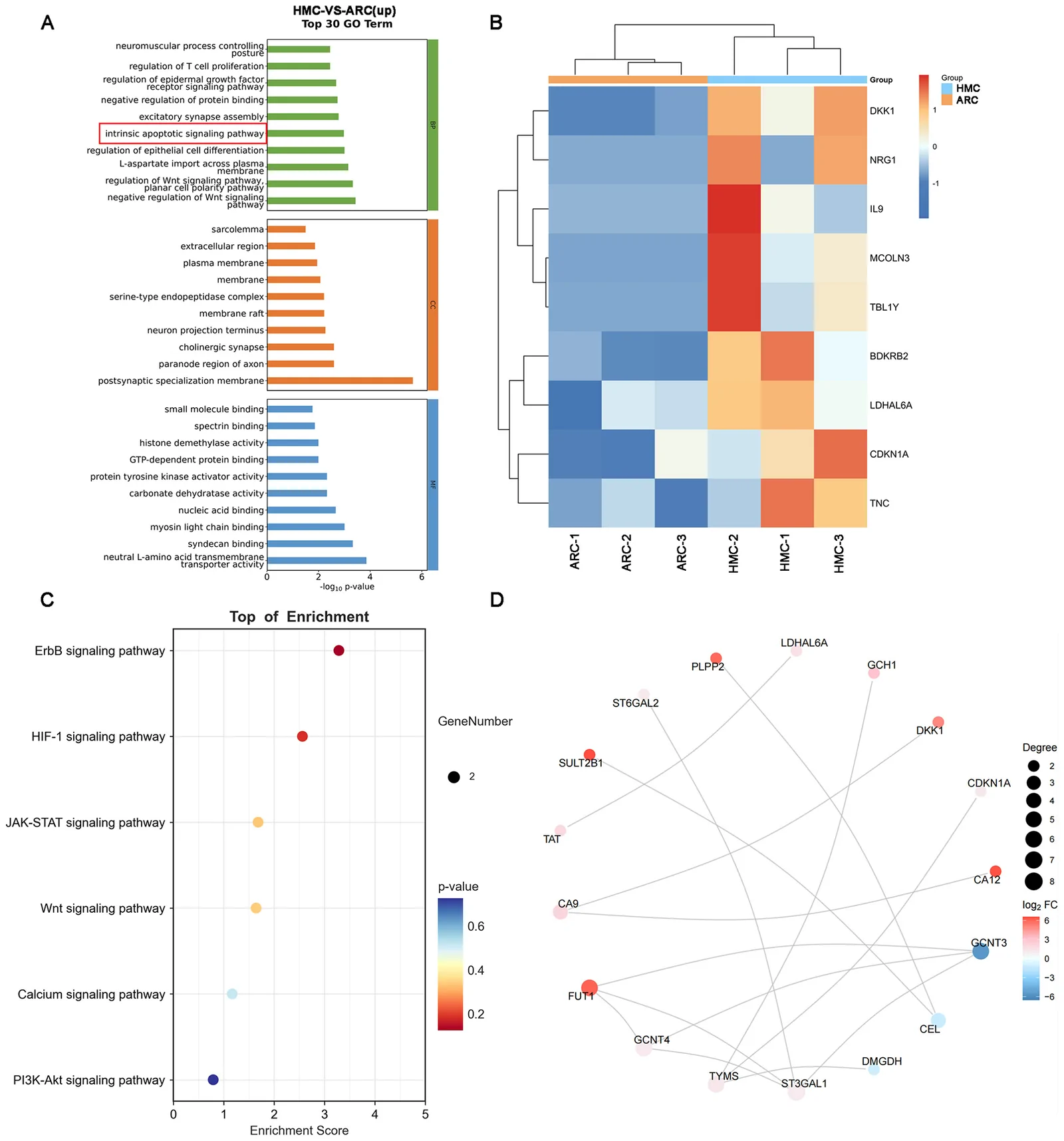
Pathway analysis and gene interaction networks associated with cell death in lens capsules. **(A)** Gene Ontology (GO) enrichment analysis showing upregulated pathways in cataract lens capsules, with the intrinsic apoptotic pathway potentially significantly altered. **(B)** Heatmap depicting the expression changes of cell death-related genes between the two patient groups. **(C)** Bubble plot illustrating the alterations of signaling pathways in lens capsules. **(D)** Protein-protein interaction (PPI) network of differentially expressed genes potentially involved in relevant pathways.

The original version of this article has been updated.

