# Supplementary material for: Integrated analysis of the aqueous humor microbiome and lens capsule transcriptome in high myopia cataract: a pilot study
Source: Front Med (Lausanne). 2026 Jun 16;13:1845205. doi: 10.3389/fmed.2026.1845205 (PMC13314463; doi:10.3389/fmed.2026.1845205)
Supplement: Supplementary file 2 [file Table_1.docx]

Supplementary Material

**Supplementary Figure 1.** Species accumulation curve. The horizontal axis represents the sample size (number of subjects), and the vertical axis represents the number of detected species (number of operational taxonomic units / amplicon sequence variants).
