# Supplementary material for: Integrated analysis of the aqueous humor microbiome and lens capsule transcriptome in high myopia cataract: a pilot study
Source: Front Med (Lausanne). 2026 Jun 16;13:1845205. doi: 10.3389/fmed.2026.1845205 (PMC13314463; doi:10.3389/fmed.2026.1845205)
Supplement: Supplementary file 3 [file Data_Sheet_1.zip › 1.Community_Structure/Krona/C372089/Krona.html]

Javascript must be enabled to view this page.

magnitude
magnitudeUnassigned

control-2
control-1
control-3
control-4
control-6
control-7
case-1
case-2
case-3
case-4
case-5
case-7

99999710000009999991000004100000310000031000001100000199999910000039999991000002

996732989073999999100000498801710000039575219780919999999671329999991000002

075903605517290000000

075903605500000000

075903605500000000

075903605500000000

075903605500000000

0003605500000000

07590000000000

000017290000000

000017290000000

000017290000000

000017290000000

000017290000000

000065880000000

000065880000000

000065880000000

000065880000000

000065880000000

000065880000000

00002241000188746000

00002241000188746000

00002241000188746000

00002241000188746000

00002241000188746000

00002241000188746000

0000185301052001168000

0000185301052001168000

0000185301052001168000

0000001052001168000

0000001052001168000

0000000001168000

000000105200000

000018530000000

000018530000000

000018530000000

437132260600298111724916458426001124950385

437132260600298111724916458426001124950385

0000669101560100000

00002310000000

00002310000000

00002310000000

0000477201560100000

000047720000000

000013800000000

000033920000000

0000001560100000

0000001560100000

000016880000000

000016880000000

000016880000000

022606002204172490002113200

022606002204172490002113200

022606002204172490002113200

022606002204172490002113200

4371300052710000000

4371300052710000000

4371300052710000000

4371300000000000

000052710000000

000018580000000

000018580000000

000018580000000

000018580000000

000023808570000385

000023808570000385

000023808570000385

000023808570000385

00001110000000

00001110000000

00001110000000

00001110000000

00001343800426009136300

0000105180000000

0000105180000000

0000105180000000

0000000009136300

0000000009136300

0000000009136300

000029200000000

000029200000000

000029200000000

000000042600000

000000042600000

000000042600000

364900044590079670868524675836

000018920000000

000018920000000

000018920000000

000018920000000

000018920000000

364900022390079670868524675836

364900022390079670868524675836

364900022390079670868524675836

364900022390079670868524675836

364900022390079670868524675836

00003280000000

00003280000000

00003280000000

00003280000000

00003280000000

00001160000000

00001160000000

00001160000000

00001160000000

00001160000000

00001160000000

91318391261164155861626268240180387076322749609685536358

91318391261164155861626268240180387076322749609685536358

225142302279140546490000000

000030180000000

000030180000000

000030180000000

225142302279140516310000000

225142302279140500000000

08961043000000000

221573980000000000

357436105340500000000

0572695000000000

000016310000000

000016310000000

000044910000000

000044910000000

000044910000000

000044910000000

68804368241136245821117128240180387076322749609685536358

00026100000000

00026100000000

00026100000000

088741892325831130014281138330223170

088741892325831130014281138330223170

0000000119370000

00189232583113002344138330223170

088740000000000

4263125927102086312061020316100484712525462366178

396351826877513235591667000463742087349335635

762218494080000016300

242276176437200000000

0000000042780000

0053720200000000

18585346221876407000015786370226

3386223195976286616670000000

207521000000206005135

00015700000000

0031215000000000

118328672723863580000018600

02150000000000

35183401440000475000

499138925668420000051600

00194000000000

0062937500000000

00266000000000

46015901064928070000025800

17067102350000690000

00021200000000

02320000000000

294119524391247000003270274

433592190777700006450349330

2996765924282764701610020974380543

579109038391400000041200181

73317305731184700007622290175

027945500161000000

52011823911112900000000

40047311453950000020900

109545330213190000000187

0334169222500000000

35195042400000923000

30410763783133200000000

0000000000113030

0000000000113030

00291085360000000

000069760000000

000015600000000

00291000000000

000019210000000

000019210000000

000019210000000

17892000171700004708400

17892000171700004708400

17892000171700004708400

474431017333000000081

47443879333000000081

47443879333000000081

00138000000000

00138000000000

706118599082908090024426923000

706118599082908090024426923000

01530009000224000

624896873225420000278000

8213657336600024426421000

00419000000000

00184000000000

0395424245000000099

0395424245000000099

011200000000099

028342424500000000

000017423767000000

0000023767000000

0000023767000000

00001740000000

00001740000000

752800000000000

752800000000000

752800000000000

009664643100000000

009664643100000000

009664643100000000

009664643100000000

009664643100000000

009664643100000000

0112200025094020906002133500

0000124570000000

0000124570000000

0000124570000000

0000124570000000

0000124570000000

01122000276602090600000

01122000276602090600000

01122000276602090600000

01122000276602090600000

01122000276602090600000

00003620000000

00003620000000

00003620000000

00003620000000

00003620000000

0000950900002133500

0000259000002133500

000025900000000

000025900000000

000025900000000

0000000002133500

0000000002133500

0000000002133500

000069190000000

000067200000000

000067200000000

000067200000000

00001990000000

00001990000000

00001990000000

04631017000000000

04631017000000000

04631017000000000

04631017000000000

04631017000000000

04631017000000000

00005160000000

00005160000000

00005160000000

00005160000000

00005160000000

00005160000000

00003730000000

00002520000000

00002520000000

00002520000000

0000960000000

0000960000000

00001560000000

00001560000000

00001210000000

00001210000000

00001210000000

00001210000000

00001210000000

000028350000000

000022060000000

000022060000000

000022060000000

000022060000000

000022060000000

00006290000000

00006290000000

00006290000000

00006290000000

00006290000000

20556629277659313363000065000

20556629277659313363000065000

205559072521487940000065000

846342914790438700000000

846342914790438700000000

0260811661438700000000

846821859000000000

002270000000000

285827320013060000034000

285827320013060000034000

285827320013060000034000

9241651722431010000031000

9241651722431010000031000

9241651722431010000031000

072225515193630000000

072225515193630000000

0497212751900000000

0289171851900000000

01230000000000

00179000000000

085230000000000

02250000000000

0480000000000

01770000000000

0042403630000000

00276000000000

00003630000000

00148000000000

000082850000000

000082850000000

000069500000000

000069500000000

000069500000000

000069500000000

000013350000000

000013350000000

000013350000000

000013350000000

13302150009158600000

13302150009158600000

13302150009158600000

13302150009158600000

13300000000000

13300000000000

0000009158600000

0000009158600000

00215000000000

00215000000000

515189541567427925495777506434491947357145560988353645473489550154604147

927222621220841552040901249696211334154824919637254078942

0000211530000000

0000211530000000

0000211530000000

0000211530000000

00002550000000

00002550000000

00002550000000

00002550000000

927222621220841552019493249696211334154824919637254078942

04638001500000004641

04638001500000004641

04638001500000004641

06831172790122520000000

06831172790122520000000

0000118360000000

068311727904160000000

00004040000000

00004040000000

00004040000000

0000410000000

0000410000000

0000410000000

0000530000000

0000530000000

0000530000000

927260004805155203366922562111542501963751874301

00001040000000

00001040000000

000503310730000000

000503310730000000

927260004805104872189922562111542501963751874301

927260004805104872189922562111542501963751874301

051520083800048249000

051520083800048249000

051520083800048249000

0000104115744000000

0000104115744000000

0000104115744000000

00001348001799000202200

00001348001799000202200

00001348001799000202200

504749518303405683480192456391466978350934526156305396453852517071595205

235360204413100649257346179749200214135822277103222426268243195560335229

570398200134793167000012075301620

570398200134793167000012075301620

570398200134793167000012075301620

22965719459385236243867176299200214135822277103222426256168165398335229

00006486000108920174290

00006486000108920174290

013930018510676392530226000

013930011400676392530226000

00007110000000

33724001718711232118480100612917200753

0000700000000

000052311848000000

000027640010061000753

337240017187735100029172000

00005240000000

0007851715546000384770041662

00004460000004392

0007851714372000384770017500

000072800000019770

170790079011262014082005169252524115

00079019030371300000

13116000683700000252524115

000068201036900000

396300018400000516900

12136198281538822057112921869250300434601279918551

12136198281538822057112921869250300434601279918551

51267593004291000001220301

000035270000000

5126759300764000001220301

35950481981456031284210609953406263046433546631061530849846

35673393791456025128174809953219371367633546631061530842872

2778819061563580018689167880006974

01167300331713920001394002658314307

01167300331713920001394002658314307

15697211112553528127113163960701971023483021594

15697211112553528127113163960701971023483021594

1031004514129753389015655084552321661792039639910486854506120198

4470991220858269793167310958574197319845076803158698

5839136019297533804329571528792120812178423201597924647561500

75839656030841846610834901461510138221230163902

000308452040000005818

01930700606510828021299570138221230118223

01781900492600000027126

7582530002271068801619400012735

60870023920363021534678100000

60870016139264510646678100000

000778198510888000000

001541302830000000

001541302830000000

001541302830000000

00009450000003744

00009450000003744

00003060000000

00003060000000

00006390000003744

00006390000003744

11547709600204930000020940

11547709600204930000020940

000025020000000

000025020000000

000016300000000

000012830000000

00003470000000

00002360000000

00002360000000

000086920000000

000086920000000

065340011590000000

06534002530000000

00009060000000

000020630000000

00003010000000

000017620000000

1046500000000000

1046500000000000

0562001070000000

0562001070000000

000018430000000

000018430000000

108200000000000

108200000000000

000022610000020940

00001830000020940

000020780000000

60481241110858758629571273900844301961800

2775968492220365313006068890687300

2775426792220122313006068890000

009222000000000

206239260053713006068890000

00006600000000

71334100260000000

00005770000687300

00001010000000

00004760000687300

000011980000000

000011980000000

05417003940000000

05417003940000000

00002610000000

00002610000000

00004500000000

00004500000000

00004500000000

00002670000000

00002670000000

00002670000000

197948016361603104661480015540182900

14760004338007280182900

00000007280000

00002860000000

000000000182900

000036620000000

14760002480000000

00001420000000

000015170000000

00004960000000

00006770000000

00003440000000

00002970000000

00002970000000

4190126314691746148008260000

000001480000000

000010810000000

419012631469665008260000

00001680000000

00001680000000

8448037313424000000000

000012810000000

8448037313400000000

00004100000000

00007090000000

0005983940129040001091600

00005460000000

00005460000000

00059832690000000

00059832690000000

0000125129040001091600

00001250000000

0000012904000000

0000000001091600

00003320000000

00003320000000

00003320000000

1294224700134630000000

0000112420000000

0000112420000000

00002760000000

00002760000000

129400018900000000

129400018900000000

0224700550000000

0224700550000000

00004290000000

00004290000000

00004290000000

00004290000000

68455544027638445600000942410604

68455544027638445600000942410604

68455544027638445600000942410604

00001370000000

0000760000000

00001300000000

00003830000000

0144400990000000

4863410002763854900000942410604

19820001150000000

00009750000000

00004820000000

000015100000000

1656129147012275238484447117986186120114311044022524

5002256840727983371010580900019601

01800014530058090000

000014530058090000

01800000000000

022807200000000

022807200000000

500225276006530371010000019601

500225276004809371010000019601

000017210000000

0000410000002923

0000410000002923

0000410000002923

1155934630122031582473701798612803011431104400

00006140000000

00006140000000

00005360000000

00005360000000

030510015287370000000

000007370000000

00004870000000

030510010410000000

00005000000000

00005000000000

115594120122031032001798612803011431104400

00002560000000

1155941201220367940179860011431104400

000017040000000

00002810000000

00002610000000

000000063410000

00008210000000

00002030000000

000000064620000

000023260000000

000023260000000

22283525185427072617105519228918569319712622199882970132321287206223104

1850001315076648446000581

18500000000000

18500000000000

000075000000581

000075000000581

000000084460000

000000084460000

00002780000000

00002780000000

00009620000000

00009620000000

000000766400000

000000766400000

5582396004155732814415601495325621608098472742

000000005621000

000000005621000

00001620000000

00001620000000

632396000203614415601458220202883462742

632396000203614415601458220202883462742

205300000000000

205300000000000

289700415551300037100405215010

000034340000000

289700415515460037100405215010

00001500000000

0002478945900000011474

0002478945900000011474

0001926000000000

00003730000000

0005529000000011474

0000860000000

197158310996361606007507800

00001150000000

00001150000000

458921480000750000

01020000000000

453341480000750000

04560000000000

152691951430149100007800

90269225000000000

62201348215108900007800

00004020000000

022137821500000000

00020600000000

00020600000000

000011470000000

00007100000000

00007100000000

00004370000000

00004370000000

00007400000000

00007400000000

00007400000000

337441640051690000004897

337441640051690000004897

000035510000000

337441640016180000004897

000042810000000

000042810000000

000015970000000

000014860000000

000011980000000

21349724214726962714147517024417127818344820394577349126163277359203410

0000295030233000537100

0000114230233000537100

000018080000000

0787281000000000

0787281000000000

0018593000000000

0018593000000000

21349724136025075314147516729414104518344820394577349120792277359203410

0107430013500000004638

000091500002999100

00003740000000

1774302108512147681209101361071109481554131787687734990230237482149673

2096819766020565142173009702517700999443931

000063302803500000

00006080000000

00000000031005168

00002260000000

000047770000000

00006010000000

15099035985074860000261298830

555378382345042924611921000022239123470

555378382345042924611921000022239123470

000011580000000

000011580000000

555378382345042923073921000022239123470

555378382345042923073921000022239123470

00003800000000

00003800000000

12564315865151000640000

12564315865151000640000

00003540000000

00003540000000

00003540000000

000011560000000

000011560000000

000011560000000

12564315865000640000

842550000000000

842550000000000

4138815865000640000

4138815865000640000

000032740000076760

000032740000076760

000032740000076760

000032740000076760

000014500000076760

000018240000000

104300043580013530000

104300043580013530000

104300036100013530000

000031830000000

000031830000000

00004270000000

00004270000000

000000013530000

000000013530000

104300000000000

104300000000000

00007480000000

00007480000000

00007480000000

0004142302000001473600

0004142302000001473600

0004142302000001473600

00002740000000

00002740000000

00002740000000

0004142274600001473600

0004142274600001473600

0004142274600001473600

251538370040651226573240910862738082387

251538370040651226573240910862738082387

115638370036131226573223980862738082387

00002260000000

00002260000000

00002260000000

2220002328200000346

2220002328200000346

2220002328200000346

9343837001232944573223980862738082041

9343837001176944573223980862738082041

9343837001176944573223980862738082041

0000560000000

0000560000000

000021320000000

000019760000000

000019760000000

00001560000000

00001560000000

13590004520016930000

13590004520016930000

13590004520016930000

13590004520016930000

338160352126328539395125348704454718455694329759394381265826370362380889

95613062519508137908843750684574184352252704241146571937601

00005980000556100

00005980000556100

00005980000556100

00005980000556100

0000149211532000000

0000011532000000

0000011532000000

0000011532000000

000014920000000

00001780000000

00001780000000

000013140000000

000013140000000

27092748290430867002288701481001753226726

27092748290430867002288701481001753226726

00003140000000

00003140000000

27092748200570601035701481001753226726

00002090000000

03844001224010357014810092426282

00009100000003293

27092363800297000000829012657

00002010000000

000000000004494

00001920000000

000011060000000

000011060000000

00904304110000000

00904304110000000

00004880000000

00004880000000

00002600000000

00002600000000

000026601253000000

0000001253000000

00002660000000

00001190000000

00001190000000

5557286001001760634367770133953789411481481870

5557286001001760634367770133953789411481481870

00000000047700

00000000047700

000009724000000

000009724000000

5557286001001760423270530133953789411004481870

00000139660037894000

000000034060000

0000872000009550

00003600000000

00003370000000

00001210000000

00005614700000444000

0286001001700000000

0000013087000000

000013640000000

0000122200998901100428320

555700000000000

0000900000000

0000900000000

00001210000000

00001210000000

0000970033700000

0000970033700000

0000970033700000

0000970033700000

1295283104653773169462375345312675707072010875

000033292375190671593707072010875

000033292375190671593707072010875

000033292375190671593707072010875

322003773166800108200000

0000650000000

0000650000000

32200377346100108200000

000043500108200000

32200000000000

000377300000000

0000260000000

000011420000000

00004210000000

00001300000000

00005910000000

973010465011620000000

97300000000000

97300000000000

0010465000000000

0010465000000000

00007500000000

0000610000000

00003470000000

0000580000000

00002840000000

00004120000000

00004120000000

000057960000000

00001090000000

00001090000000

000056870000000

000051030000000

00005840000000

0000950000000

0000950000000

0000950000000

028300379301546400000

00002090000000

00002090000000

00002440000000

00002440000000

0000001546400000

0000001546400000

0000360000000

0000360000000

02830000000000

02830000000000

0000980000000

0000980000000

0000900000000

0000900000000

00003170000000

00003170000000

000027990000000

000027990000000

00004410000000

00004410000000

00004410000000

00006620000000

0000460000000

0000460000000

00006160000000

00003950000000

00002210000000

00004550000000

00004550000000

00004550000000

00004550000000

00004550000000

328599321501309031381335259812404034398276286237341677241712304643343288

00002250000000

00002250000000

00002250000000

00002250000000

180990224737193029744000000

516000000000000

516000000000000

516000000000000

0022473719300000000

0022473719300000000

0022473719300000000

0000029744000000

0000029744000000

0000029744000000

1293900000000000

1293900000000000

1293900000000000

28032618898048198336197208001200613307898180878174080181446195307294507

4164546506018282815043322860399105181701203296540

4164546506018282815043322860399105181701203296540

4164546506018282815043322860399105181701203296540

2133371046344819815336915652416763320628669261122263179245183275185722

00003900000000

00003900000000

174175839033947113344313717112584913156151124115826148252159627152437

2630008230027910000

13745259170117639741410177612060312434226559115826117491129042113014

00001180000000

000013540721900626683000

000012380000000

35570003340000000

8401180940202581163787018090001409926684

16321001577141485159036840718000

0000610000000

000000000007149

0017714000000000

000012890000000

45090003430000000

05308002940000000

00004720000000

000011430000641300

00002830000000

00002860000052950

000026640000000

000019390000000

00007570000000

013310000000000

00002980000000

00003390000000

3672099940287300001090200

00004330000000

00004860000000

00008490000000

00003070000000

00004130000028915590

00002140000000

0000970000000

0000970000000

00007810000000

00007810000000

00004220000004401

00004220000004401

3916220731872719926176634178474725181376437309932364828884

00002049000643744400

3542499909374220600745301288405030

36141018587274119584133423320078003011954011625

242351180643340288361186722681057113691574

295834290035390240460002327910655

3105105010013498694240000220104729

310500034680240000220104729

310500000000000

000000000004729

000024240000220100

000000240000000

00007880000000

00002560000000

0105010071070000000

0105010062600000000

00002940000000

00005530000000

000011960000000

000011960000000

00001727694000000

00001727694000000

00001360000000

00001360000000

00001360000000

22239273390022800099212717070007516

12089000108650000007516

000000000007516

12089000108650000000

00003120000000

00003120000000

10150273390011623099212717070000

0000000316780000

101502733900528309921200000

000063400000000

0000000400290000

83210007800000011667

83210007800000011667

0000780000000

0000780000000

8321000000000011667

8321000000000011667

000023720000048320

000023720000048320

00001350000000

00001350000000

000013570000000

000013570000000

00008800000048320

00004250000000

00004550000048320

1832111649723836037945320701006053303510454916545446020826500

017797000000000

017797000000000

06897000000000

01090000000000

00001060000032900

00001060000032900

00001060000032900

0000334300001733500

000052900001733500

000032300001733500

00002060000000

00005700000000

00005700000000

000012020000000

000012020000000

00004840000000

00004840000000

00005580000000

00005580000000

0000255014800000

0000255014800000

0000255014800000

6881503352997173000000000

2961427852047173000000000

004708173000000000

01361747339000000000

2966610000000000

92197291000000000

92197291000000000

300558659000000000

300558659000000000

68756330394064303357724912258884213405217480

00001520000000

00001520000000

000000000017480

000000000017480

00002590004213000

00002590004213000

00006370000000

00006370000000

00006990000000

0000650000000

00002080000000

00004260000000

6875000826024912220340000

6875000826024912220340000

00004380000000

00004380000000

000000000405200

000000000405200

0242394000000000

0242394000000000

000024190000000

000024190000000

060880080533577038540000

060880080533577038540000

00001950000000

00001950000000

000013990000000

00002890000000

00002890000000

000011100000000

000011100000000

107589495718487236215205376702879757866116124124633776120

000000797500000

000000797500000

000011140000000

000011140000000

66370000000006380

00000000006380

663700000000000

41219495718487236215194236702807866116124124633719700

41219495718487236215194236702807866116124124633719700

000000000050040

000000000050040

353216024001303854350391000000

000016360000000

000016360000000

000016360000000

353216024001110754350391000000

000029650000000

000020600000000

00009050000000

30581602400456854350391000000

30581602400456854350391000000

47400000000000

47400000000000

00002910000000

00002910000000

000026080000000

000024520000000

00001560000000

00006750000000

00006750000000

00002950000000

00002950000000

00002950000000

0000374618722534338102143142462185437114

0000374618722534338102143142462185437114

0000760000000

0000760000000

000001858353363810214314040105036572

000001858353363810214314040105036572

000019440000000

00003240000000

000015460000000

0000740000000

00001726139700020620804542

00001726139700020620804542

00002820000000

00002820000000

00002820000000

00002820000000

010740147754515263108458948323190046550

010740147754515263108458948323190046550

0014772251174300272380000

0014772251174300272380000

000011990000000

000011990000000

00613000000000

00613000000000

00337000000000

00337000000000

0000000272380000

0000000272380000

0000105440000000

0000105440000000

0052722500000000

0052722500000000

000029290000000

000029290000000

000029290000000

000029290000000

010740004240894850810000

0107400000894800000

0107400000894800000

000000894800000

0107400000000000

00004240050810000

00004240050810000

00004240050810000

00032016710845000046550

00032016710845000046550

00032016710845000046550

000000000046550

00032016710845000000

0000532200002765000

0000532200002765000

0000532200002765000

0000532200002765000

0000532200002765000

0000532200002765000

0000532200002765000

326510927006664042480219100522100

000000000522100

000000000522100

000000000522100

000000000522100

000000000522100

000000000522100

326510927006664042480219100000

326510927005571042480219100000

326510927005571042480219100000

326510927005571042480219100000

326510927005571042480219100000

326510927005571042480219100000

000010930000000

000010930000000

000010930000000

000010930000000

000010400000000

0000530000000
