# Supplementary figures and images for: Integrated analysis of the aqueous humor microbiome and lens capsule transcriptome in high myopia cataract: a pilot study (part 2 of 2)
Source: Front Med (Lausanne). 2026 Jun 16;13:1845205. doi: 10.3389/fmed.2026.1845205 (PMC13314463; doi:10.3389/fmed.2026.1845205)

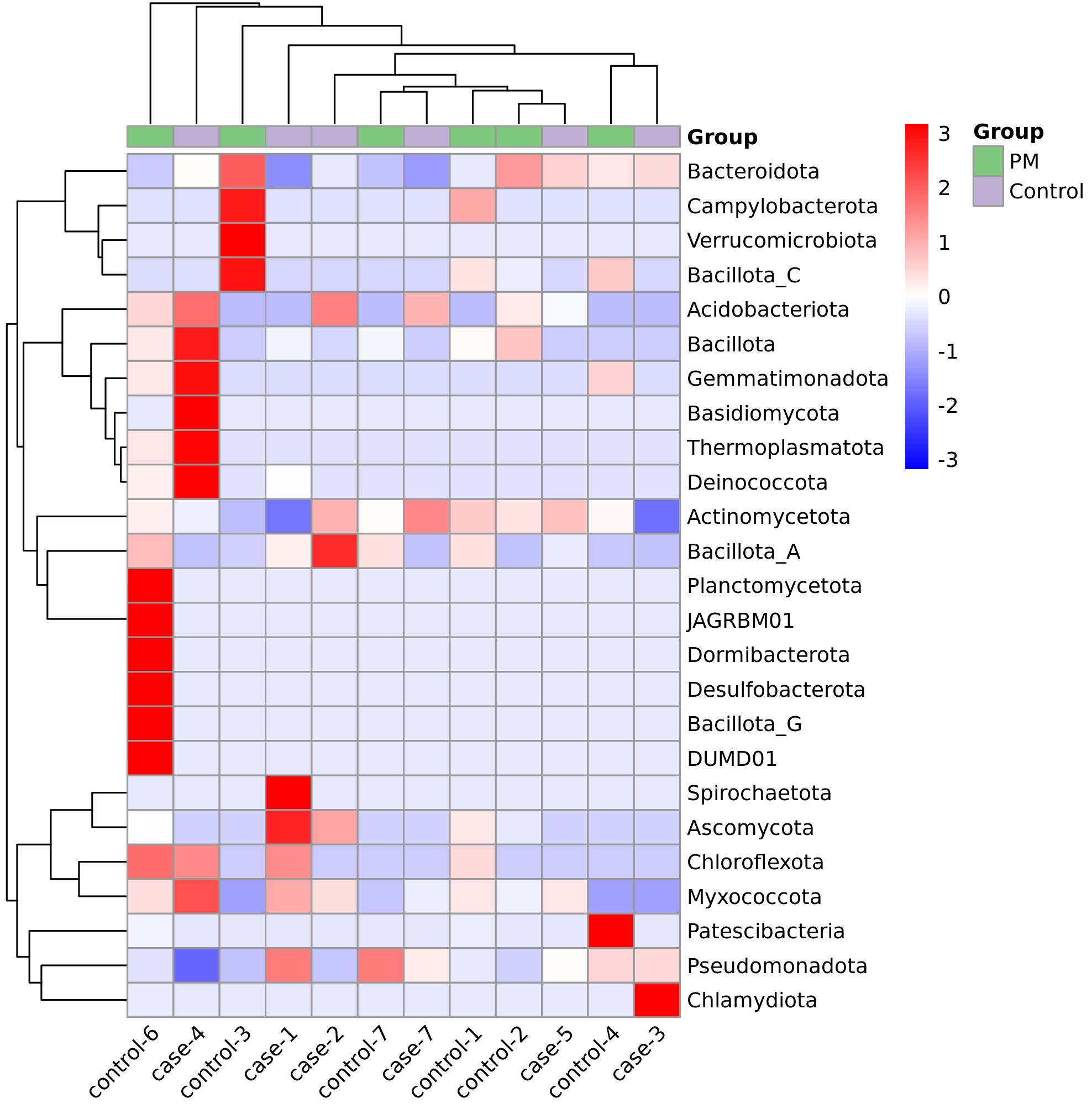

Supplement: Supplementary file 3 [file Data_Sheet_1.zip › 1.Community_Structure/heatmap/C372089/Phylum_top30_cluster.png]

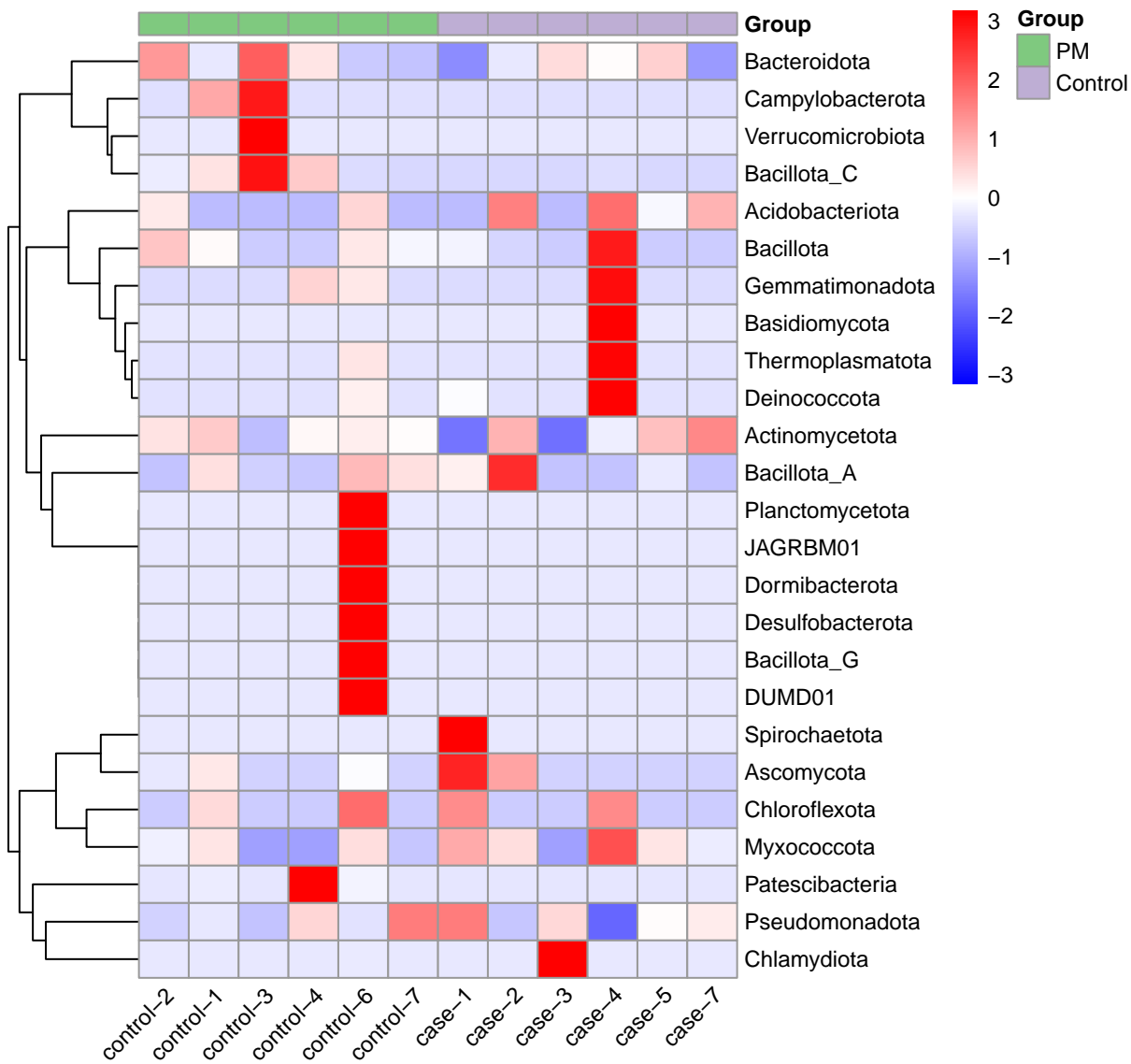

Supplement: Supplementary file 3 [file Data_Sheet_1.zip › 1.Community_Structure/heatmap/C372089/Phylum_top30_nocluster.pdf]

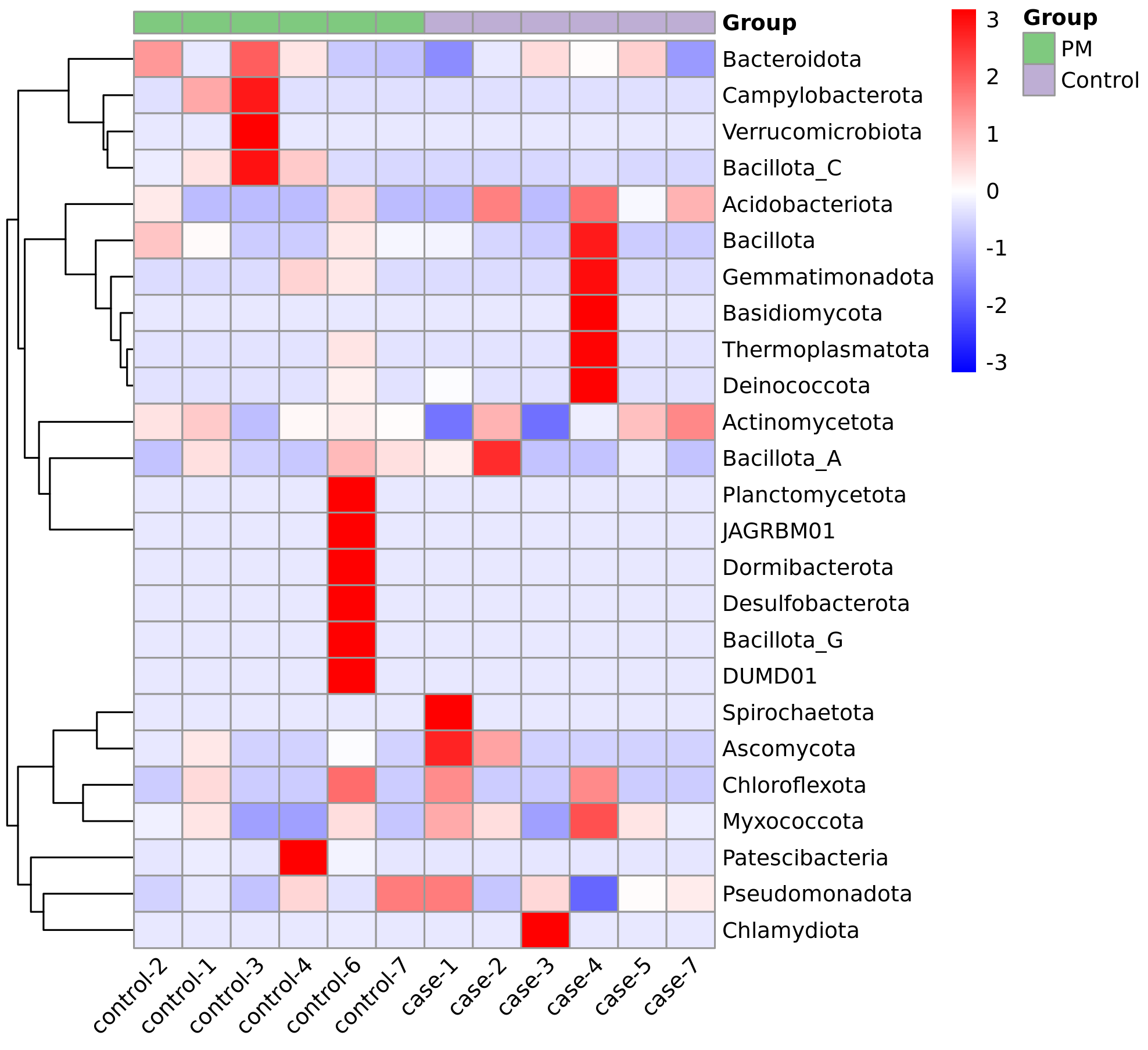

Supplement: Supplementary file 3 [file Data_Sheet_1.zip › 1.Community_Structure/heatmap/C372089/Phylum_top30_nocluster.png]

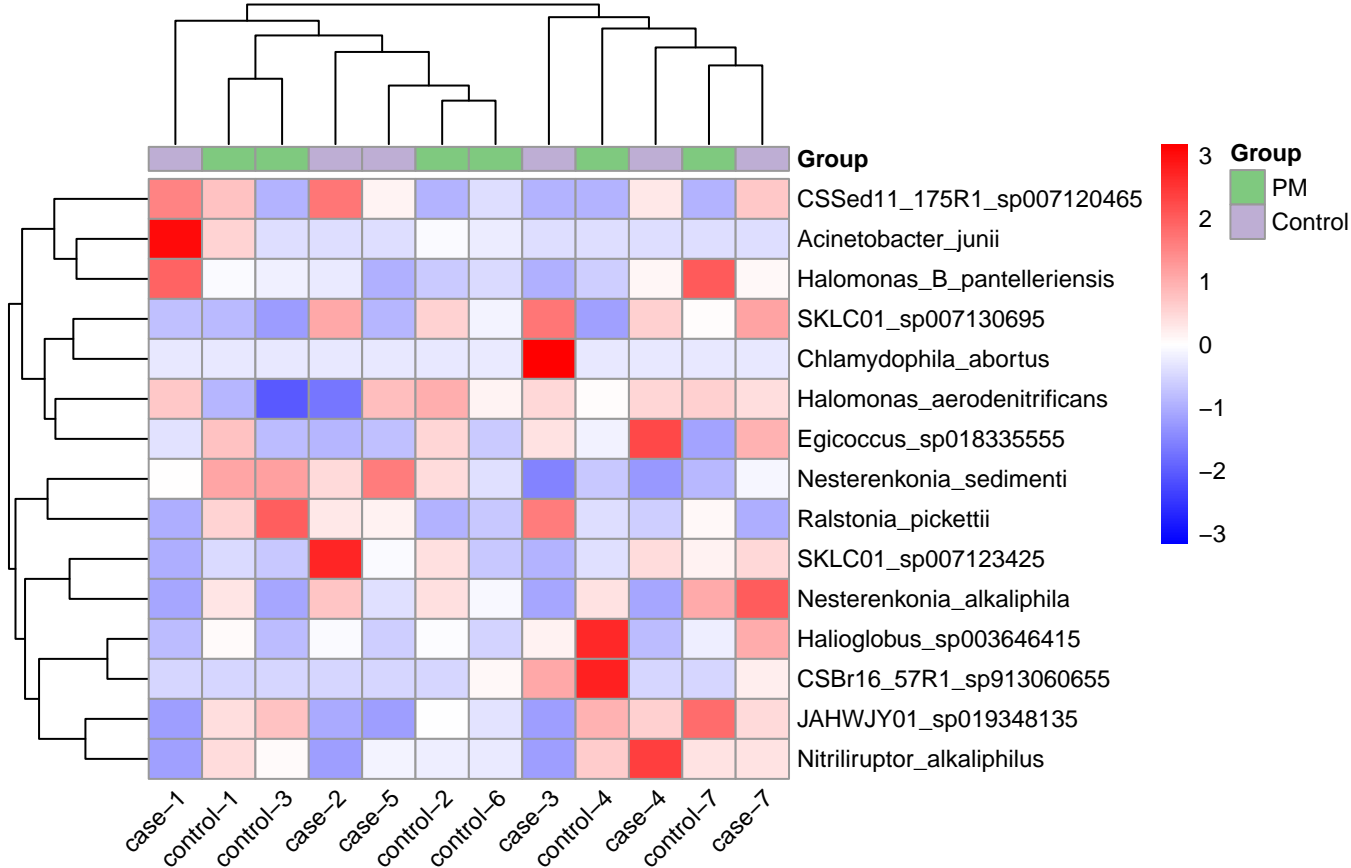

Supplement: Supplementary file 3 [file Data_Sheet_1.zip › 1.Community_Structure/heatmap/C372089/Species_top15_cluster.pdf]

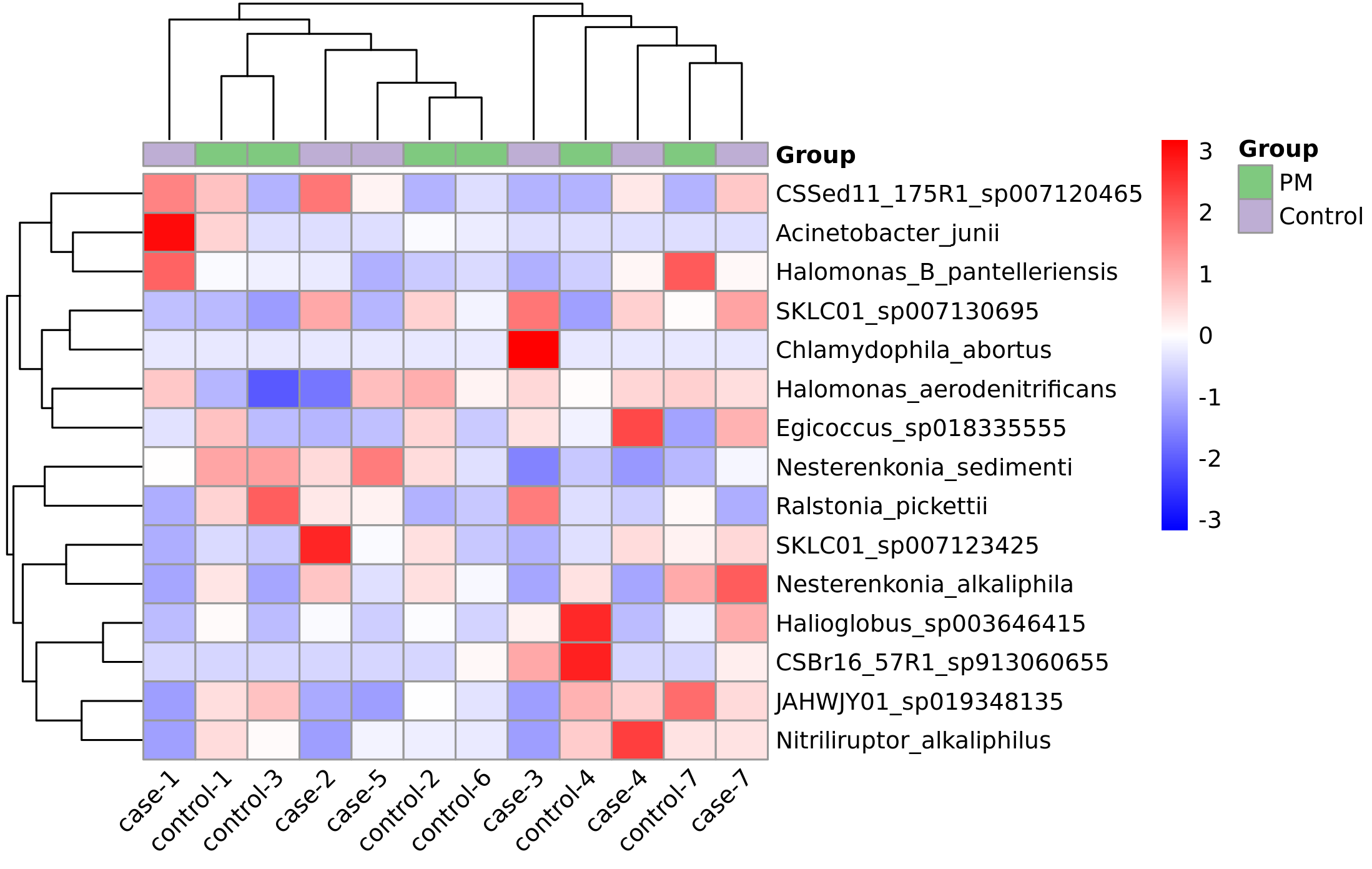

Supplement: Supplementary file 3 [file Data_Sheet_1.zip › 1.Community_Structure/heatmap/C372089/Species_top15_cluster.png]

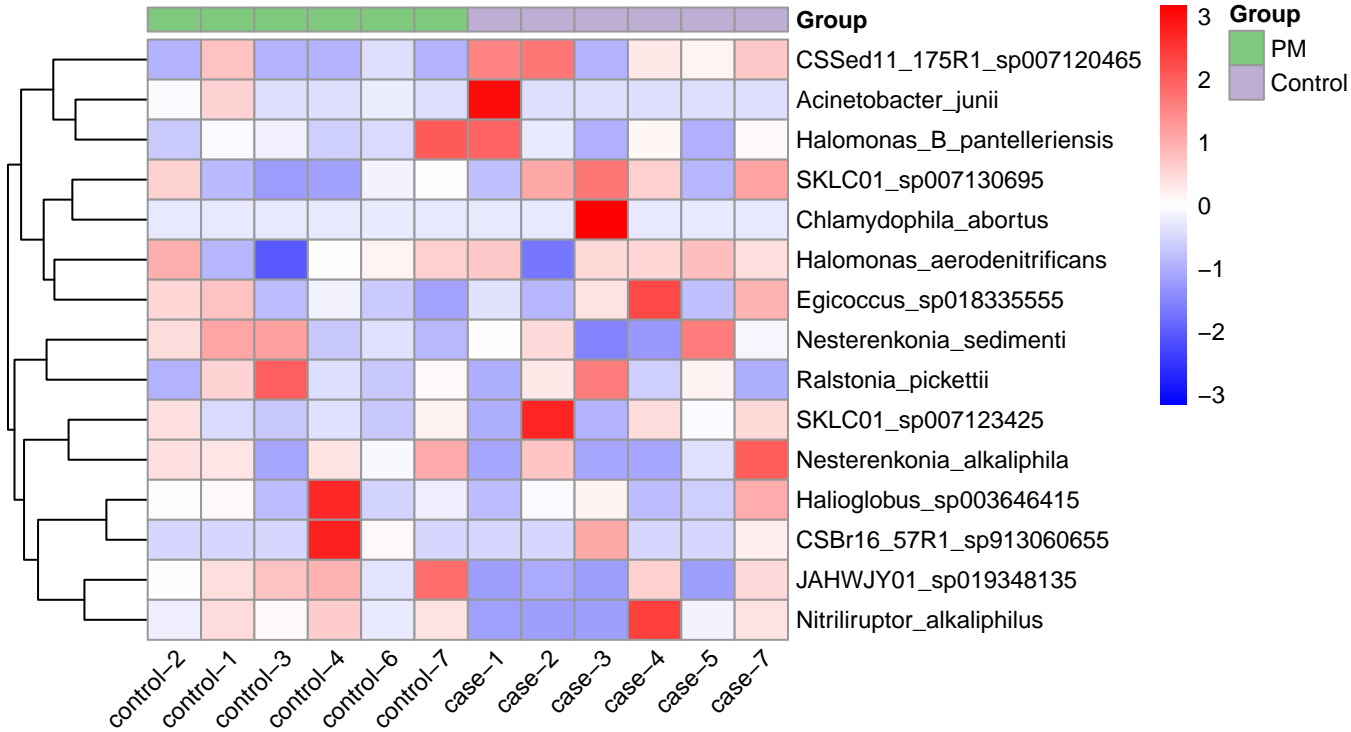

Supplement: Supplementary file 3 [file Data_Sheet_1.zip › 1.Community_Structure/heatmap/C372089/Species_top15_nocluster.pdf]

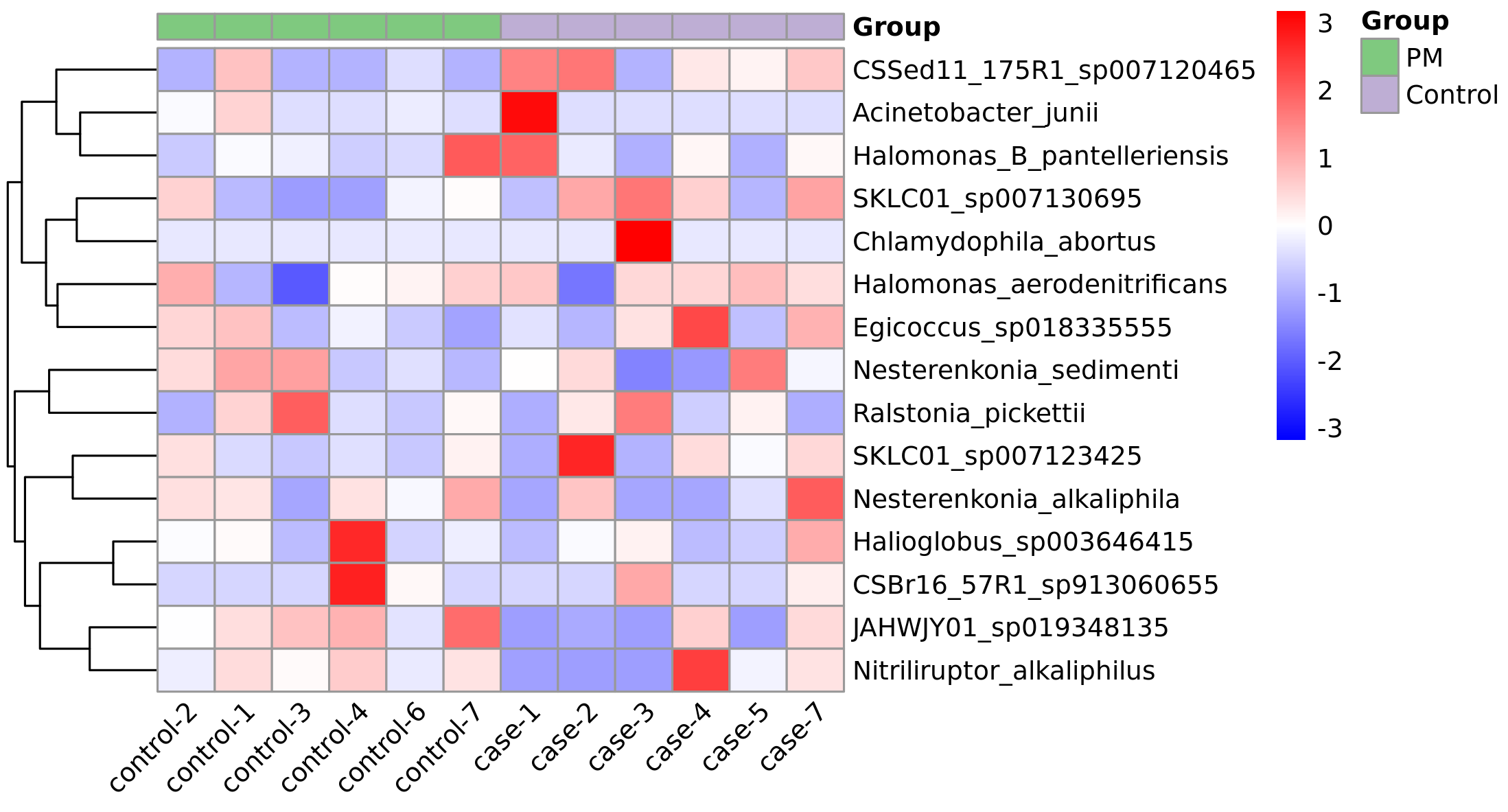

Supplement: Supplementary file 3 [file Data_Sheet_1.zip › 1.Community_Structure/heatmap/C372089/Species_top15_nocluster.png]

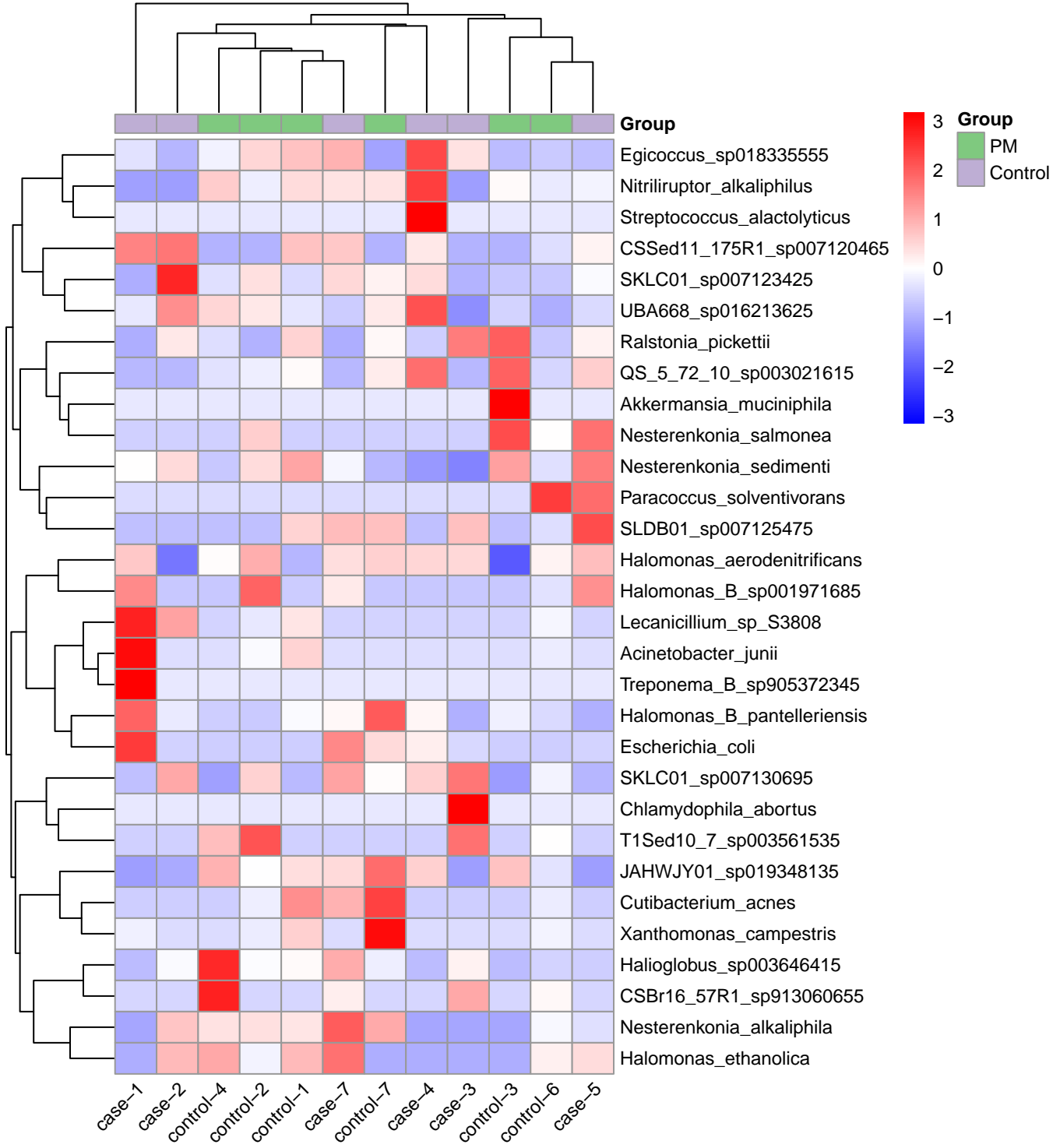

Supplement: Supplementary file 3 [file Data_Sheet_1.zip › 1.Community_Structure/heatmap/C372089/Species_top30_cluster.pdf]

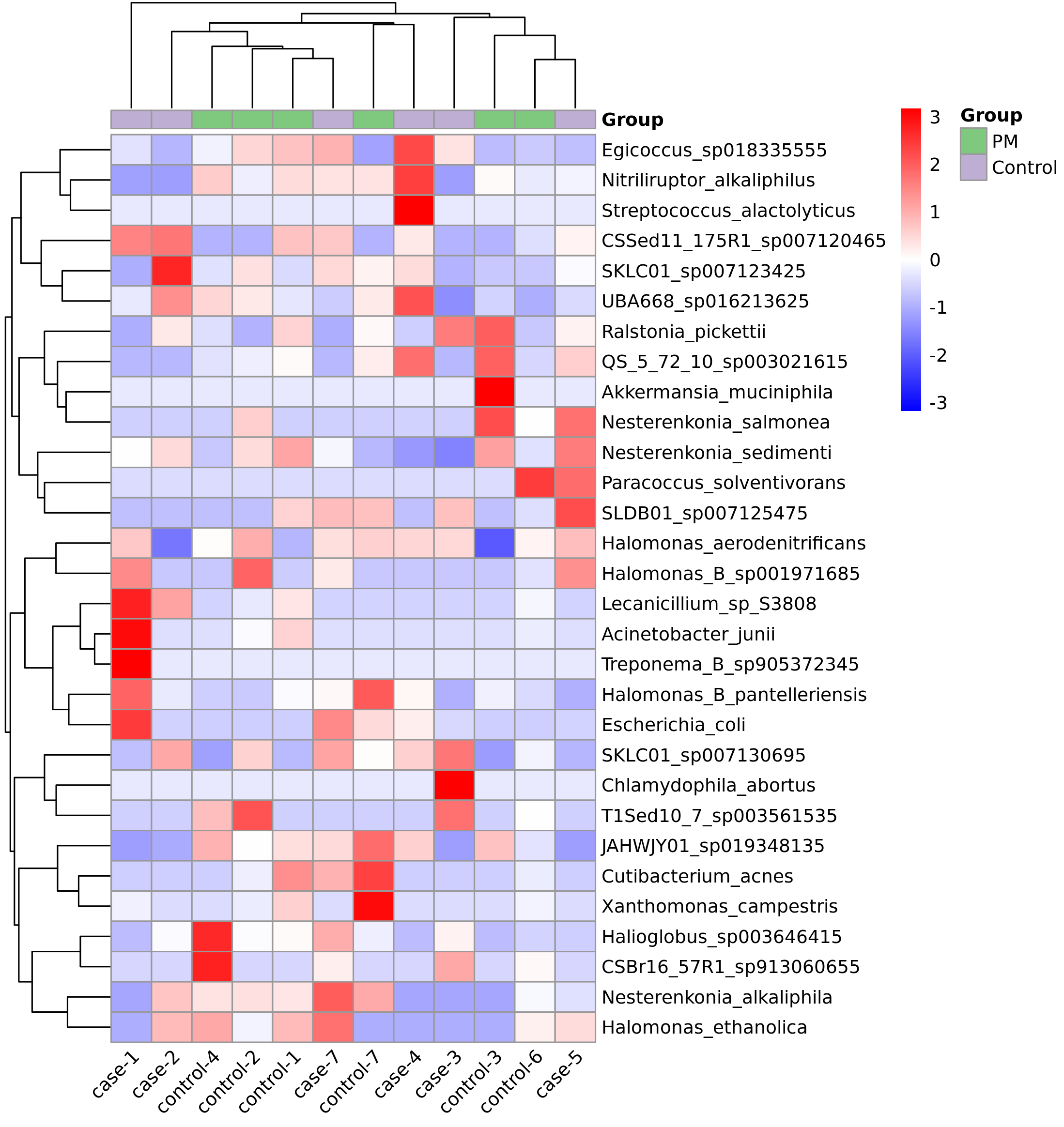

Supplement: Supplementary file 3 [file Data_Sheet_1.zip › 1.Community_Structure/heatmap/C372089/Species_top30_cluster.png]

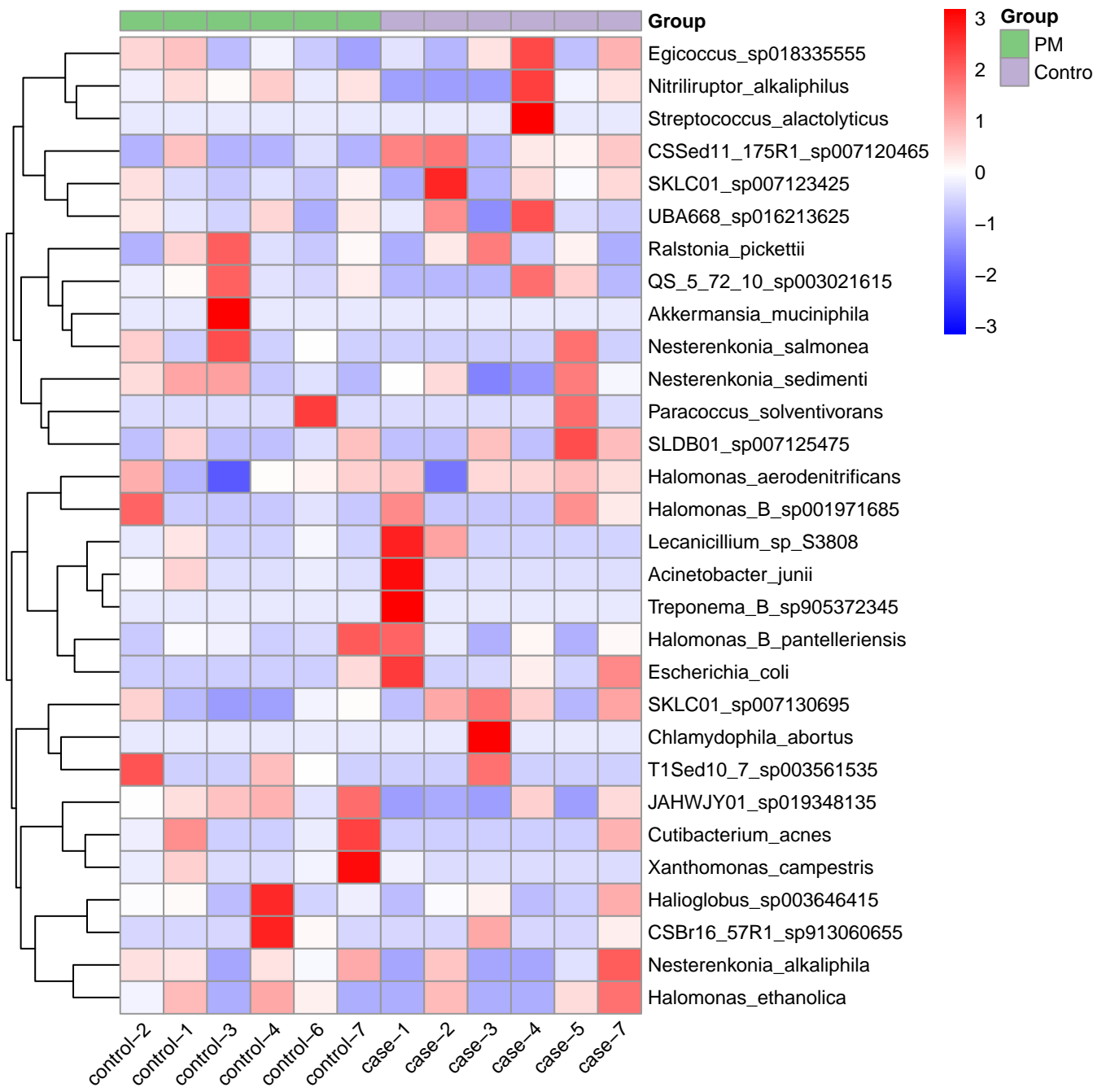

Supplement: Supplementary file 3 [file Data_Sheet_1.zip › 1.Community_Structure/heatmap/C372089/Species_top30_nocluster.pdf]

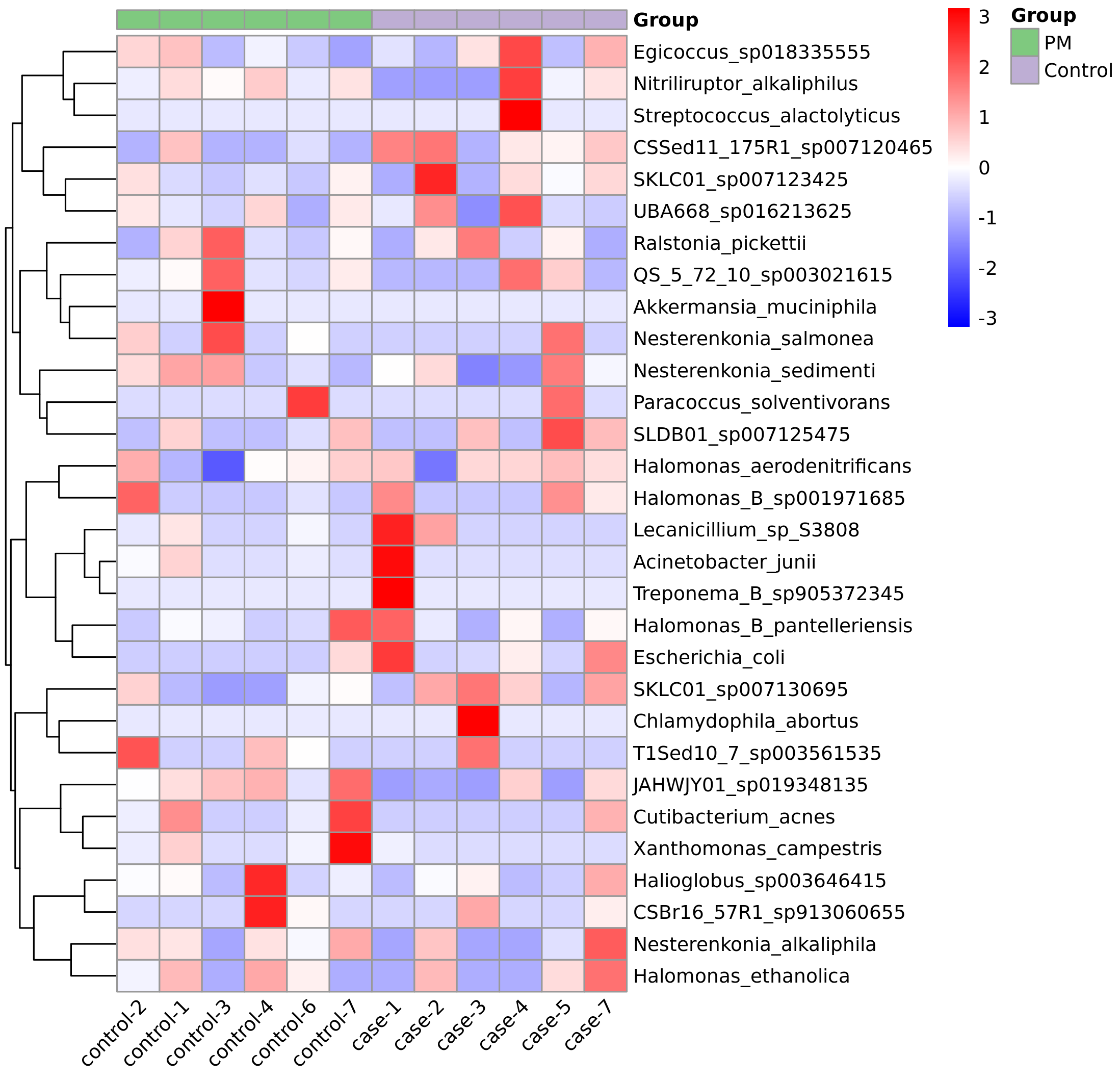

Supplement: Supplementary file 3 [file Data_Sheet_1.zip › 1.Community_Structure/heatmap/C372089/Species_top30_nocluster.png]

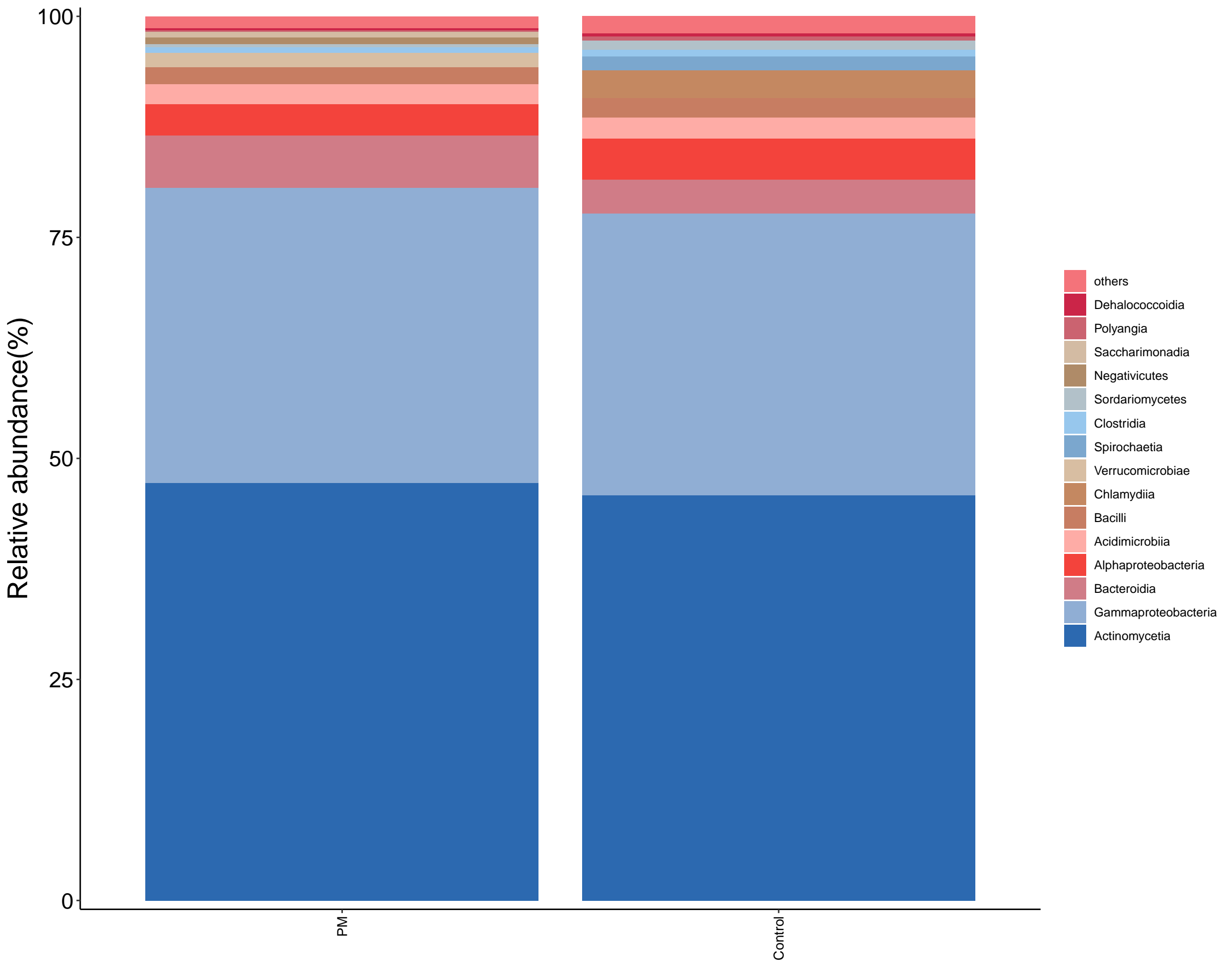

Supplement: Supplementary file 3 [file Data_Sheet_1.zip › 7.Average/1.Community_Structure/barplot/C372089/Class_top15_others.pdf]

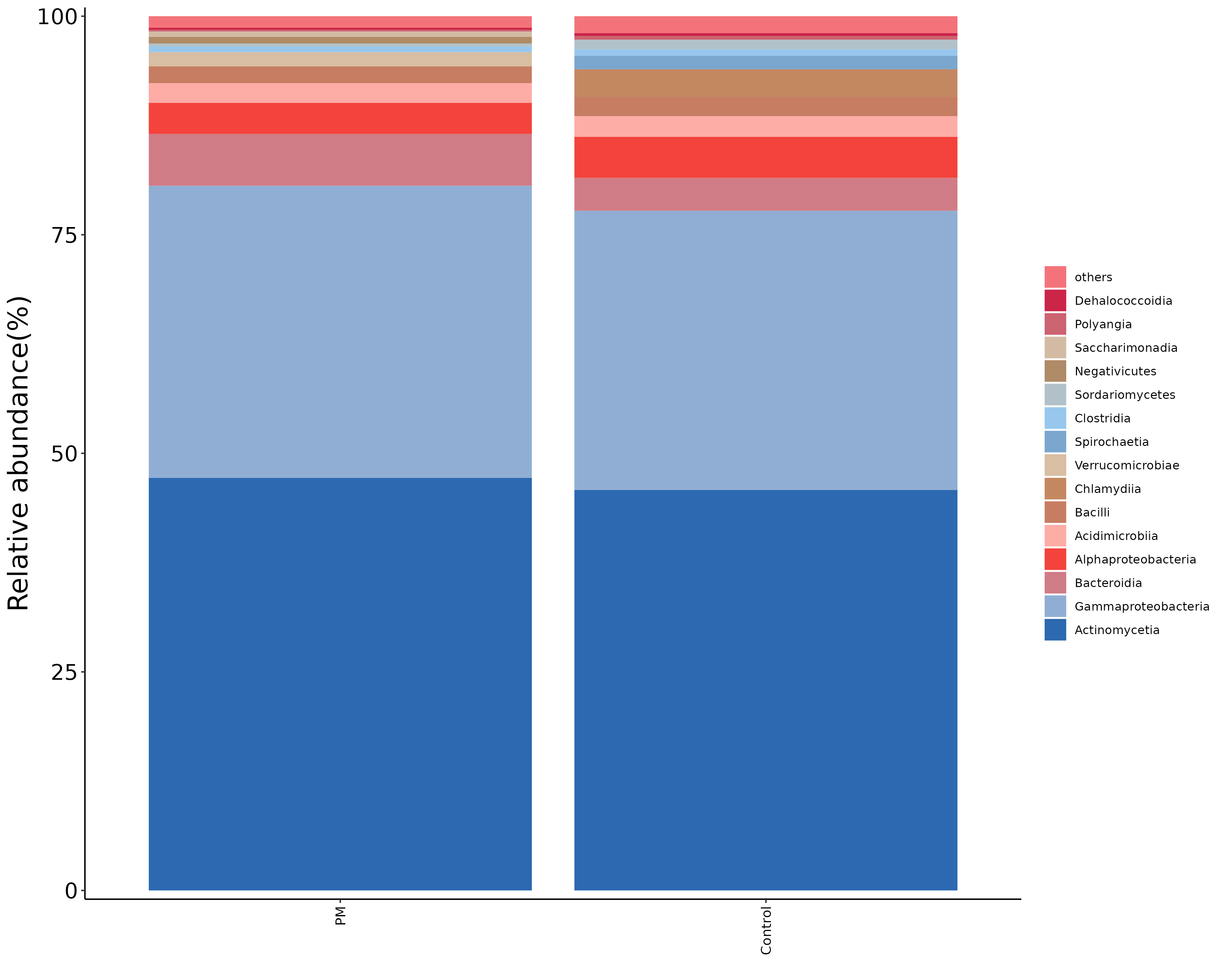

Supplement: Supplementary file 3 [file Data_Sheet_1.zip › 7.Average/1.Community_Structure/barplot/C372089/Class_top15_others.png]

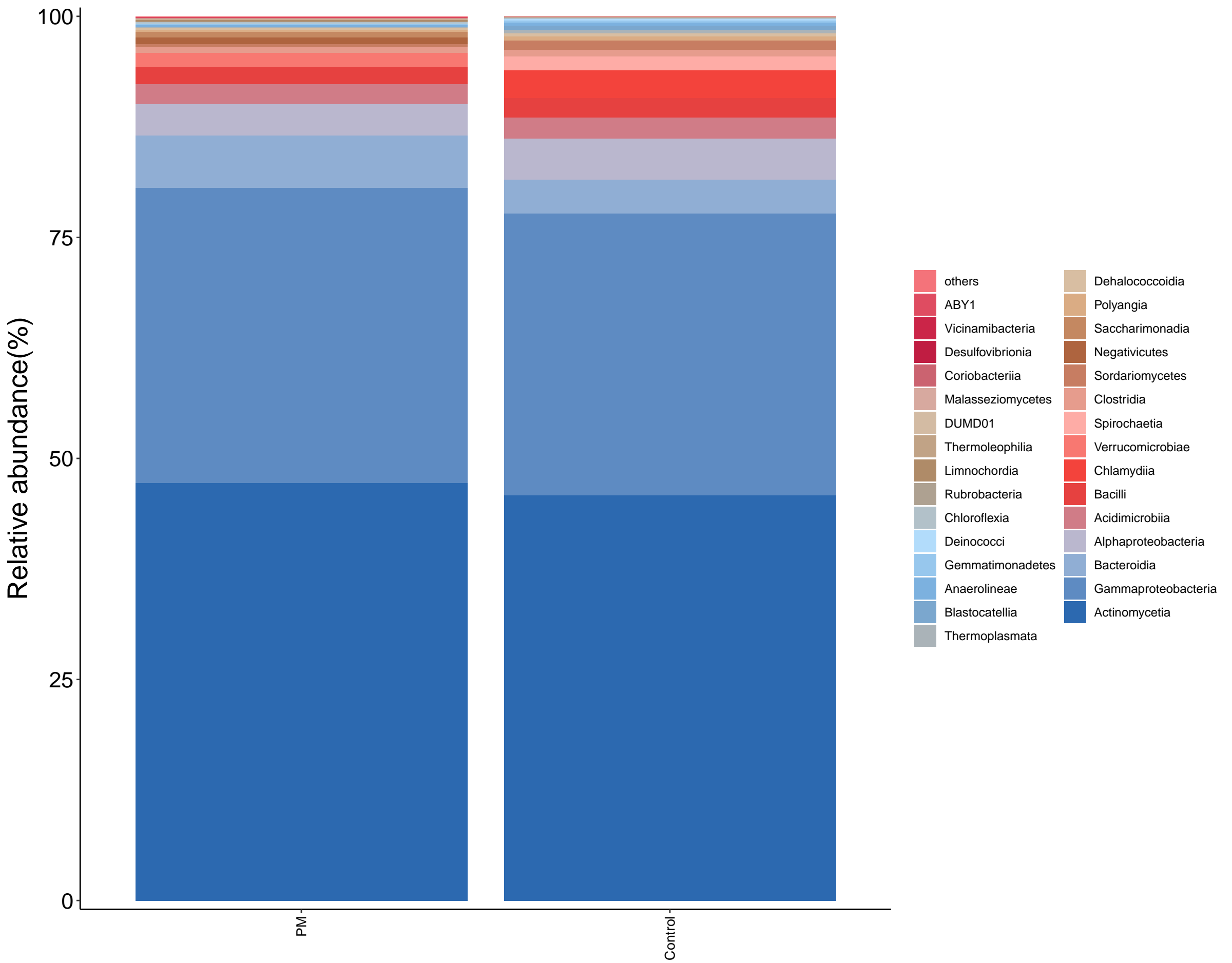

Supplement: Supplementary file 3 [file Data_Sheet_1.zip › 7.Average/1.Community_Structure/barplot/C372089/Class_top30_others.pdf]

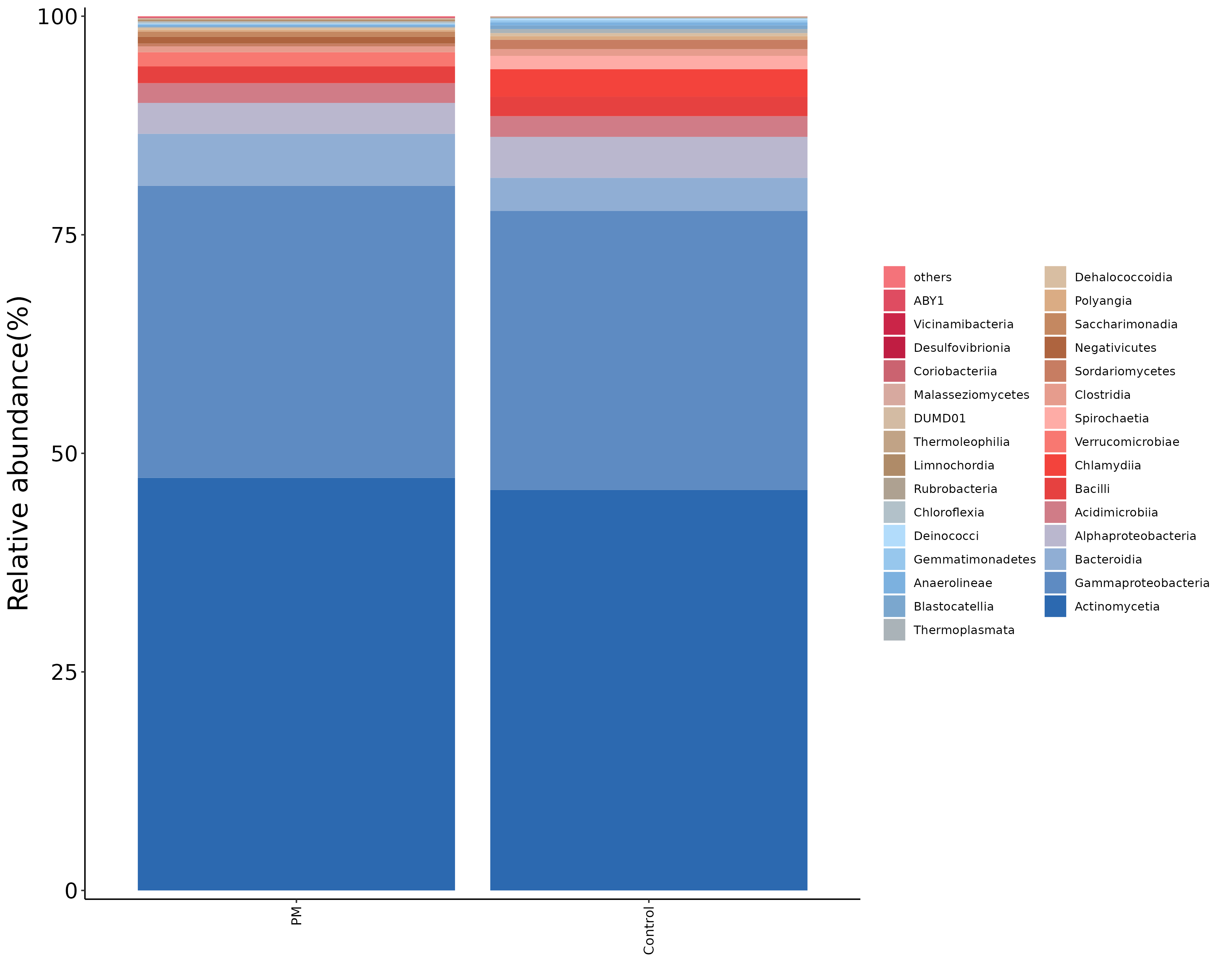

Supplement: Supplementary file 3 [file Data_Sheet_1.zip › 7.Average/1.Community_Structure/barplot/C372089/Class_top30_others.png]

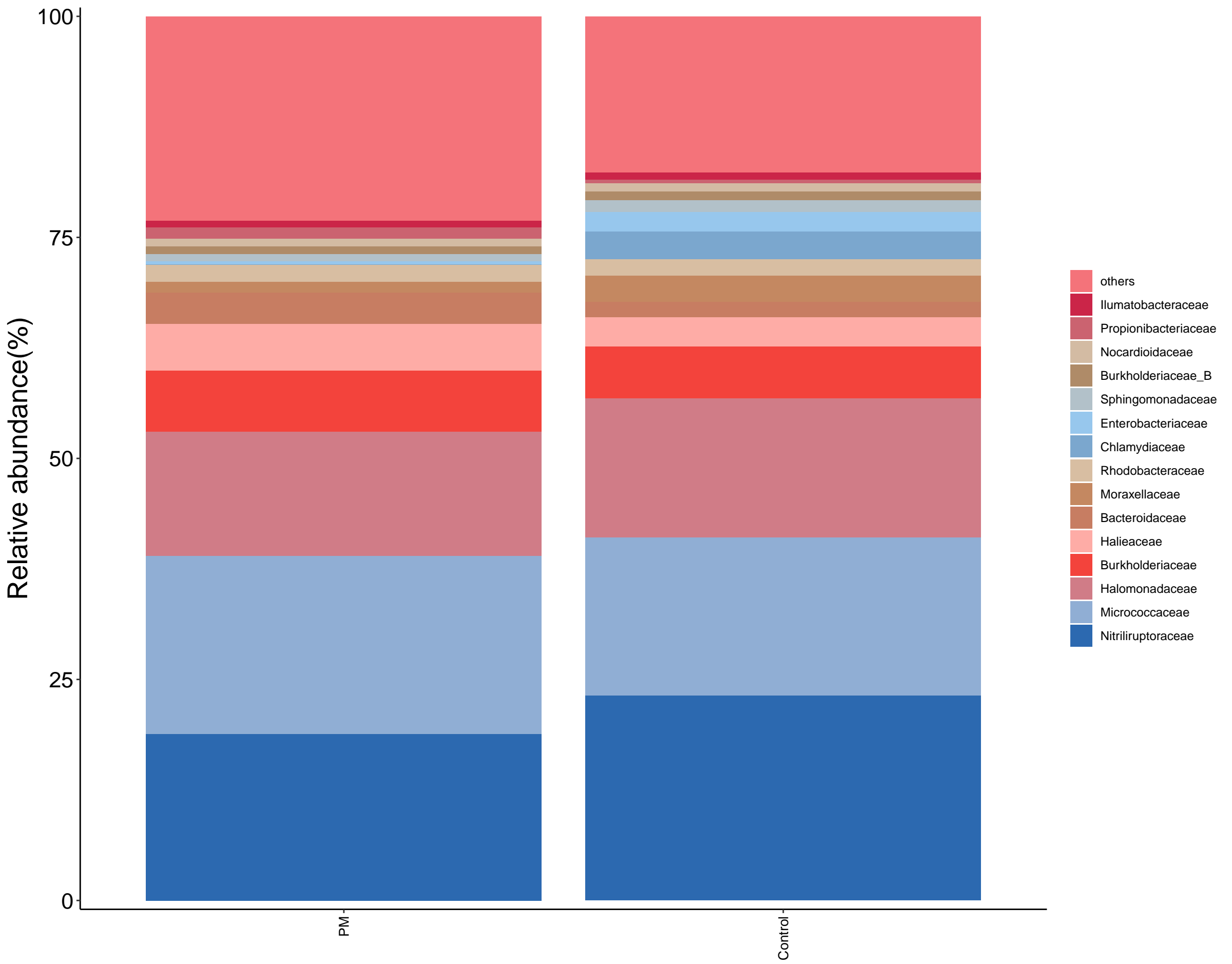

Supplement: Supplementary file 3 [file Data_Sheet_1.zip › 7.Average/1.Community_Structure/barplot/C372089/Family_top15_others.pdf]

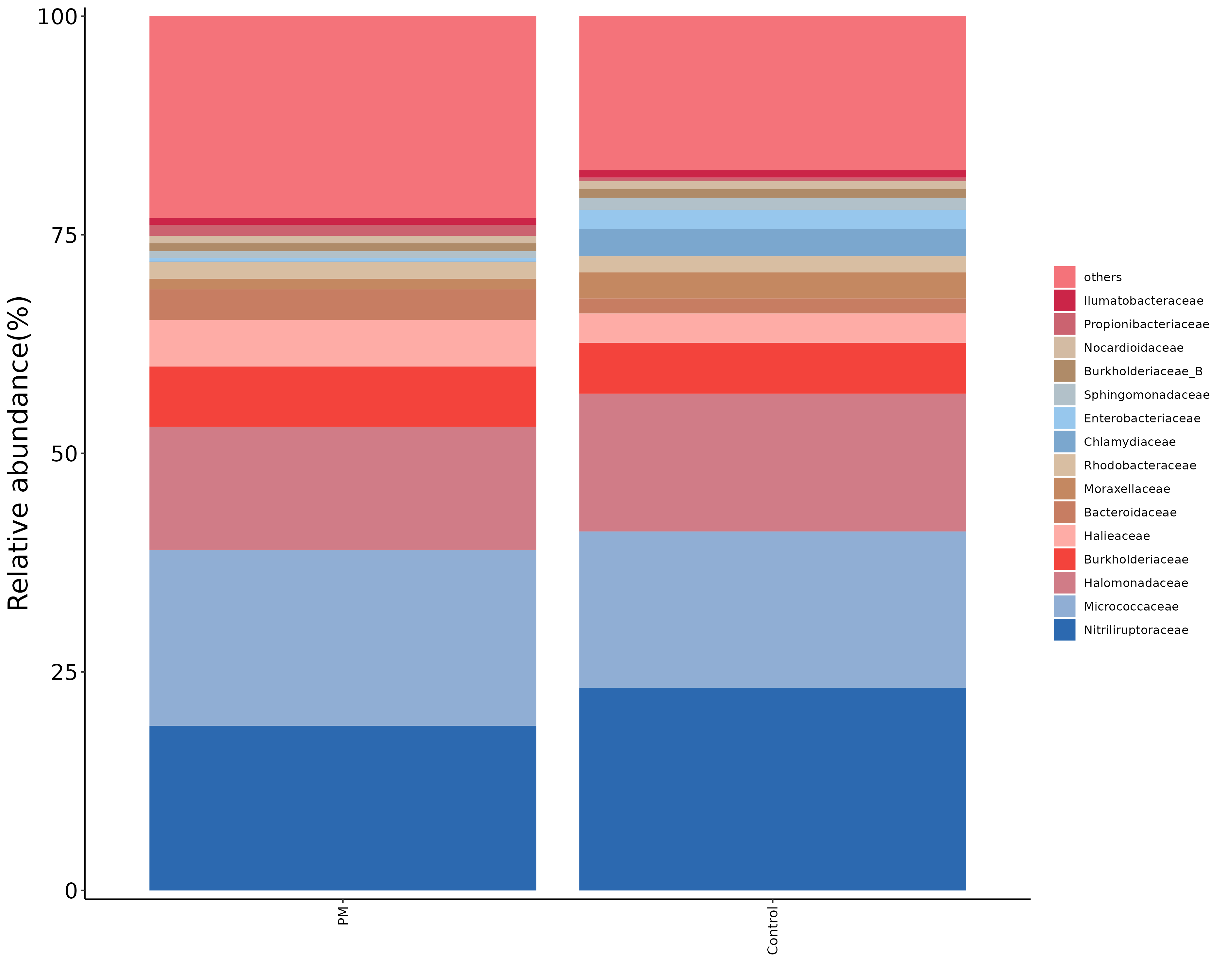

Supplement: Supplementary file 3 [file Data_Sheet_1.zip › 7.Average/1.Community_Structure/barplot/C372089/Family_top15_others.png]

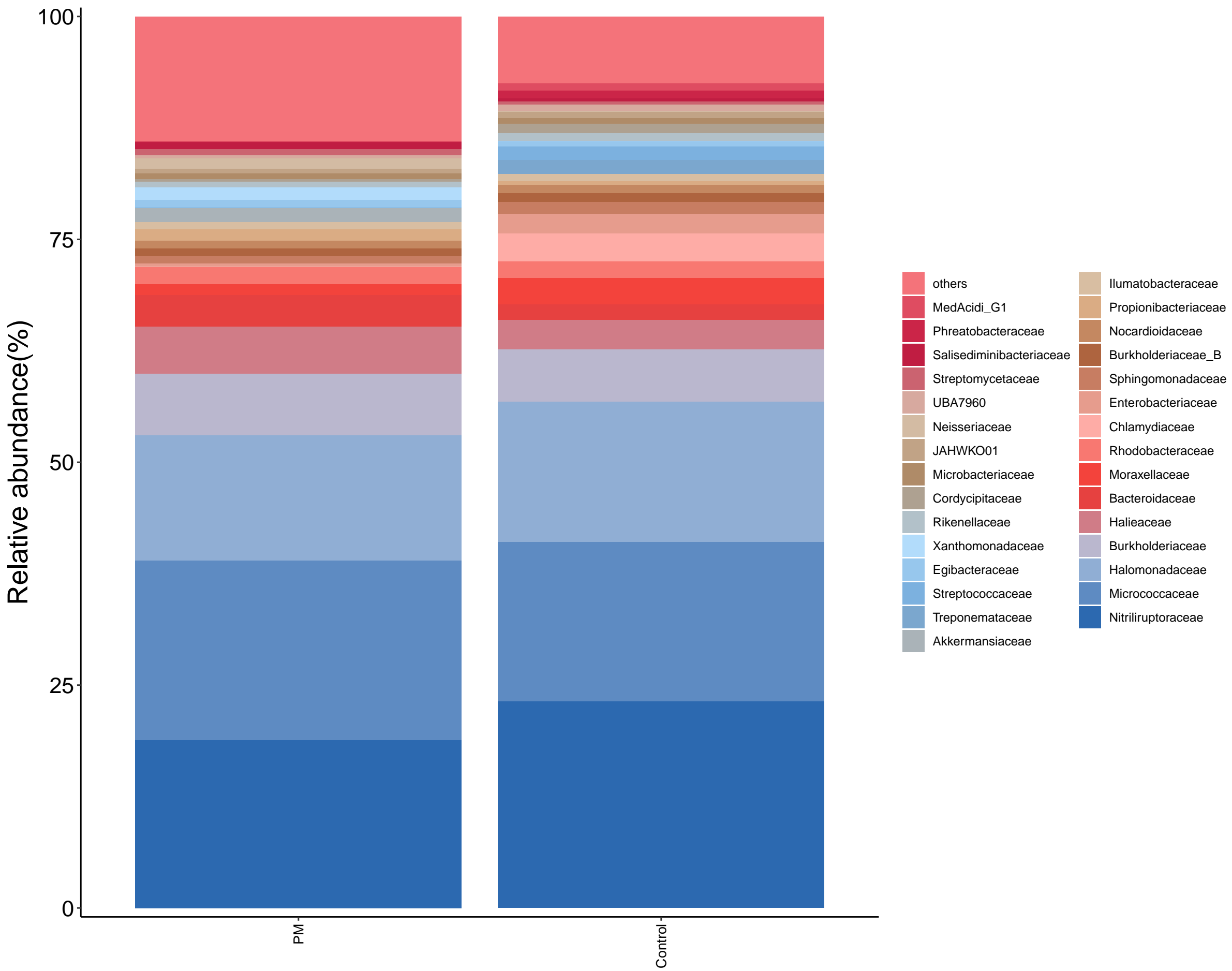

Supplement: Supplementary file 3 [file Data_Sheet_1.zip › 7.Average/1.Community_Structure/barplot/C372089/Family_top30_others.pdf]

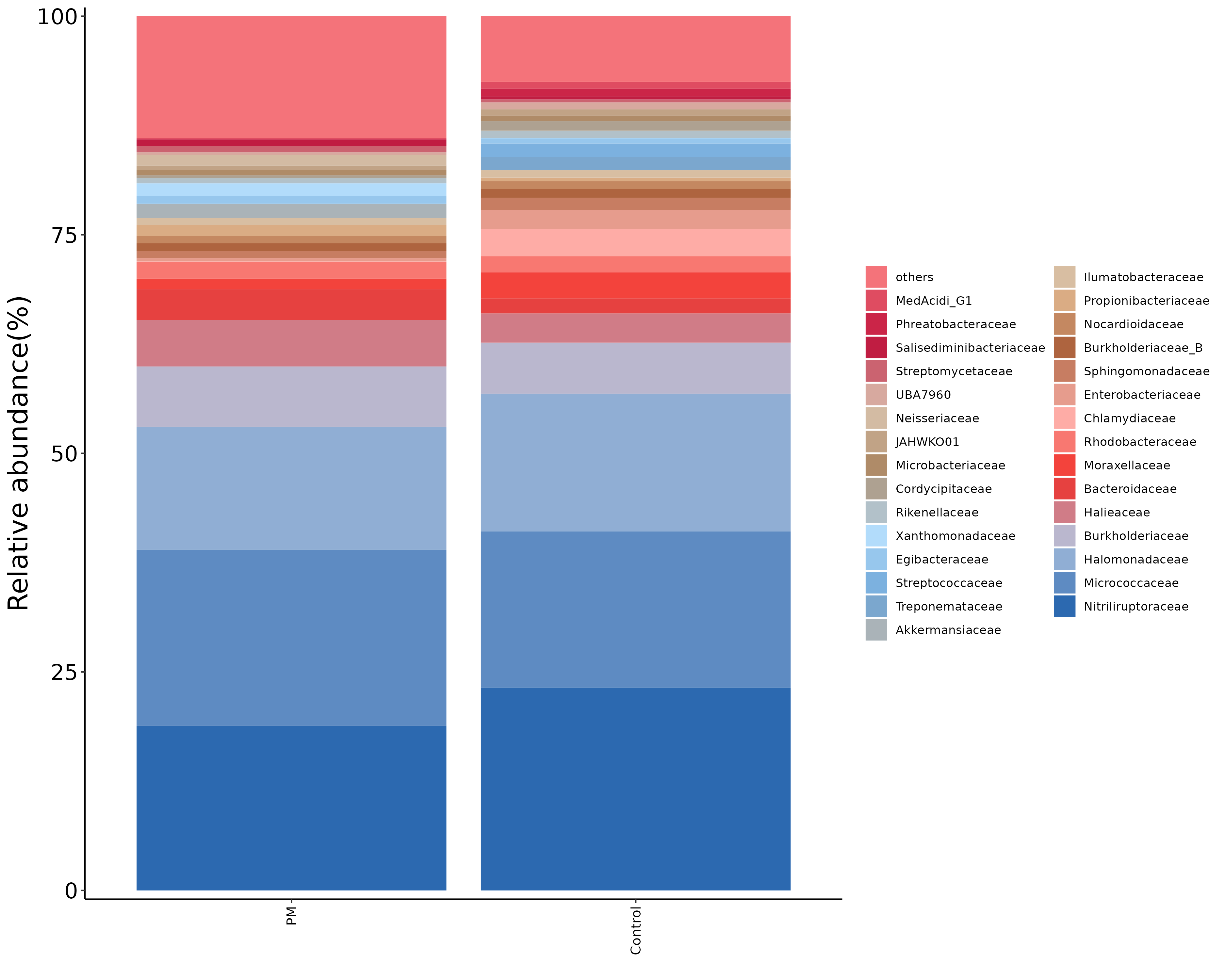

Supplement: Supplementary file 3 [file Data_Sheet_1.zip › 7.Average/1.Community_Structure/barplot/C372089/Family_top30_others.png]

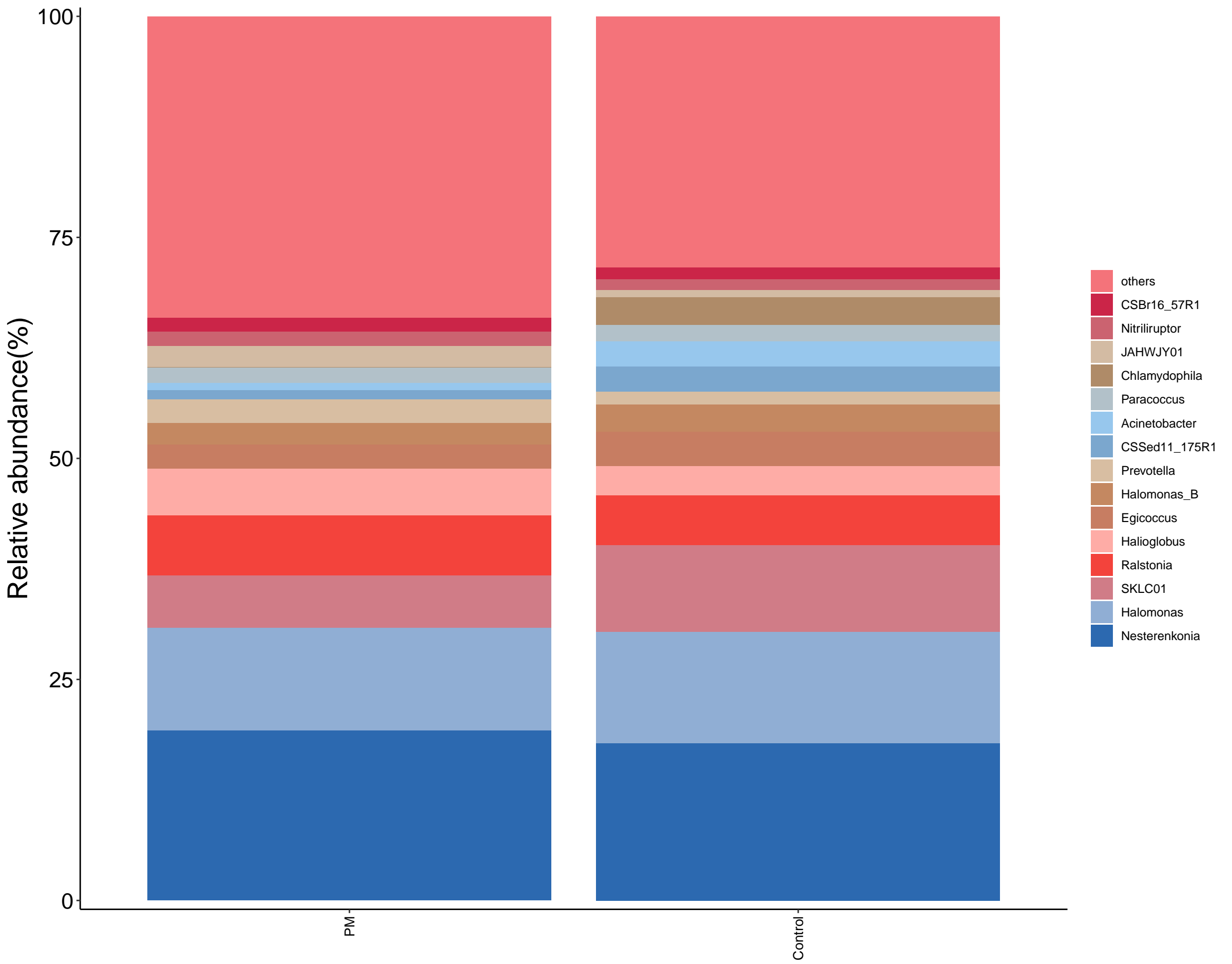

Supplement: Supplementary file 3 [file Data_Sheet_1.zip › 7.Average/1.Community_Structure/barplot/C372089/Genus_top15_others.pdf]

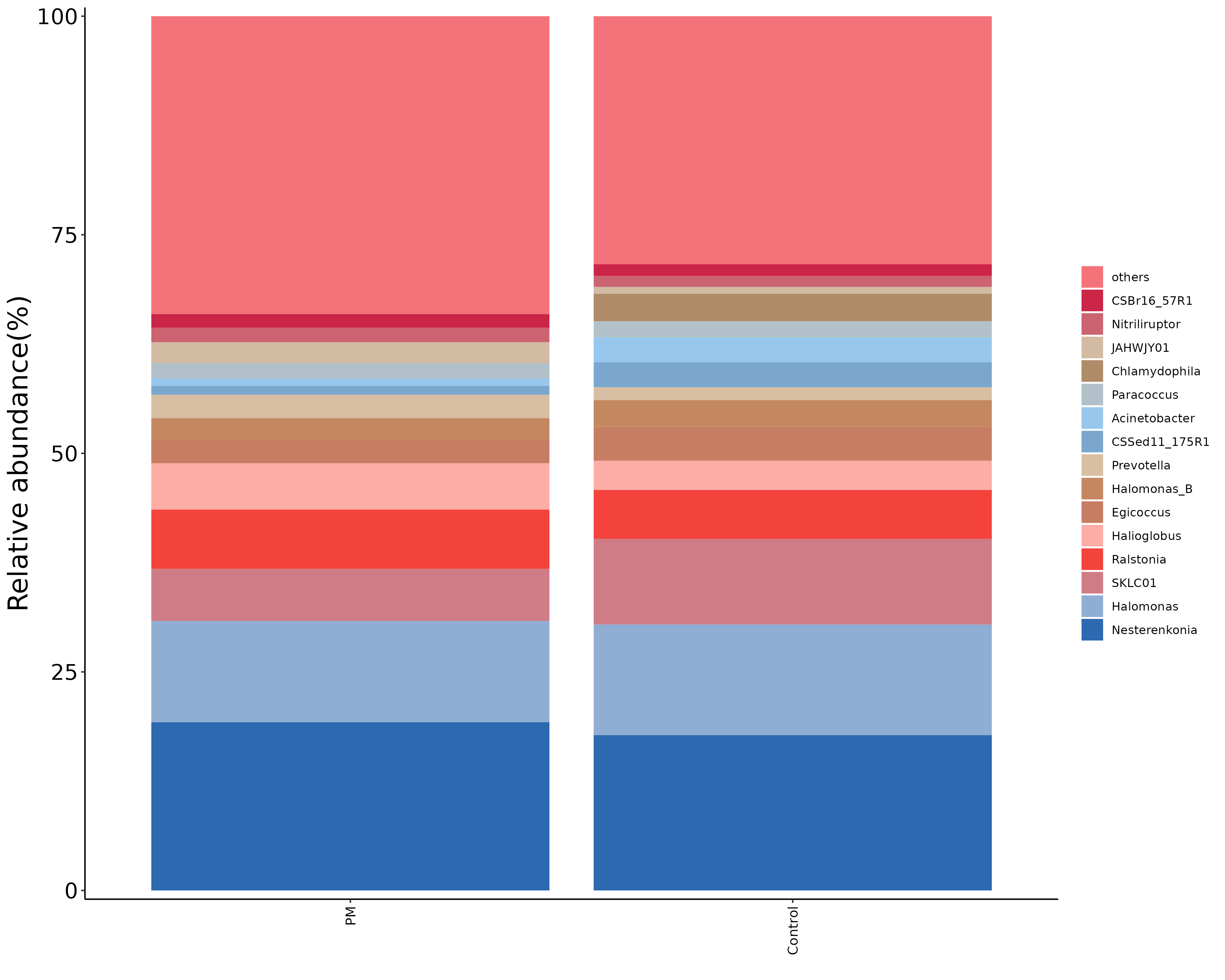

Supplement: Supplementary file 3 [file Data_Sheet_1.zip › 7.Average/1.Community_Structure/barplot/C372089/Genus_top15_others.png]

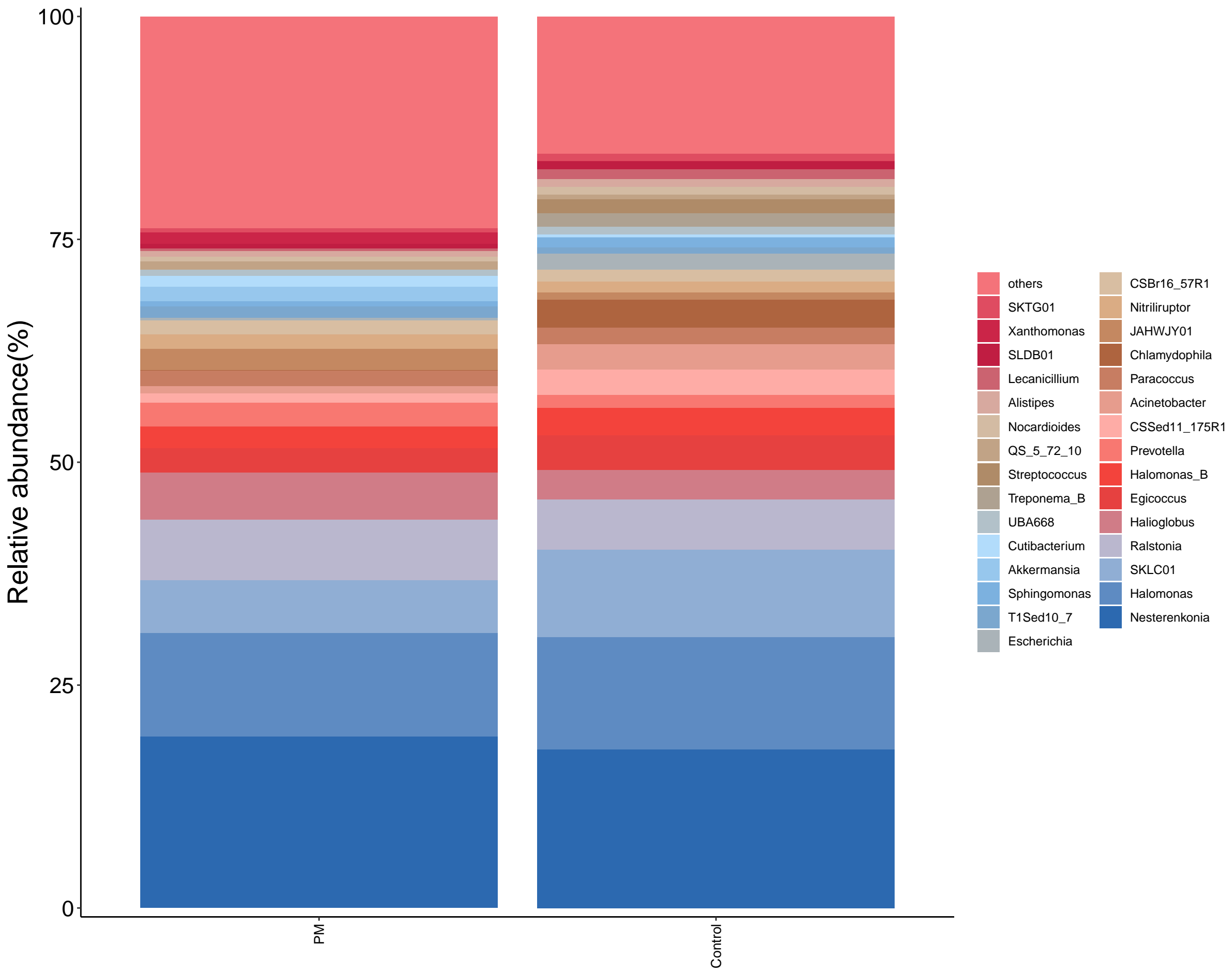

Supplement: Supplementary file 3 [file Data_Sheet_1.zip › 7.Average/1.Community_Structure/barplot/C372089/Genus_top30_others.pdf]

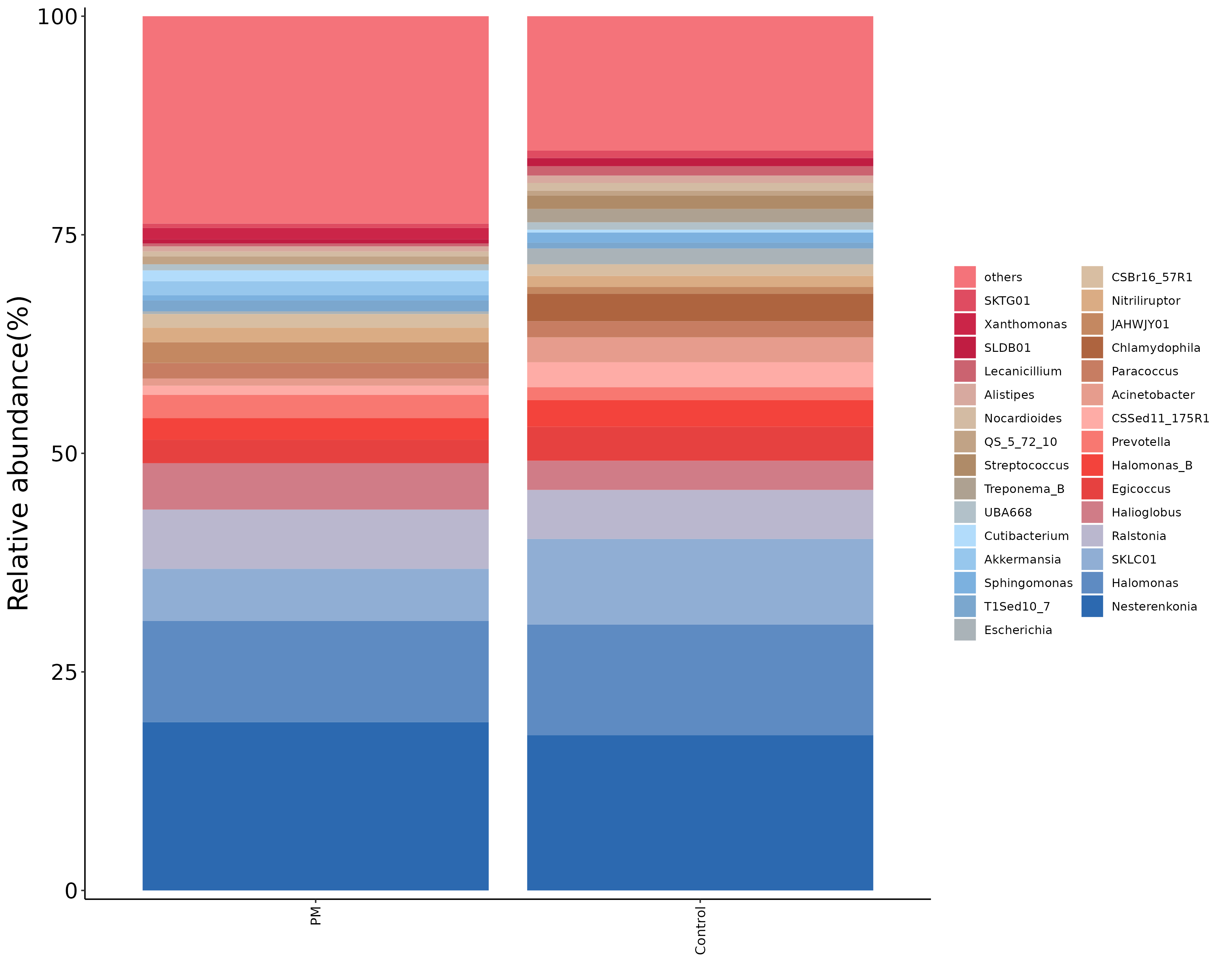

Supplement: Supplementary file 3 [file Data_Sheet_1.zip › 7.Average/1.Community_Structure/barplot/C372089/Genus_top30_others.png]

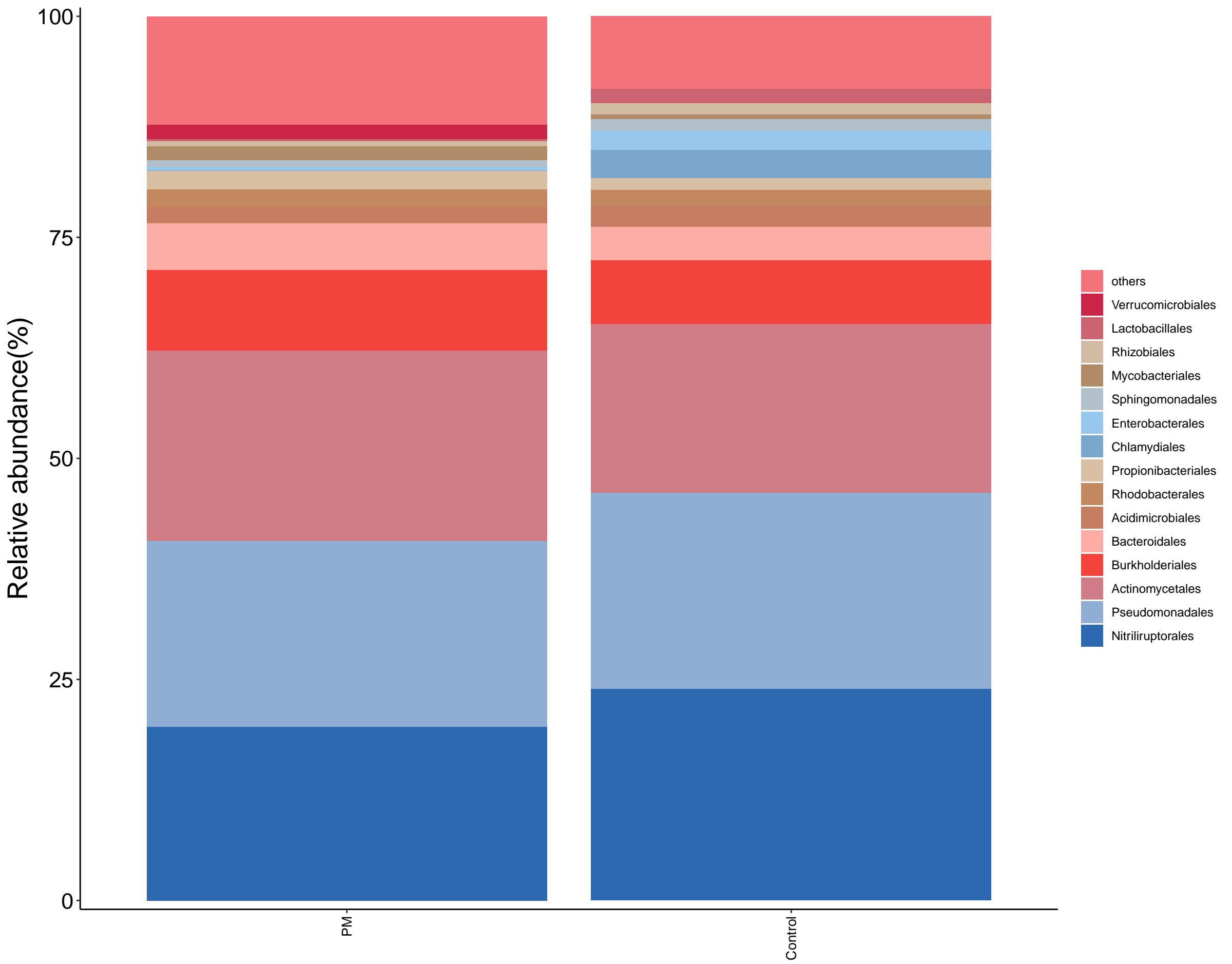

Supplement: Supplementary file 3 [file Data_Sheet_1.zip › 7.Average/1.Community_Structure/barplot/C372089/Order_top15_others.pdf]

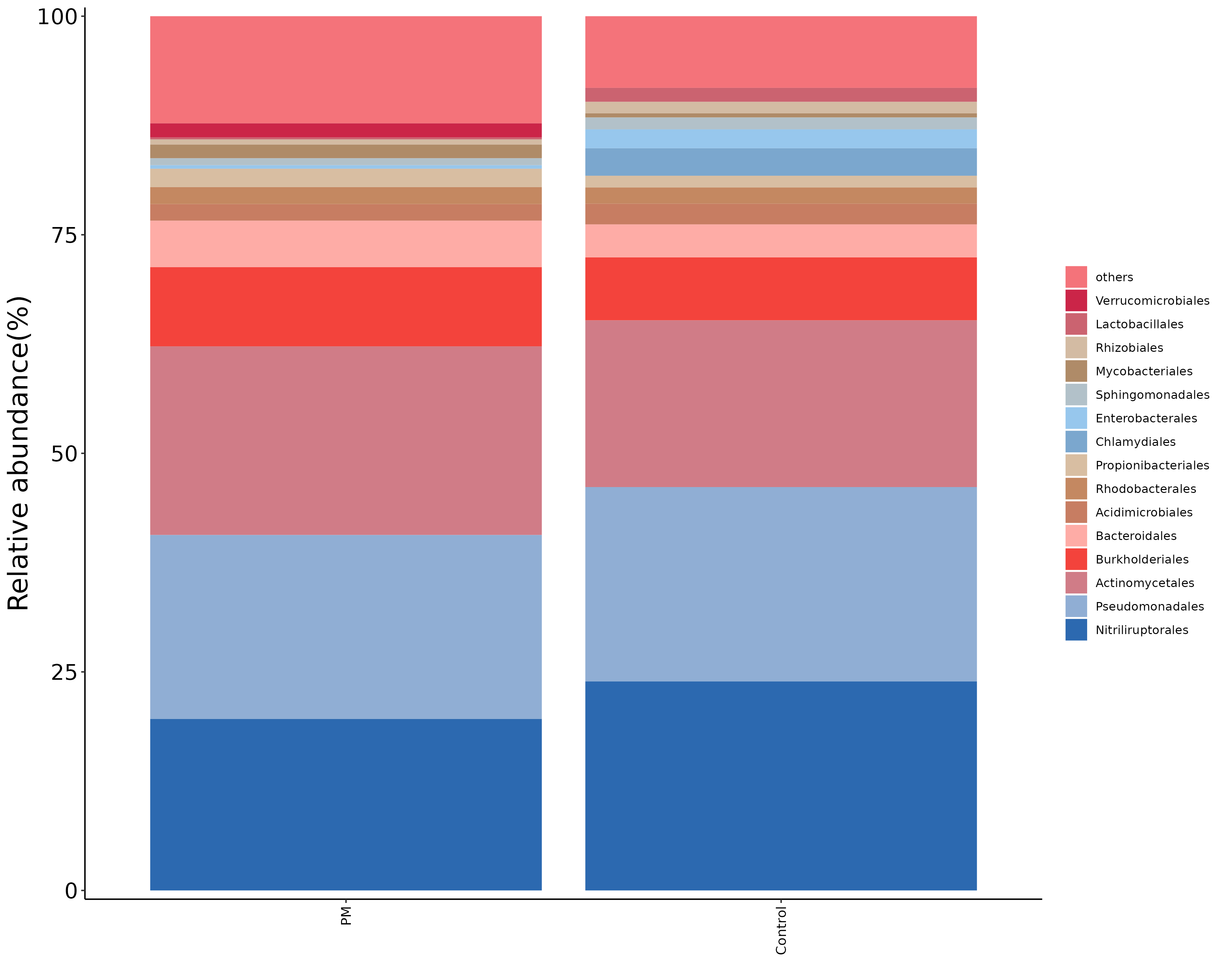

Supplement: Supplementary file 3 [file Data_Sheet_1.zip › 7.Average/1.Community_Structure/barplot/C372089/Order_top15_others.png]

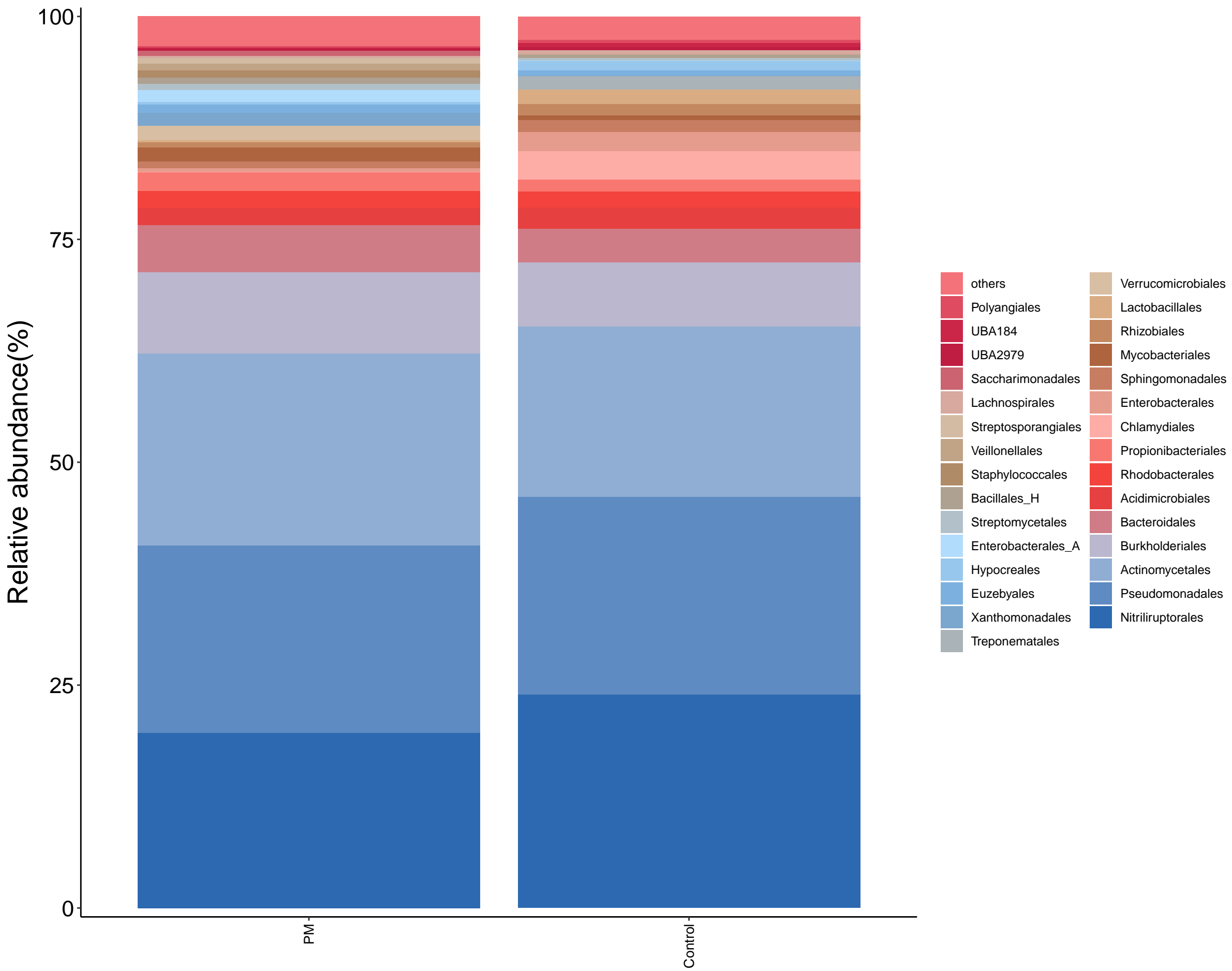

Supplement: Supplementary file 3 [file Data_Sheet_1.zip › 7.Average/1.Community_Structure/barplot/C372089/Order_top30_others.pdf]

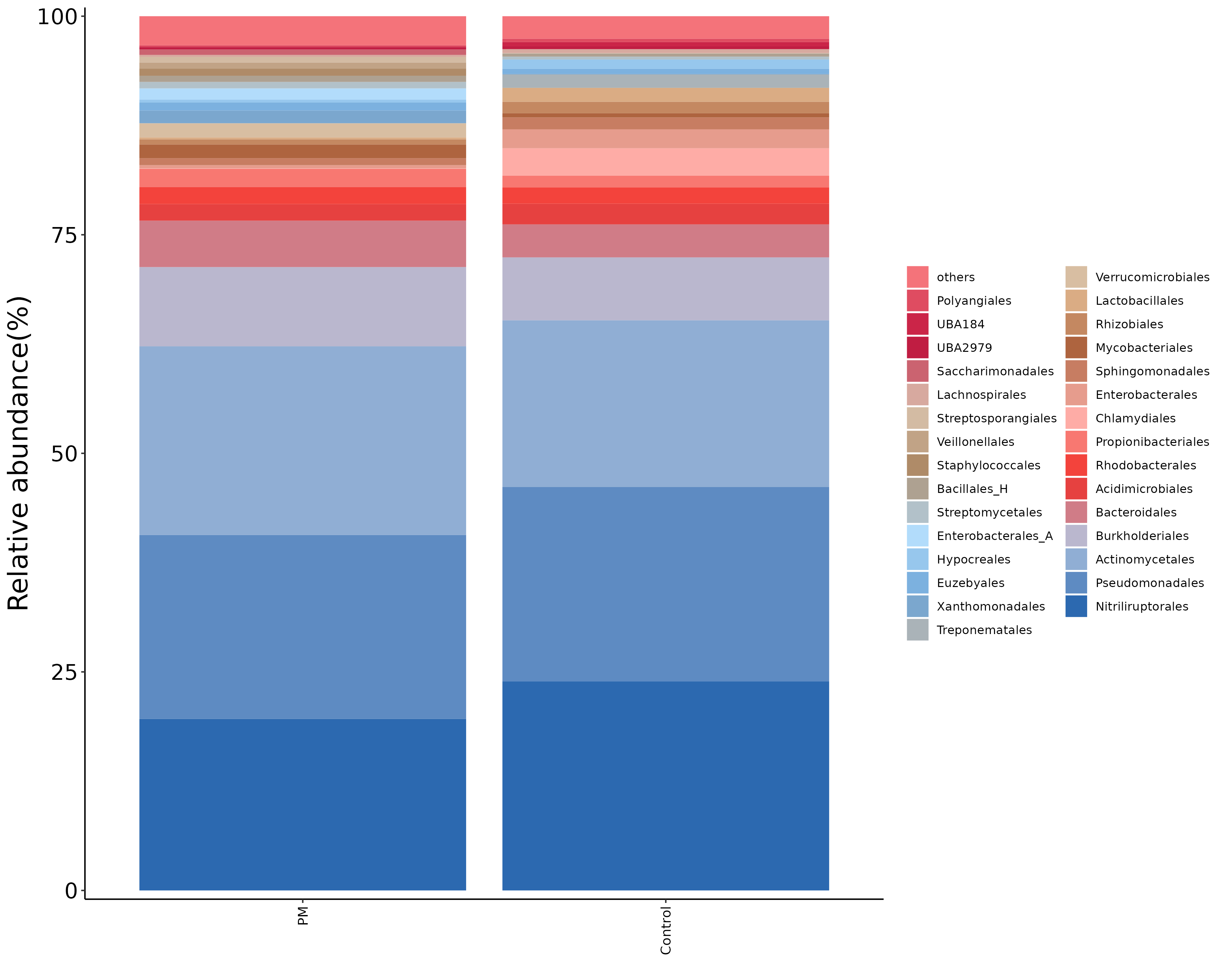

Supplement: Supplementary file 3 [file Data_Sheet_1.zip › 7.Average/1.Community_Structure/barplot/C372089/Order_top30_others.png]

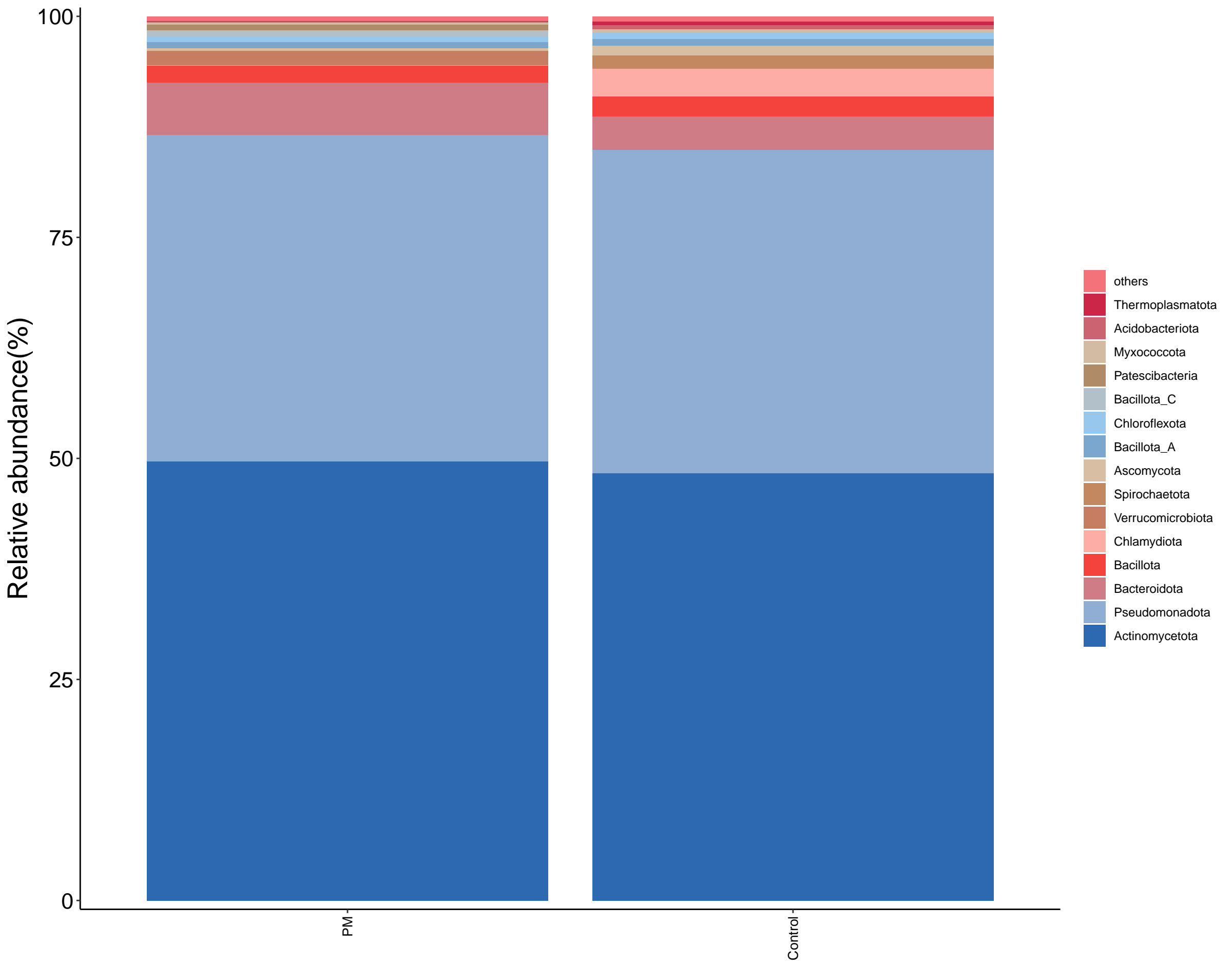

Supplement: Supplementary file 3 [file Data_Sheet_1.zip › 7.Average/1.Community_Structure/barplot/C372089/Phylum_top15_others.pdf]

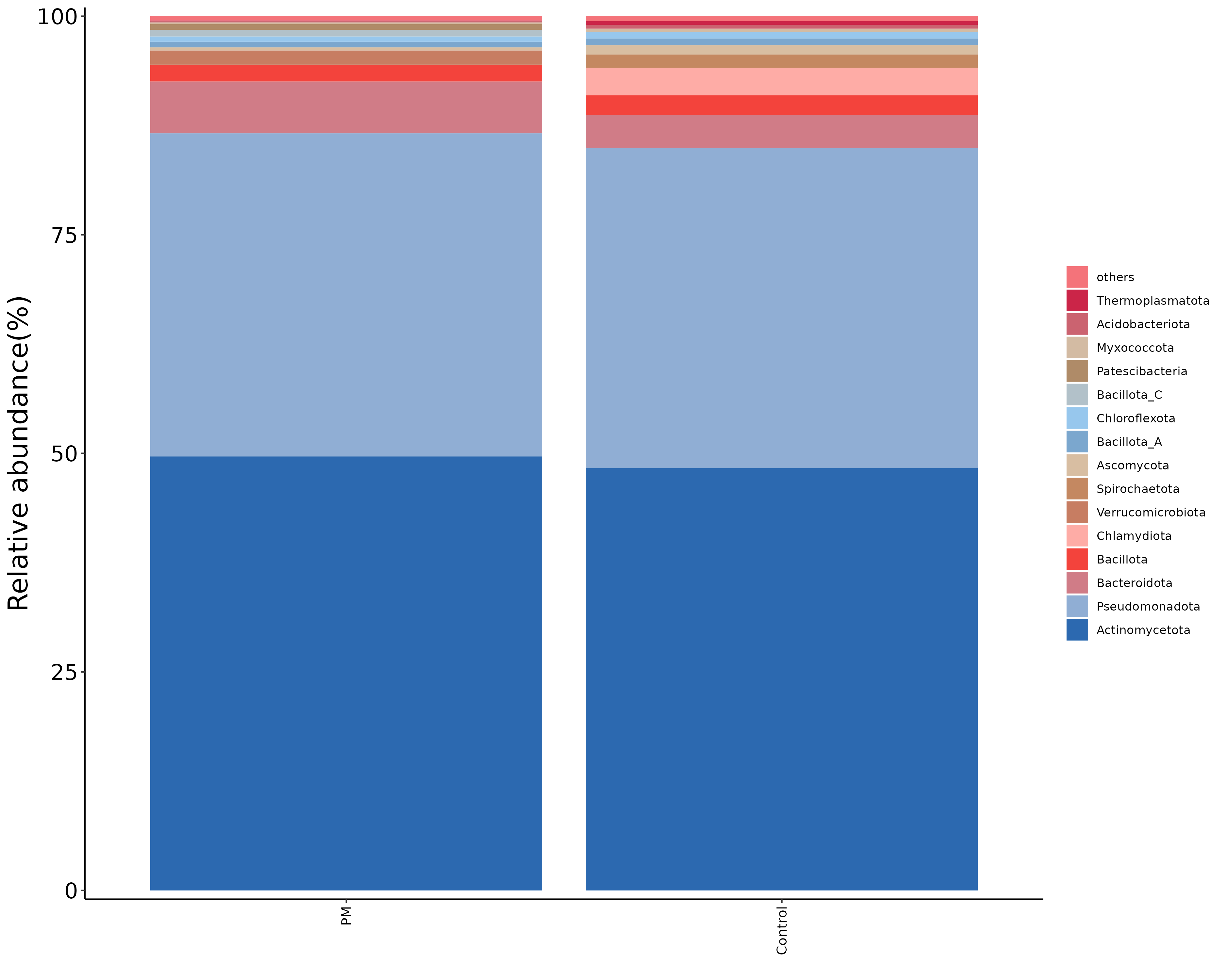

Supplement: Supplementary file 3 [file Data_Sheet_1.zip › 7.Average/1.Community_Structure/barplot/C372089/Phylum_top15_others.png]

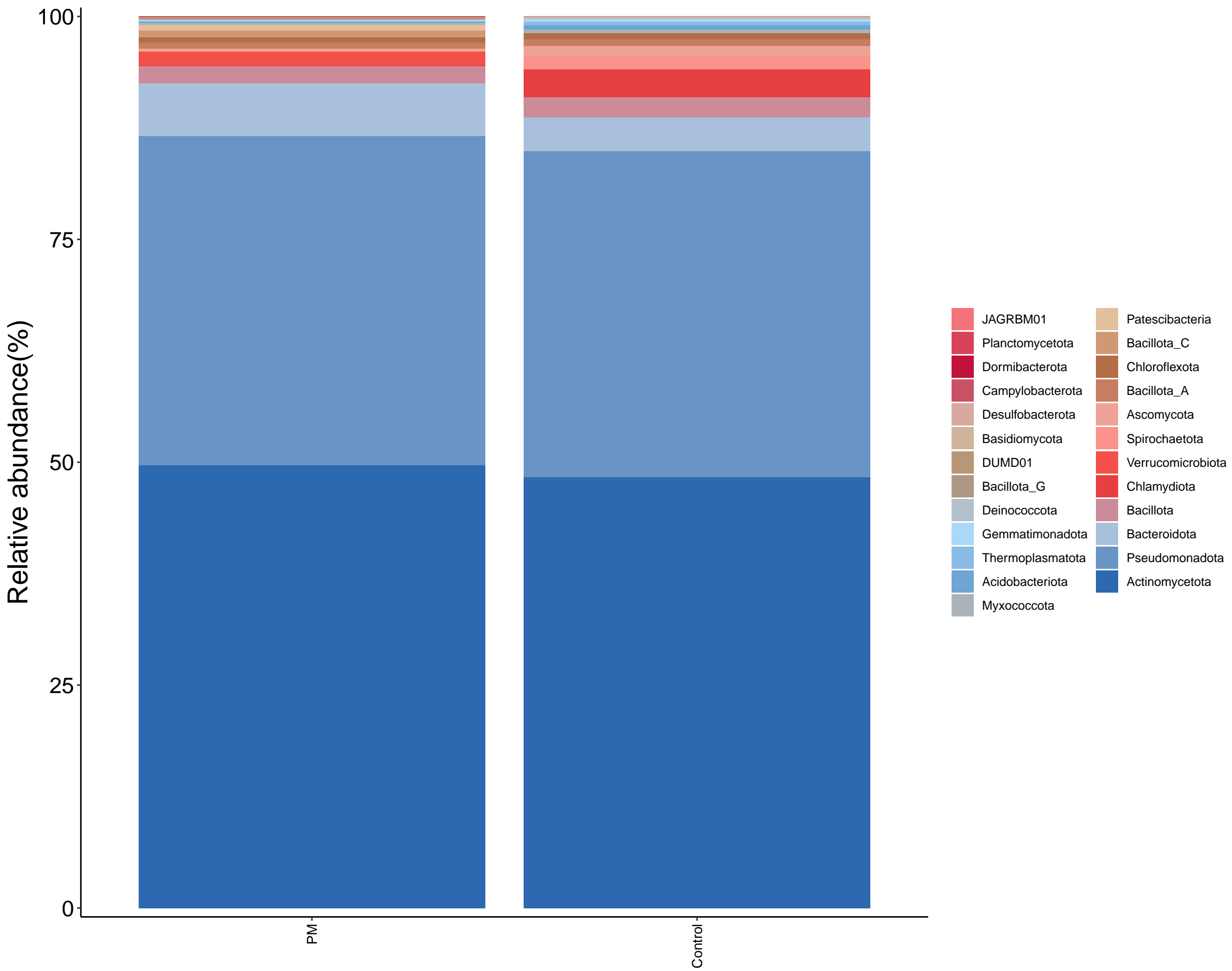

Supplement: Supplementary file 3 [file Data_Sheet_1.zip › 7.Average/1.Community_Structure/barplot/C372089/Phylum_top30_others.pdf]

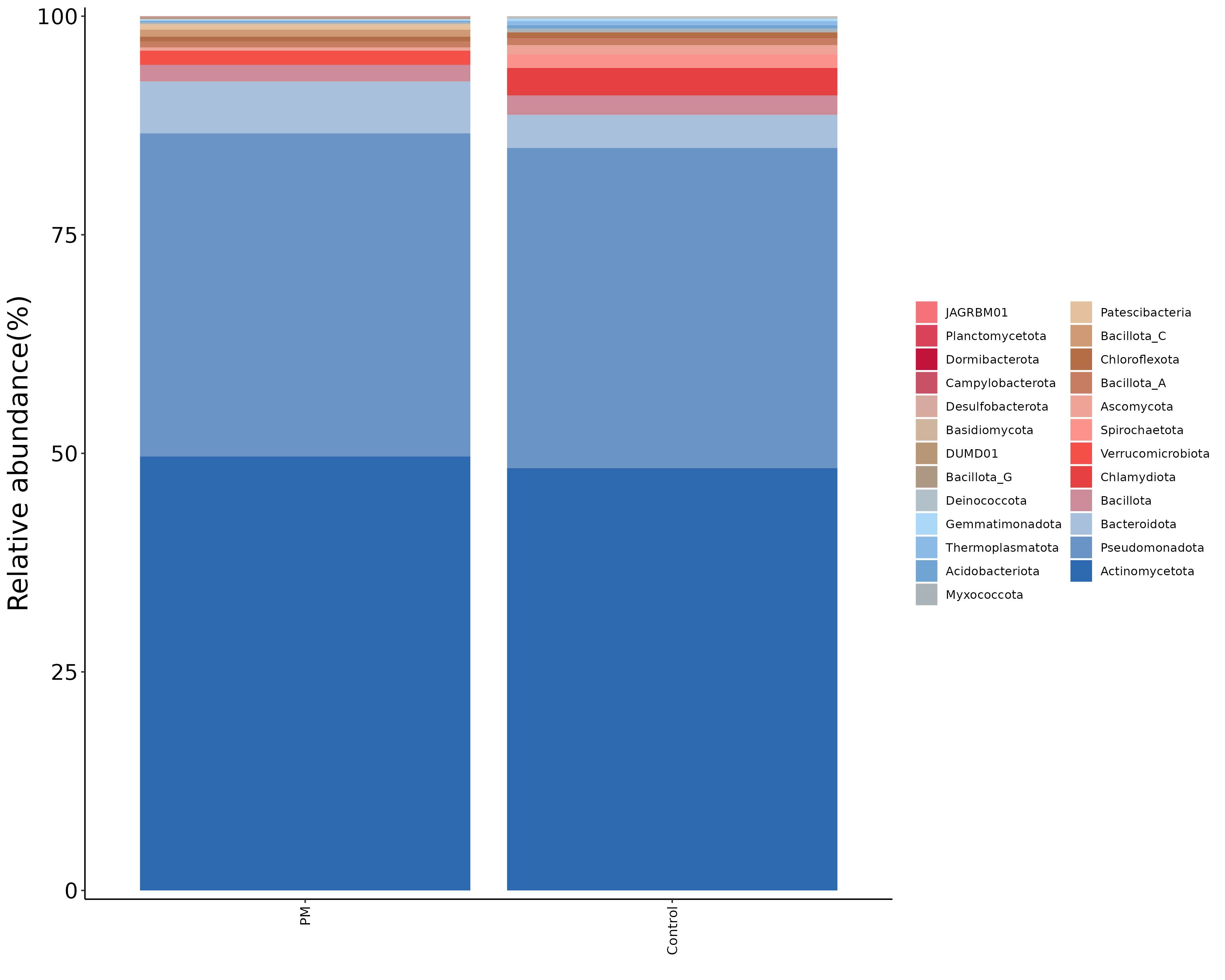

Supplement: Supplementary file 3 [file Data_Sheet_1.zip › 7.Average/1.Community_Structure/barplot/C372089/Phylum_top30_others.png]

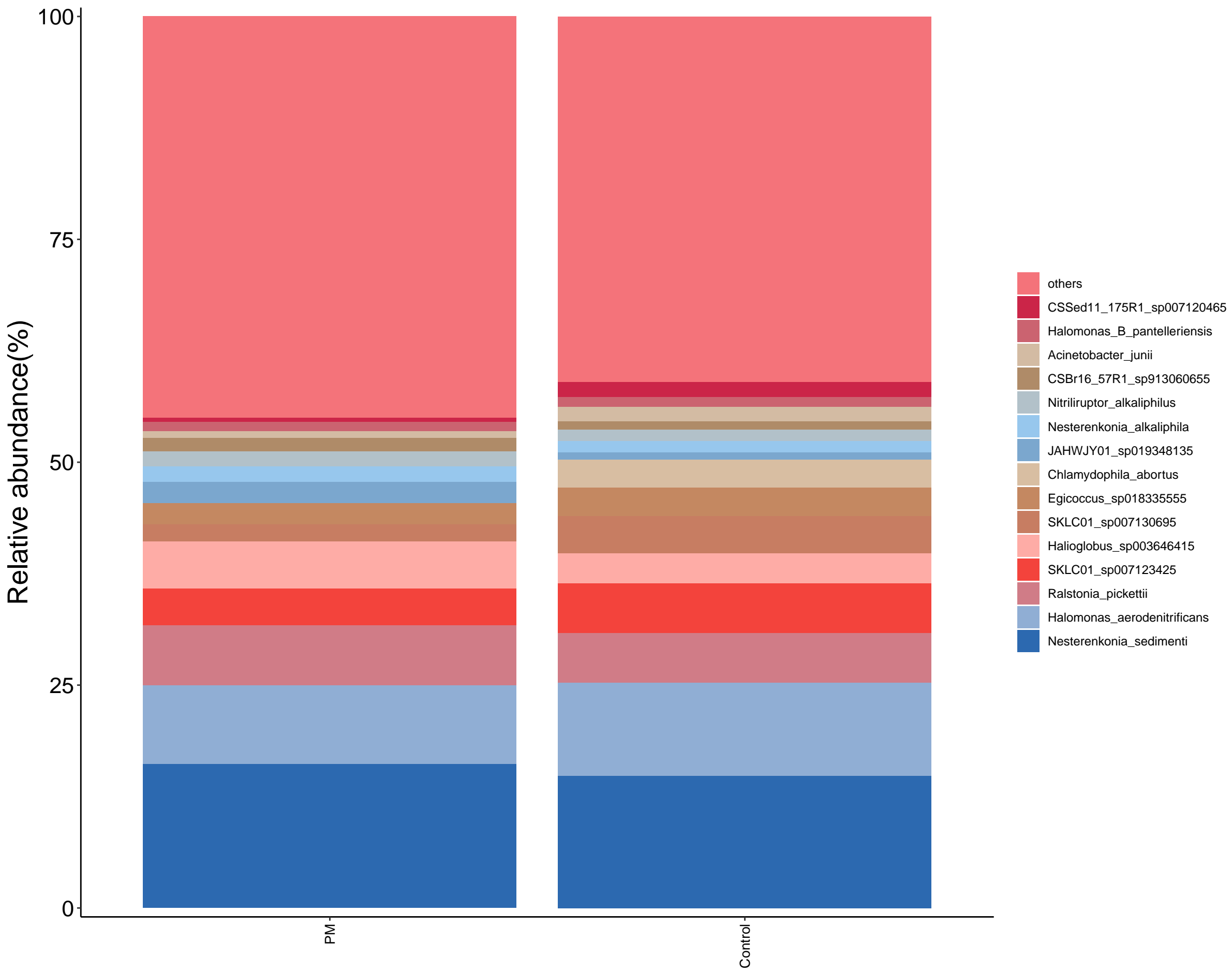

Supplement: Supplementary file 3 [file Data_Sheet_1.zip › 7.Average/1.Community_Structure/barplot/C372089/Species_top15_others.pdf]

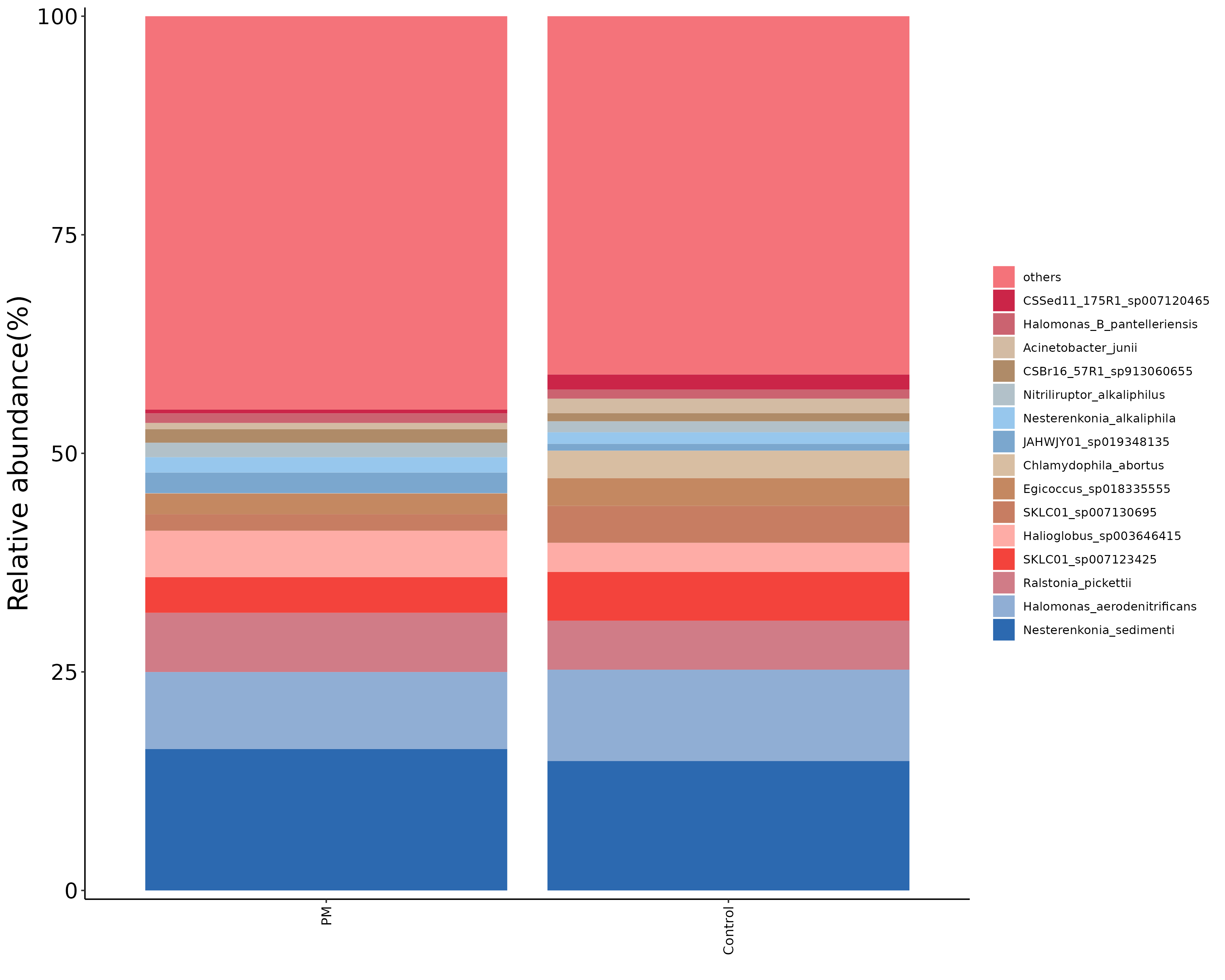

Supplement: Supplementary file 3 [file Data_Sheet_1.zip › 7.Average/1.Community_Structure/barplot/C372089/Species_top15_others.png]

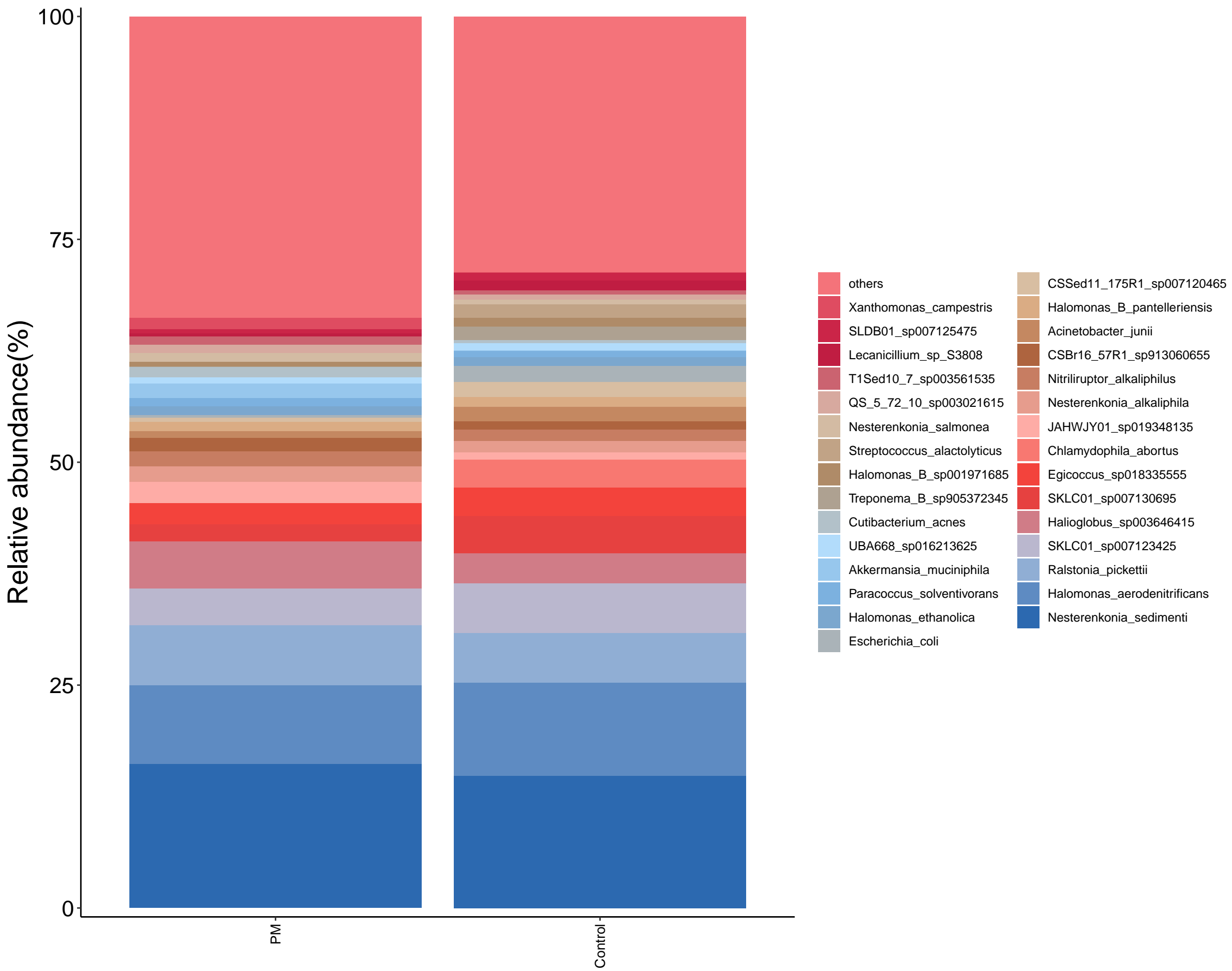

Supplement: Supplementary file 3 [file Data_Sheet_1.zip › 7.Average/1.Community_Structure/barplot/C372089/Species_top30_others.pdf]

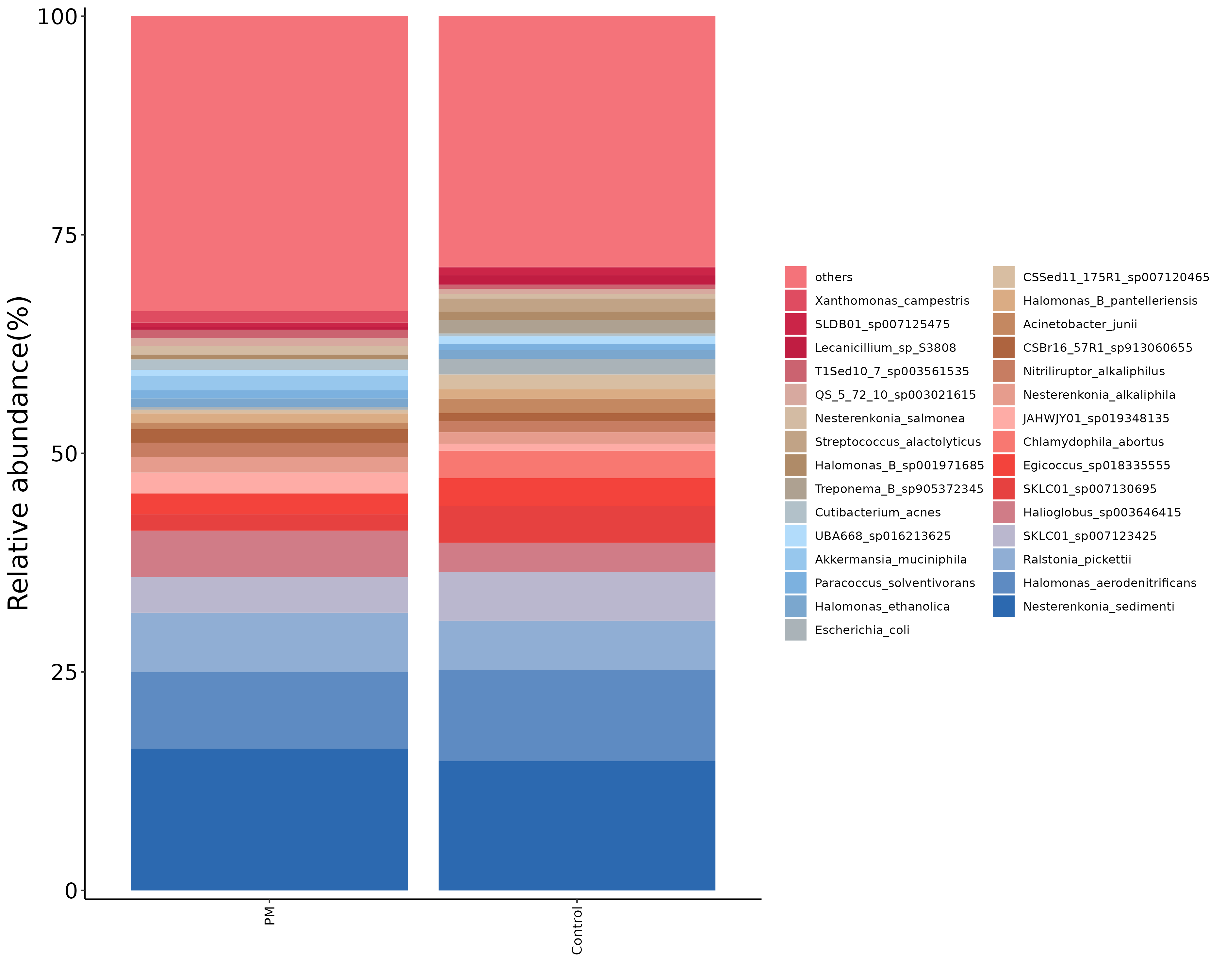

Supplement: Supplementary file 3 [file Data_Sheet_1.zip › 7.Average/1.Community_Structure/barplot/C372089/Species_top30_others.png]

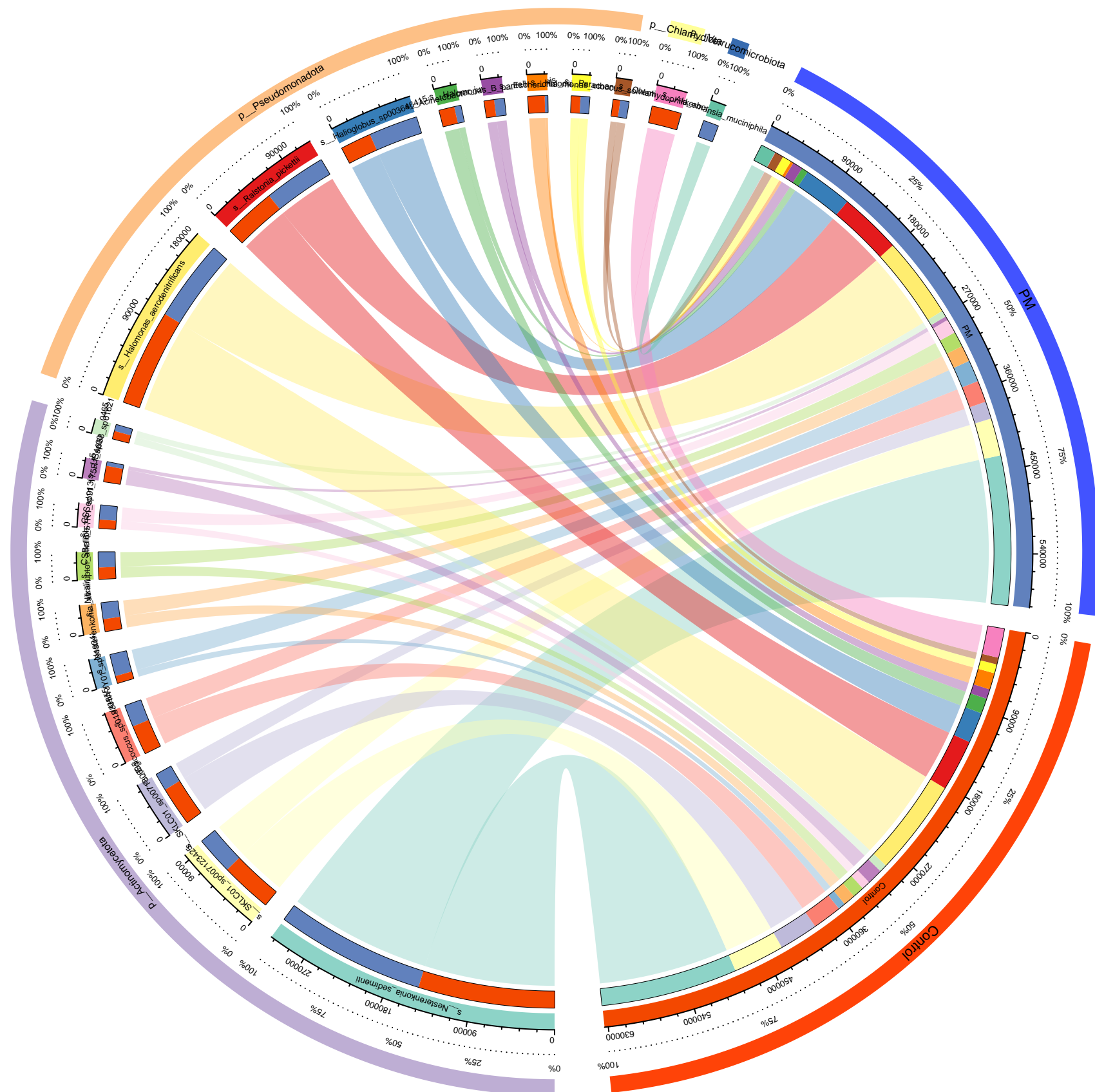

Supplement: Supplementary file 3 [file Data_Sheet_1.zip › 7.Average/1.Community_Structure/Circos/C372089/circlize_plot.pdf]

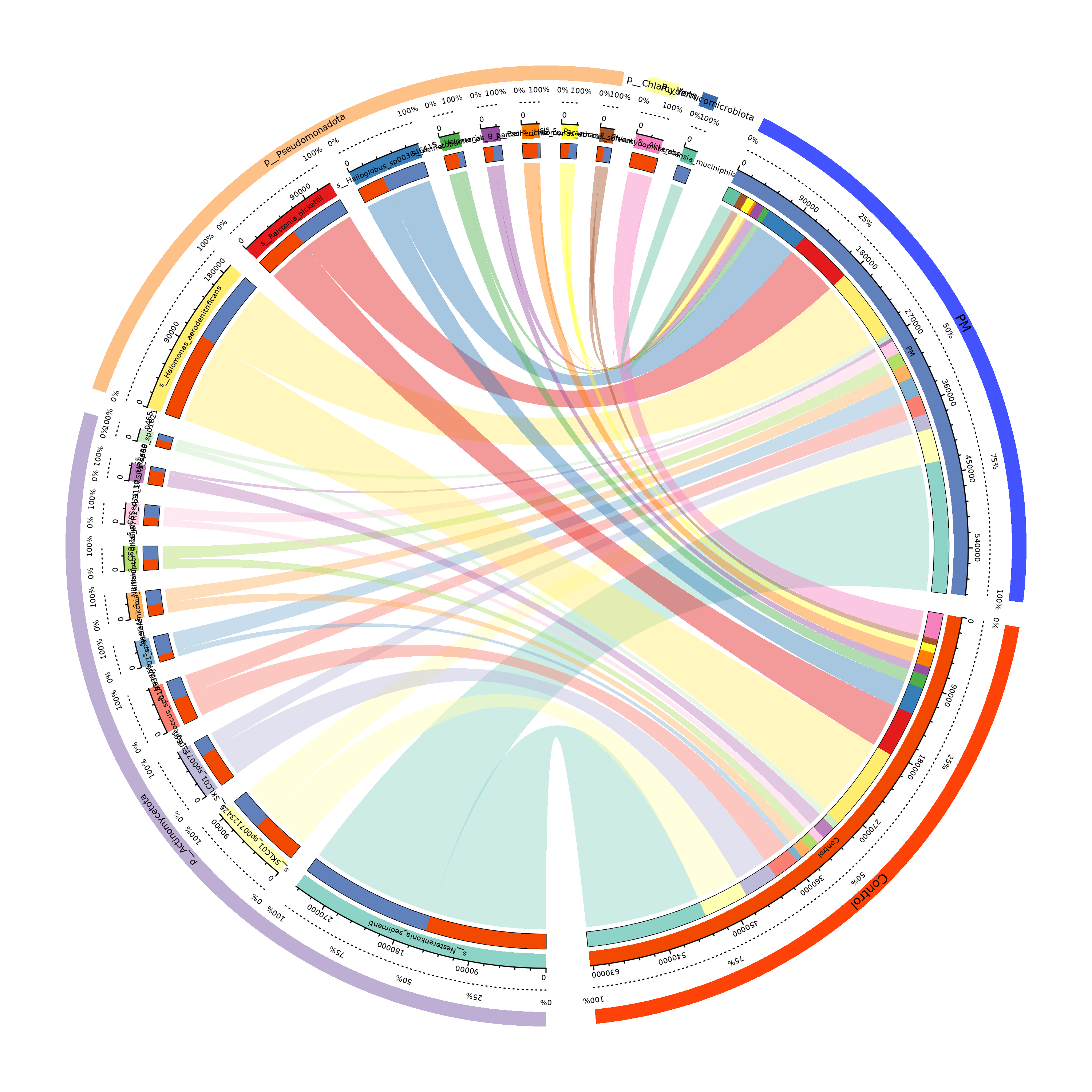

Supplement: Supplementary file 3 [file Data_Sheet_1.zip › 7.Average/1.Community_Structure/Circos/C372089/circlize_plot.png]

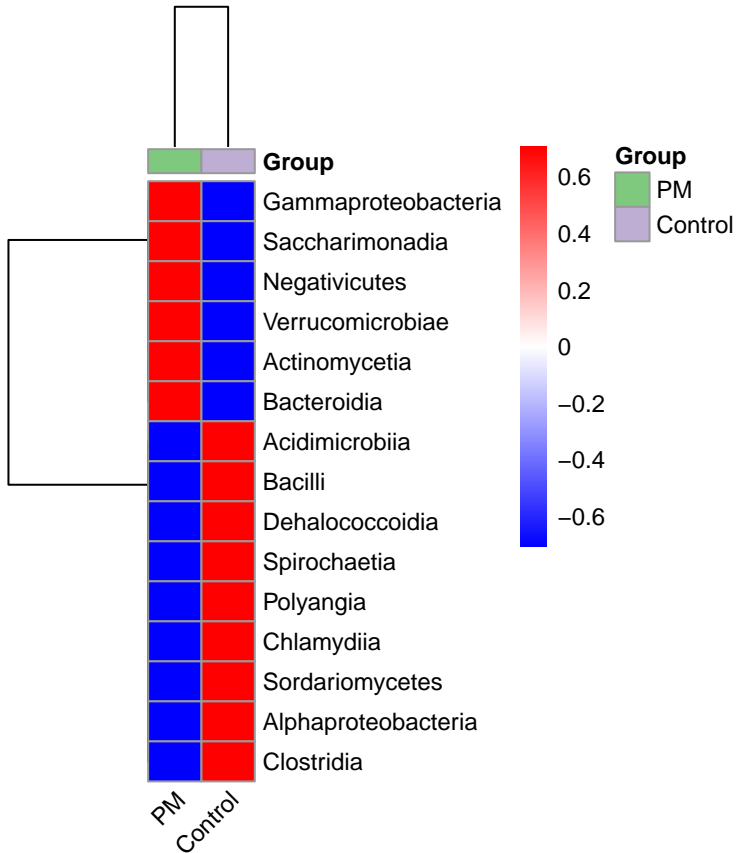

Supplement: Supplementary file 3 [file Data_Sheet_1.zip › 7.Average/1.Community_Structure/heatmap/C372089/Class_top15_cluster.pdf]

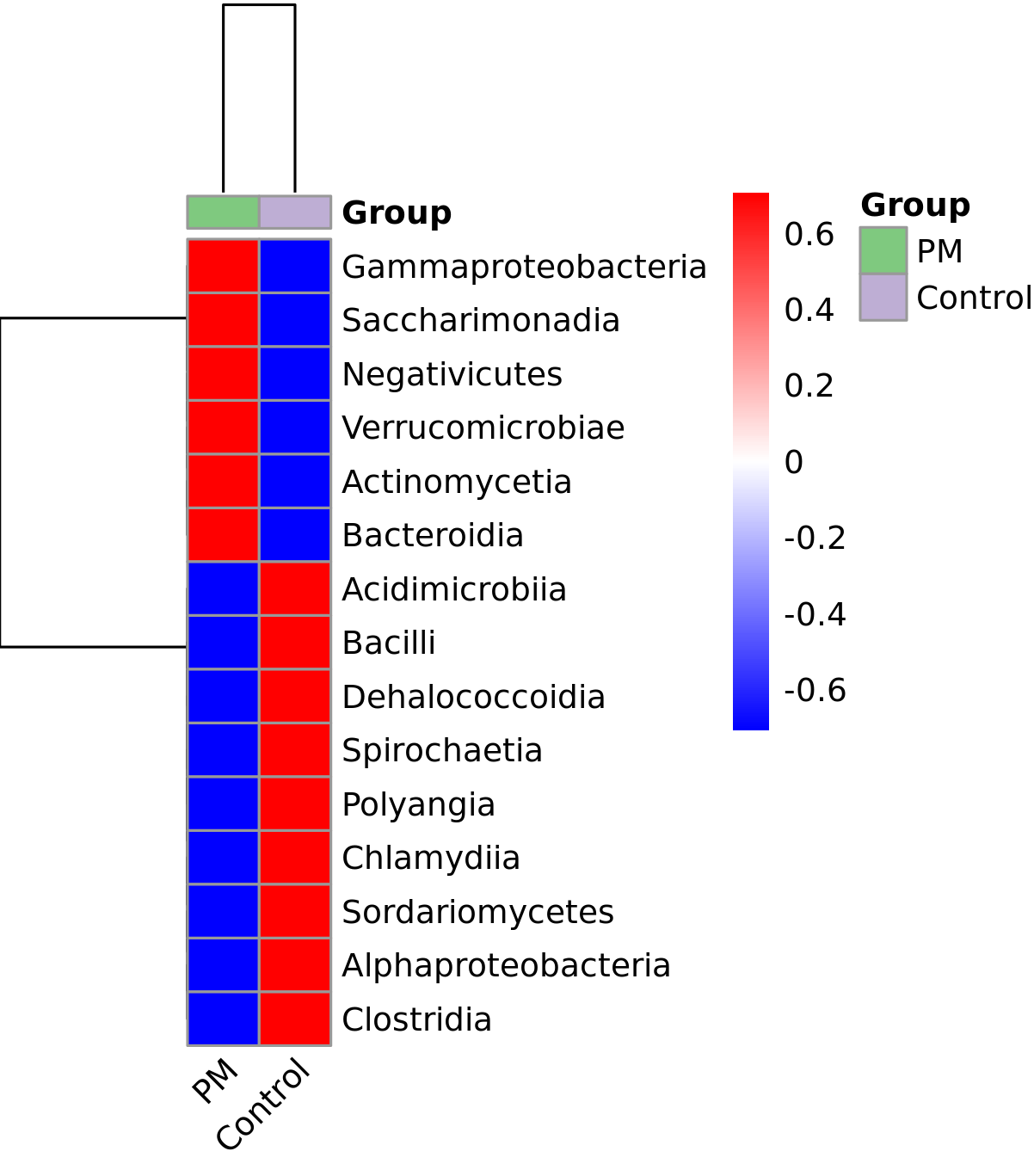

Supplement: Supplementary file 3 [file Data_Sheet_1.zip › 7.Average/1.Community_Structure/heatmap/C372089/Class_top15_cluster.png]

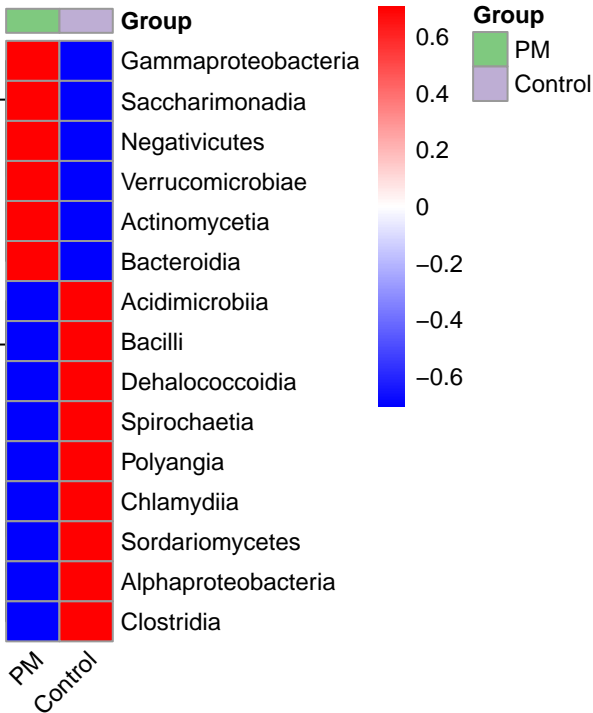

Supplement: Supplementary file 3 [file Data_Sheet_1.zip › 7.Average/1.Community_Structure/heatmap/C372089/Class_top15_nocluster.pdf]

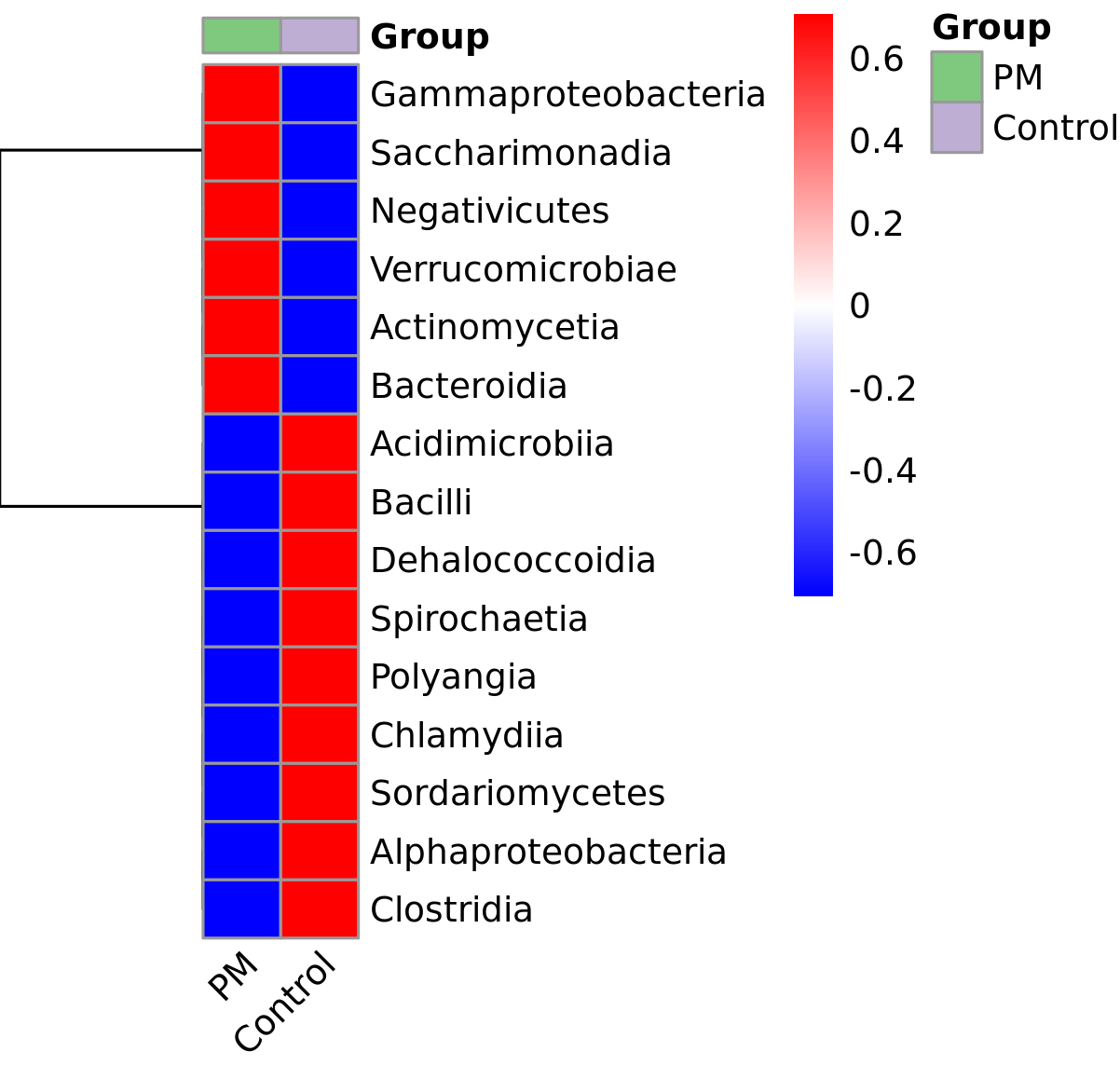

Supplement: Supplementary file 3 [file Data_Sheet_1.zip › 7.Average/1.Community_Structure/heatmap/C372089/Class_top15_nocluster.png]

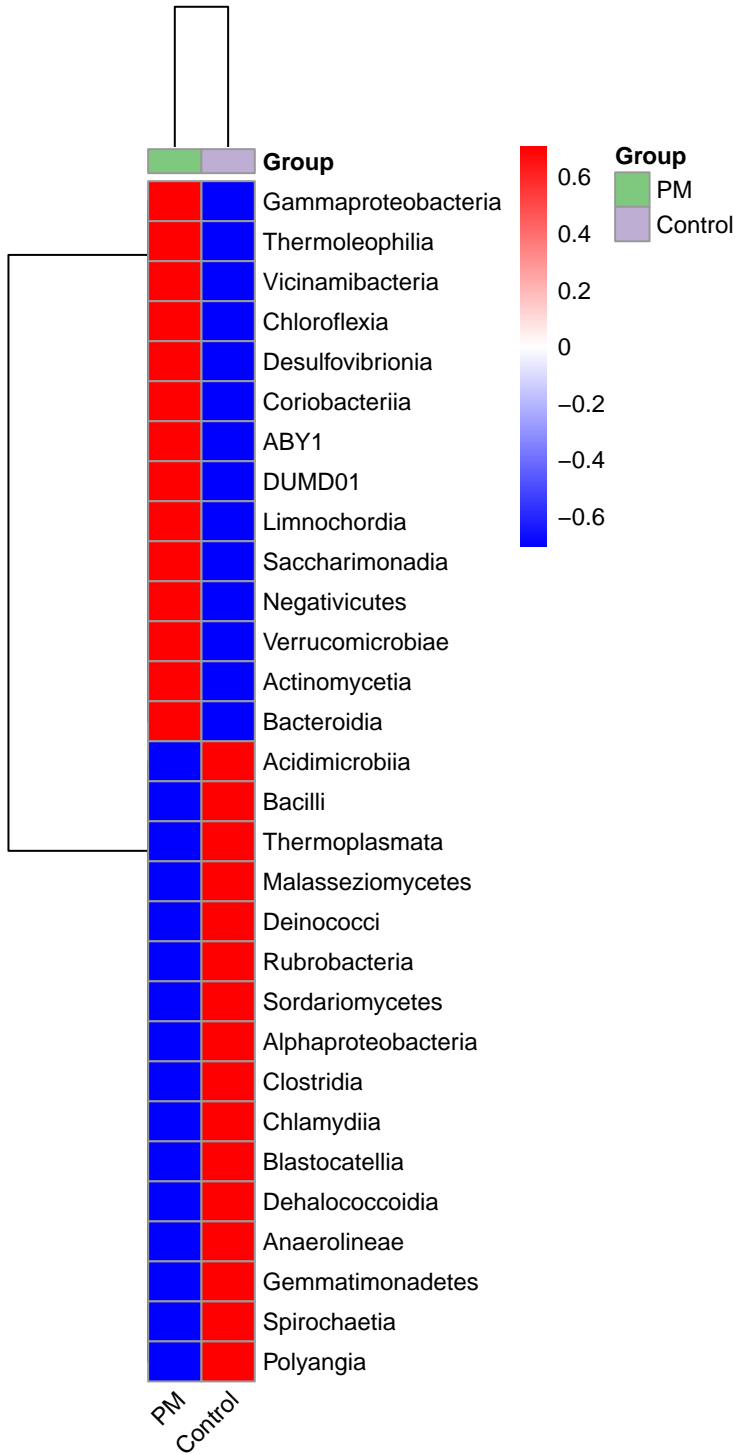

Supplement: Supplementary file 3 [file Data_Sheet_1.zip › 7.Average/1.Community_Structure/heatmap/C372089/Class_top30_cluster.pdf]

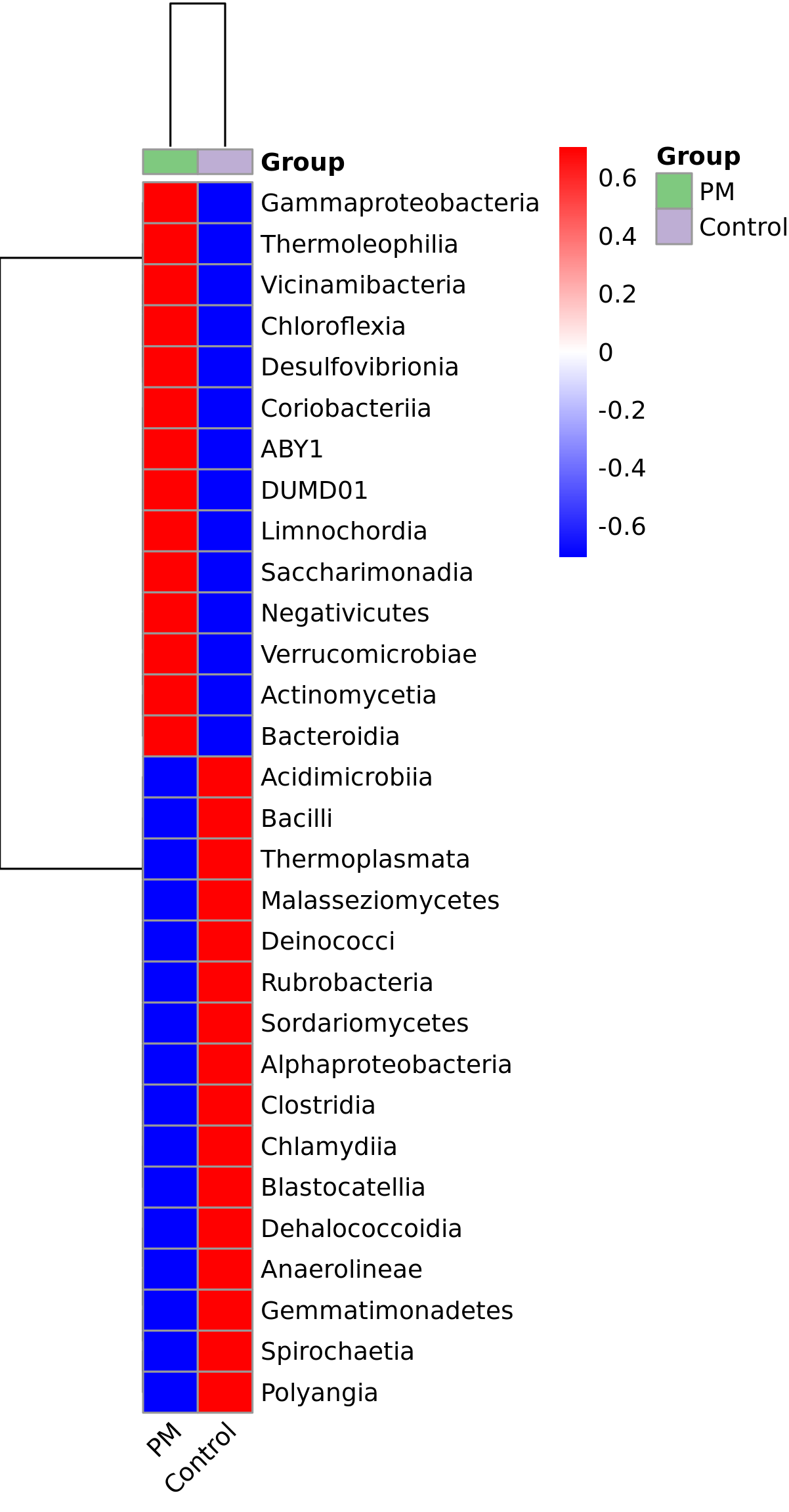

Supplement: Supplementary file 3 [file Data_Sheet_1.zip › 7.Average/1.Community_Structure/heatmap/C372089/Class_top30_cluster.png]

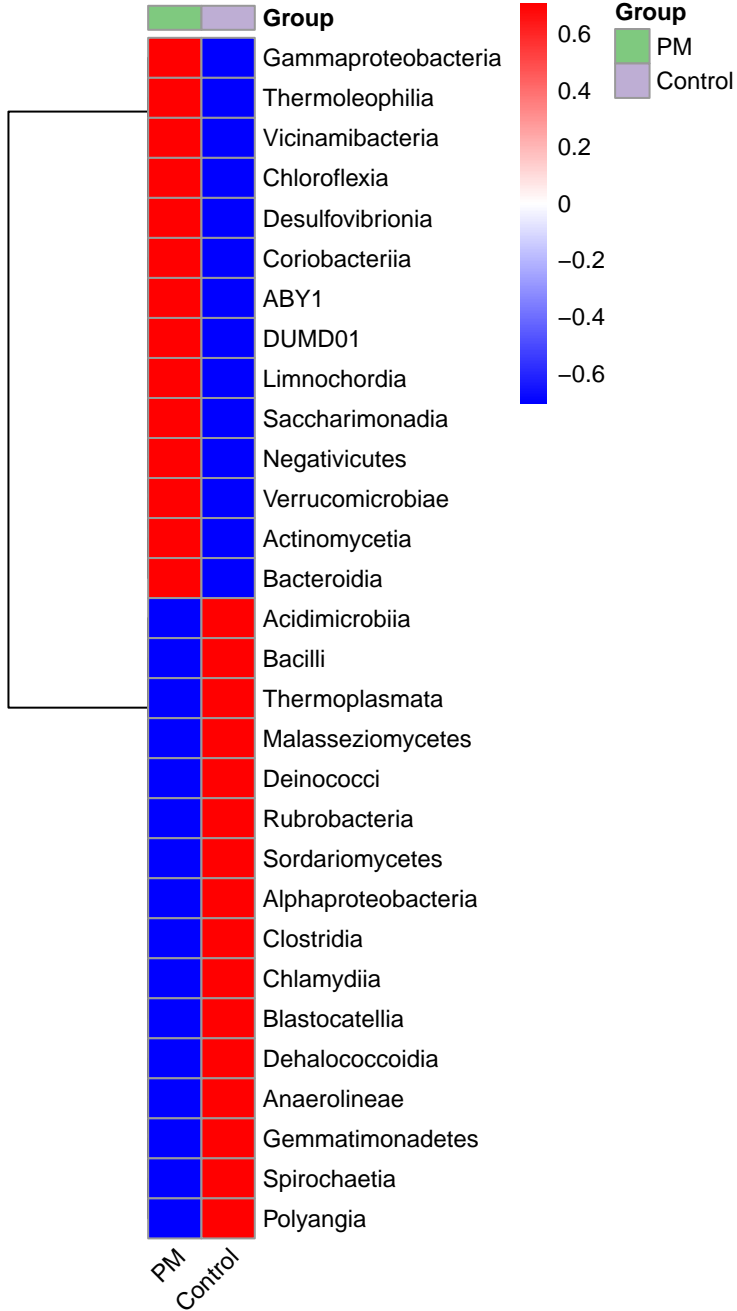

Supplement: Supplementary file 3 [file Data_Sheet_1.zip › 7.Average/1.Community_Structure/heatmap/C372089/Class_top30_nocluster.pdf]

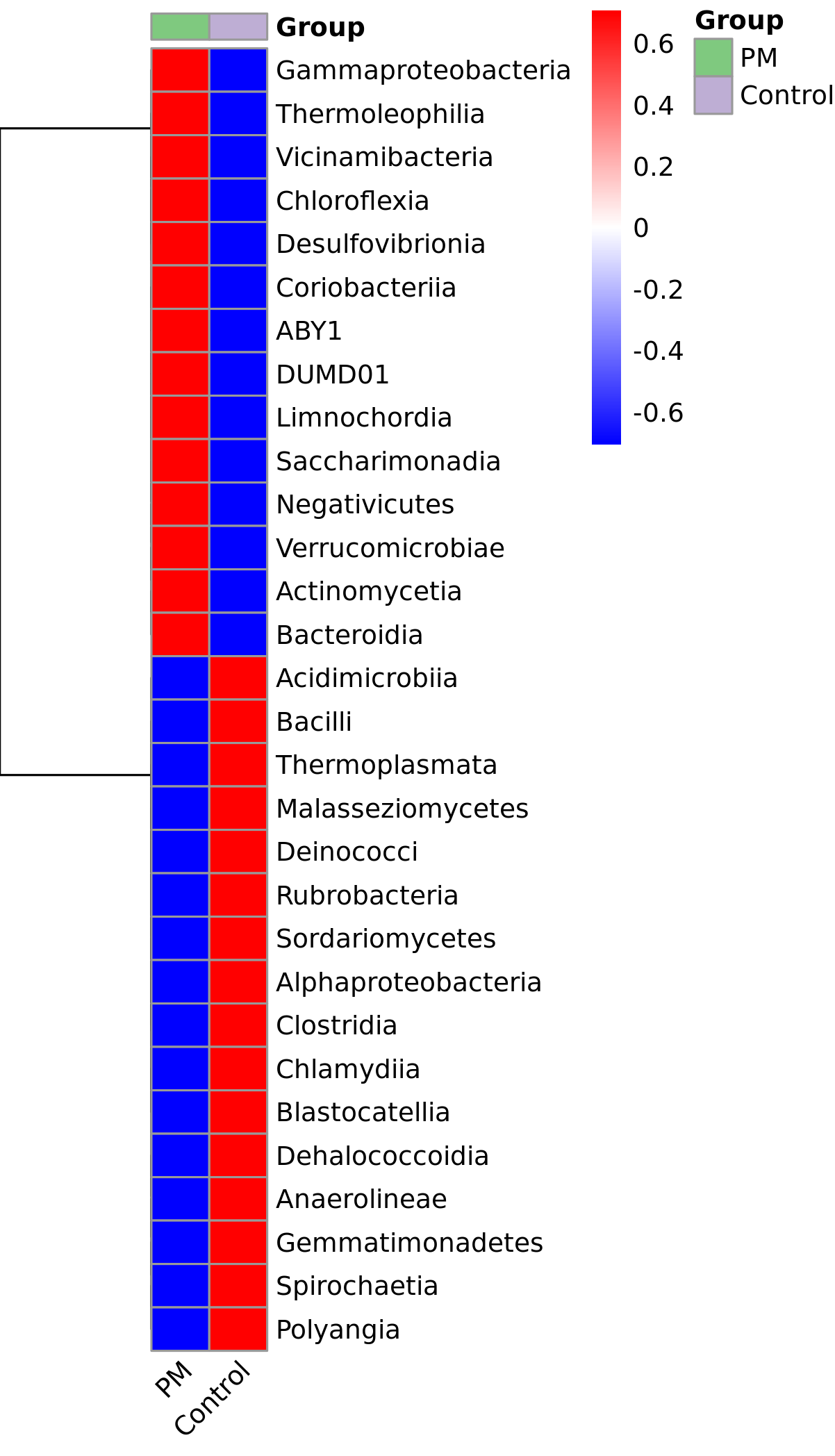

Supplement: Supplementary file 3 [file Data_Sheet_1.zip › 7.Average/1.Community_Structure/heatmap/C372089/Class_top30_nocluster.png]

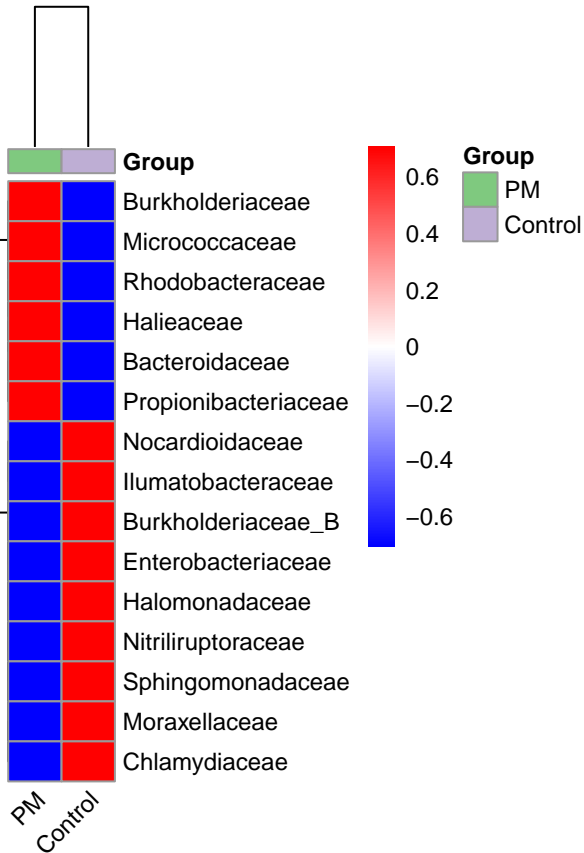

Supplement: Supplementary file 3 [file Data_Sheet_1.zip › 7.Average/1.Community_Structure/heatmap/C372089/Family_top15_cluster.pdf]

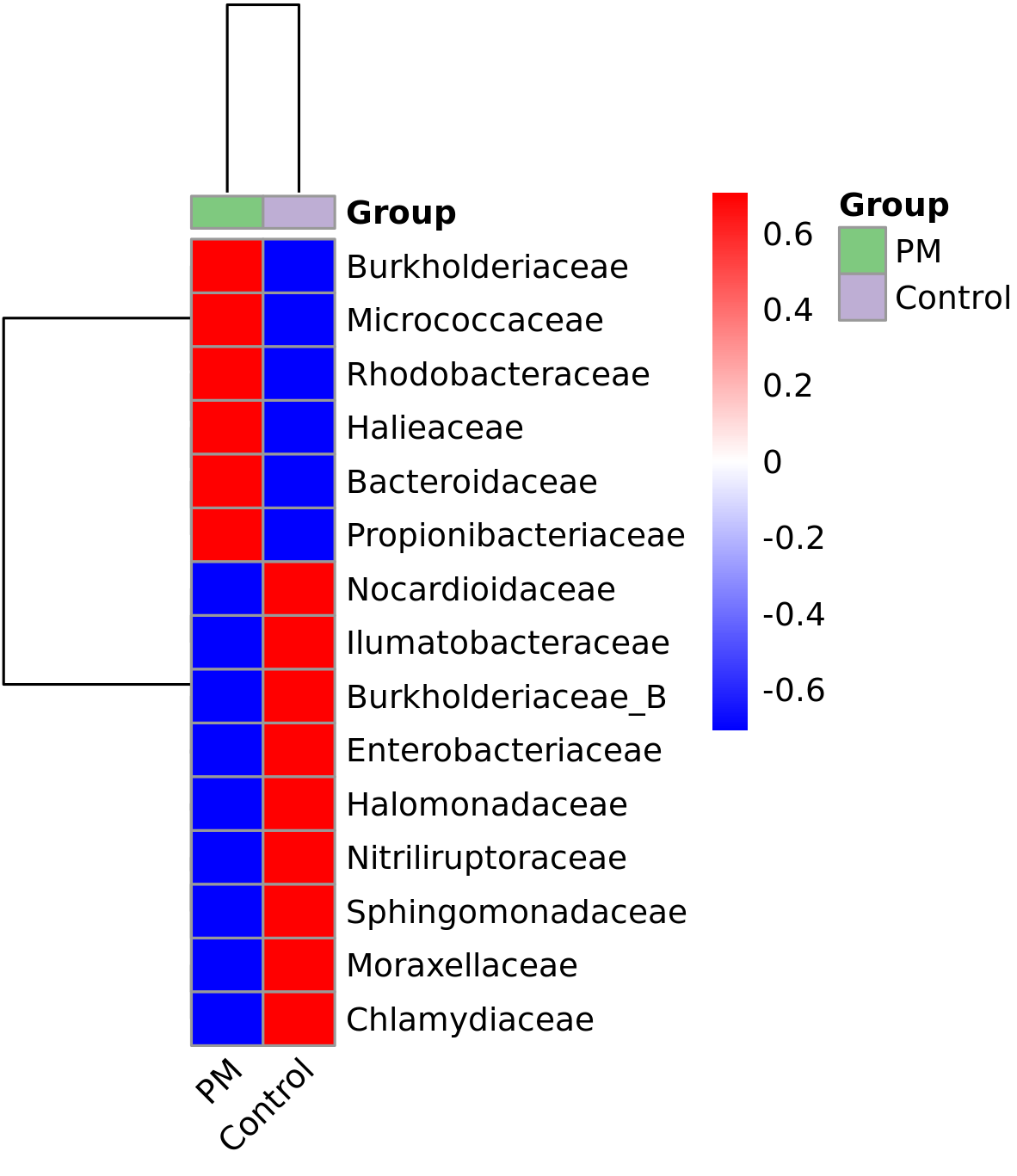

Supplement: Supplementary file 3 [file Data_Sheet_1.zip › 7.Average/1.Community_Structure/heatmap/C372089/Family_top15_cluster.png]

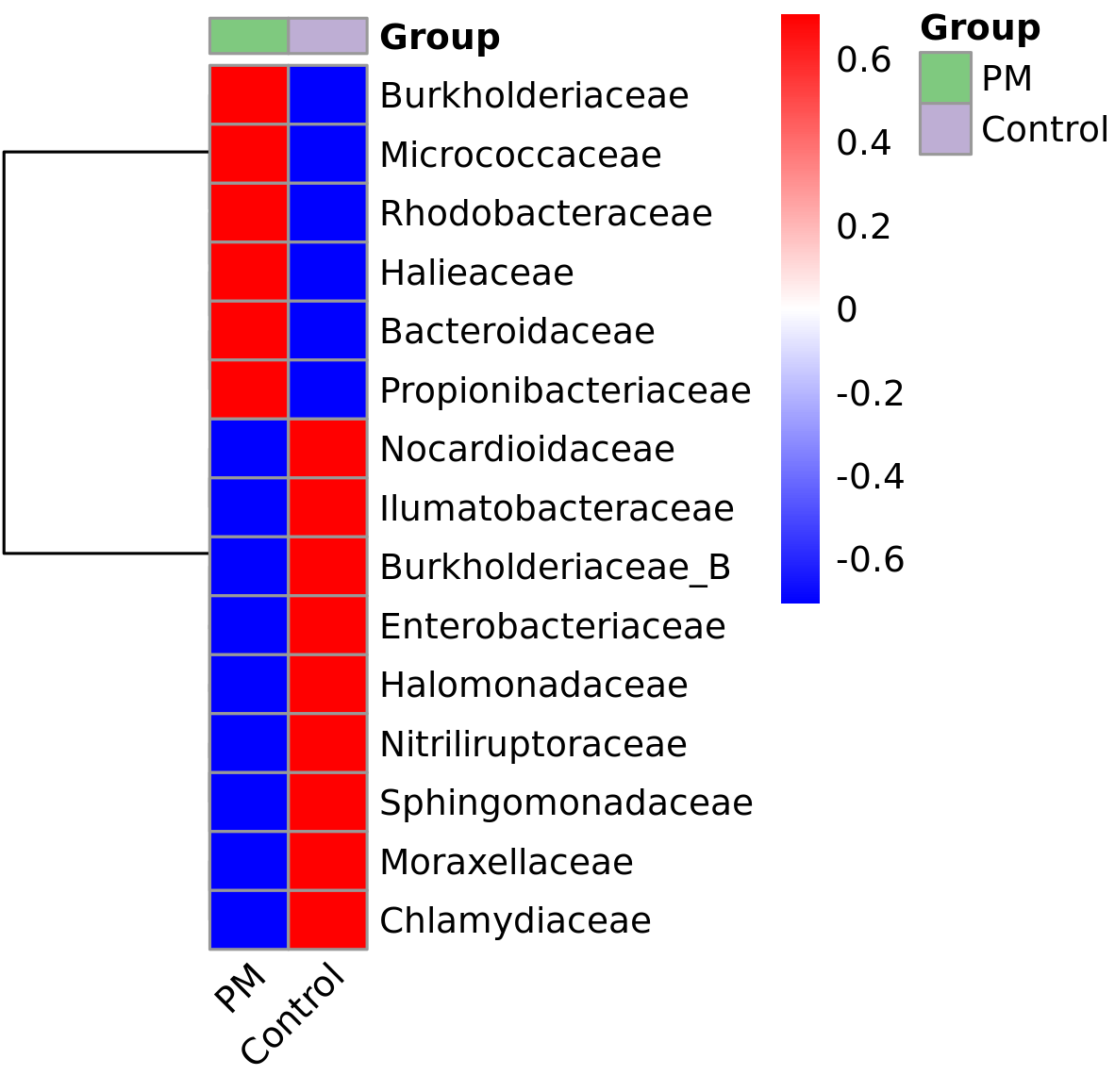

Supplement: Supplementary file 3 [file Data_Sheet_1.zip › 7.Average/1.Community_Structure/heatmap/C372089/Family_top15_nocluster.png]

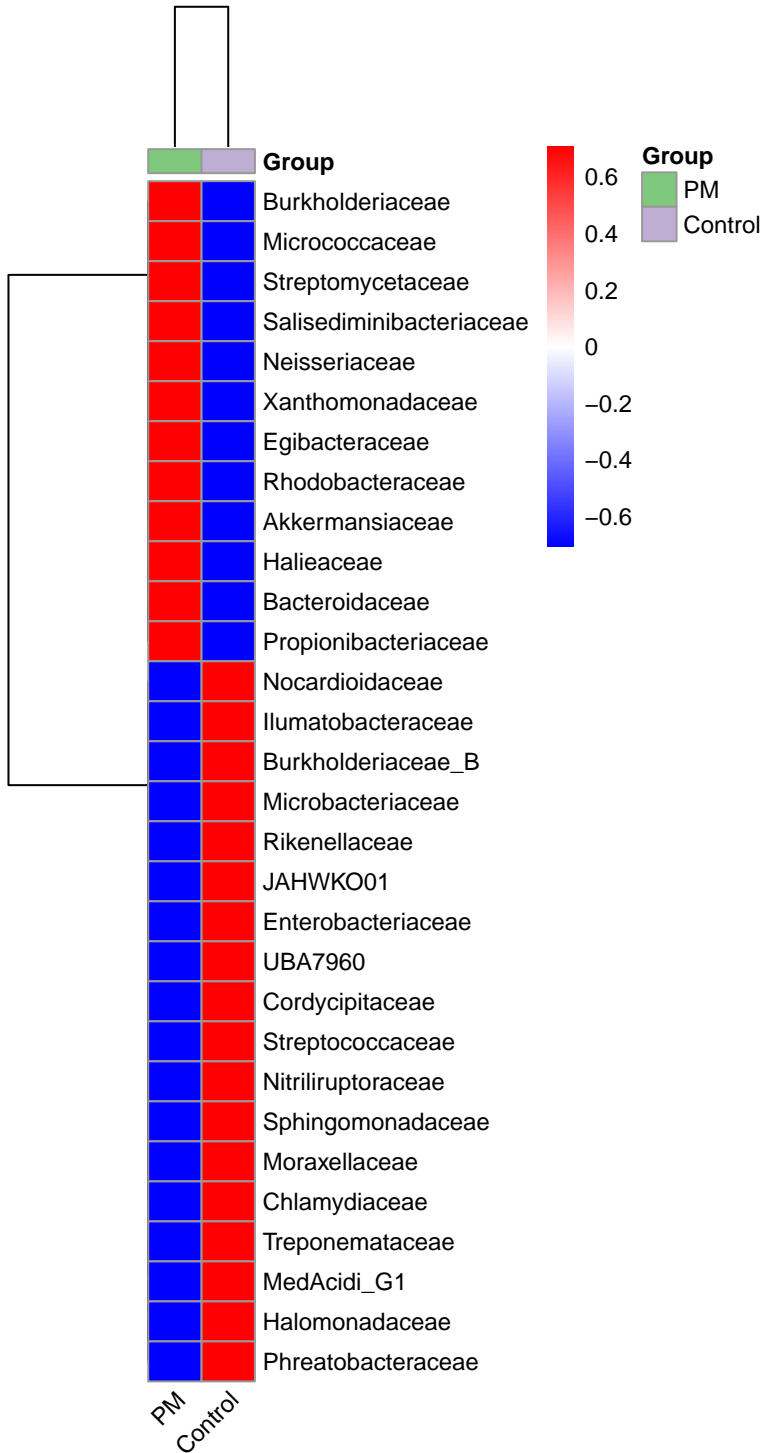

Supplement: Supplementary file 3 [file Data_Sheet_1.zip › 7.Average/1.Community_Structure/heatmap/C372089/Family_top30_cluster.pdf]

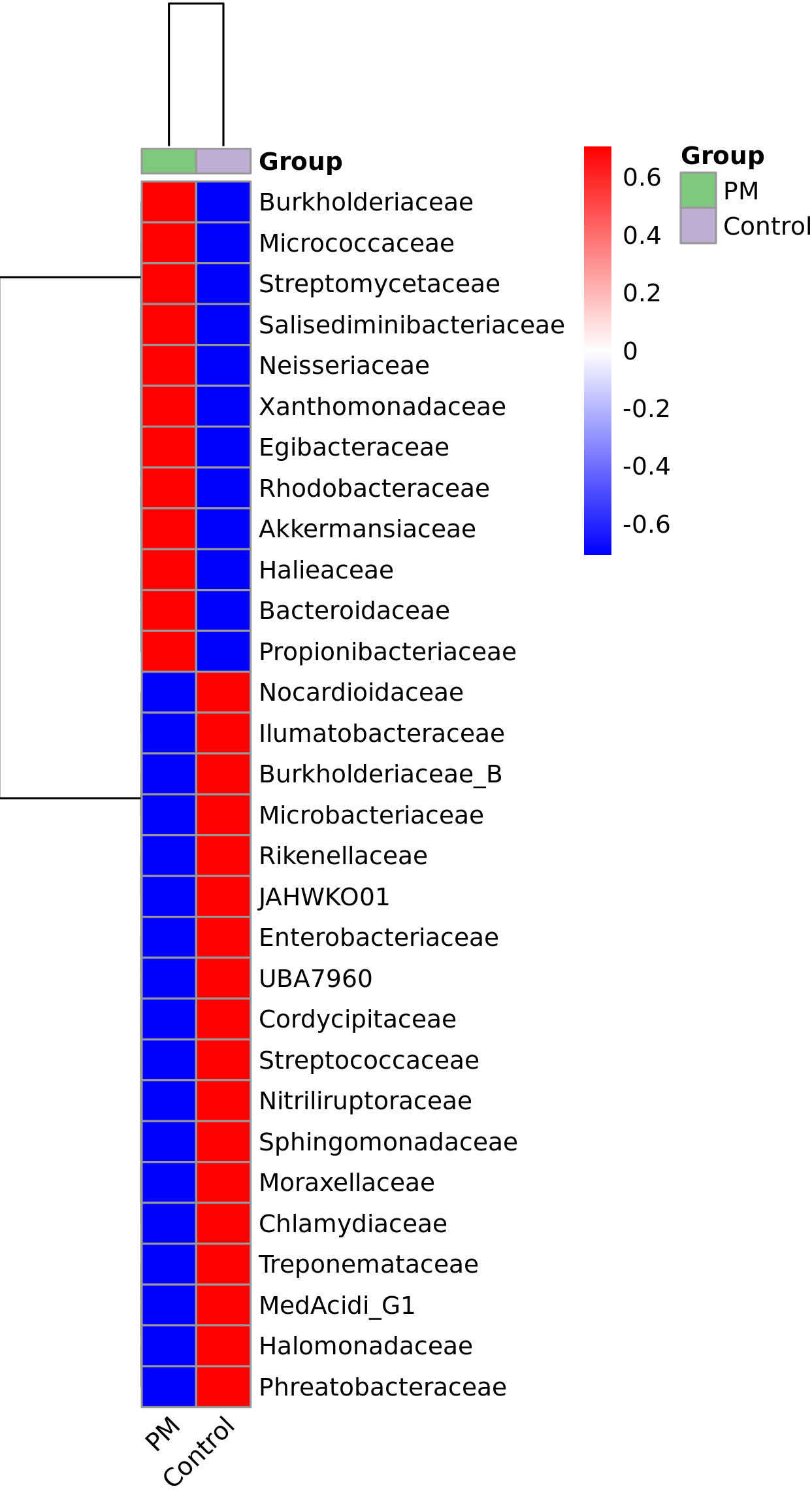

Supplement: Supplementary file 3 [file Data_Sheet_1.zip › 7.Average/1.Community_Structure/heatmap/C372089/Family_top30_cluster.png]

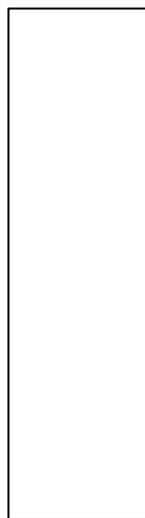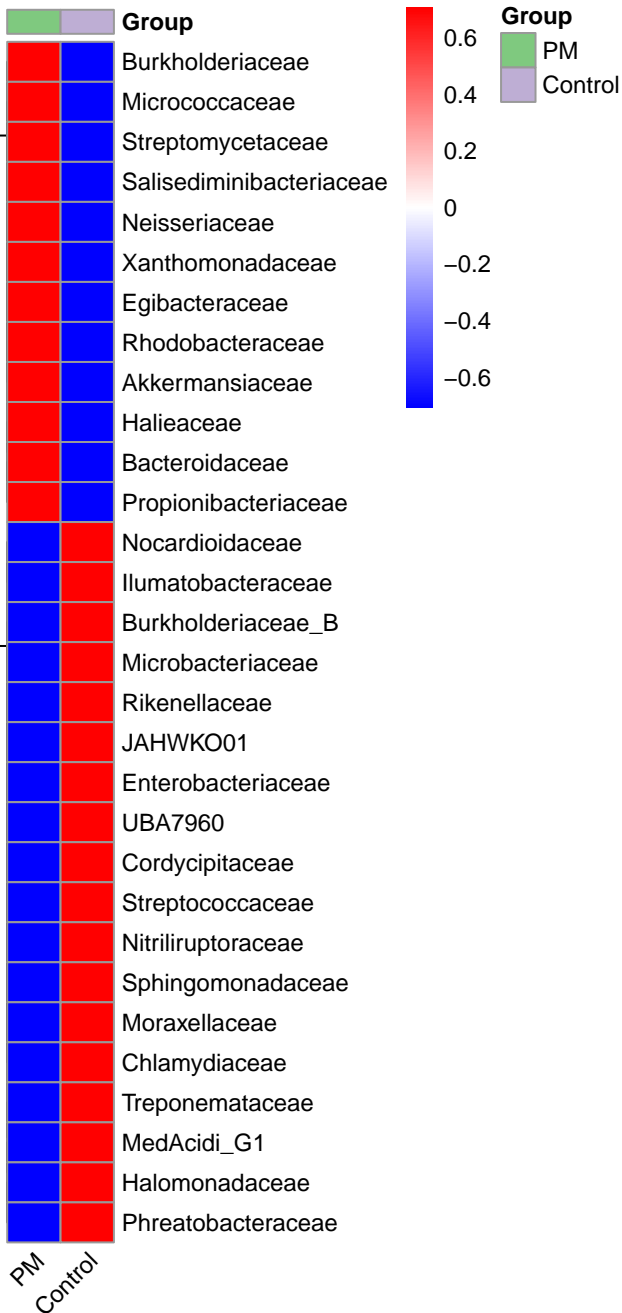

Supplement: Supplementary file 3 [file Data_Sheet_1.zip › 7.Average/1.Community_Structure/heatmap/C372089/Family_top30_nocluster.pdf]

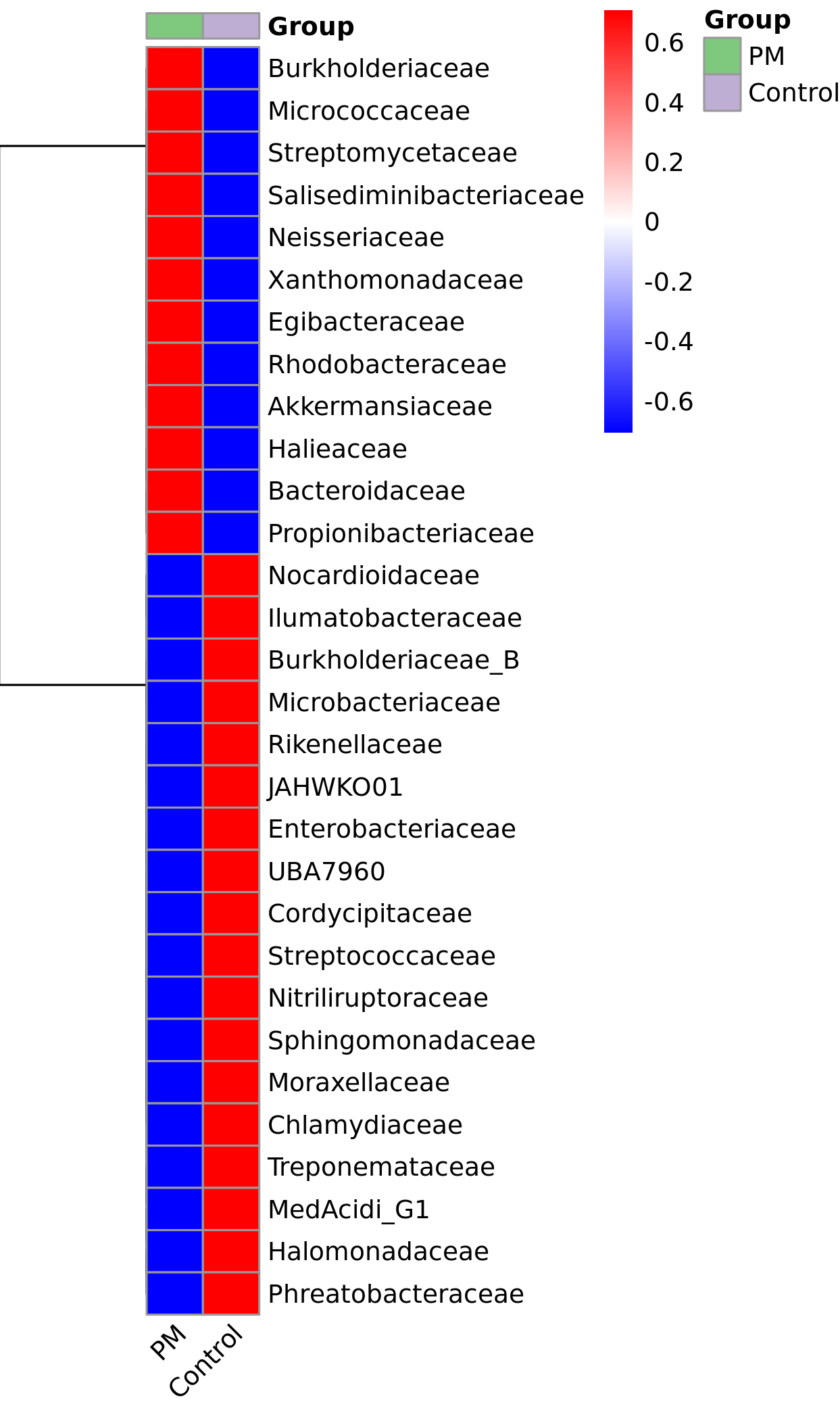

Supplement: Supplementary file 3 [file Data_Sheet_1.zip › 7.Average/1.Community_Structure/heatmap/C372089/Family_top30_nocluster.png]

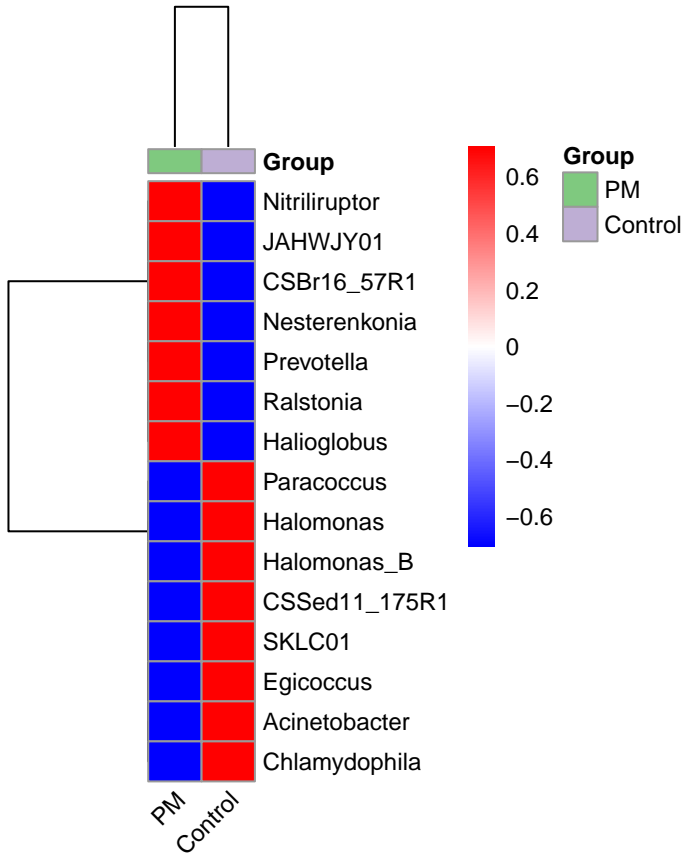

Supplement: Supplementary file 3 [file Data_Sheet_1.zip › 7.Average/1.Community_Structure/heatmap/C372089/Genus_top15_cluster.pdf]

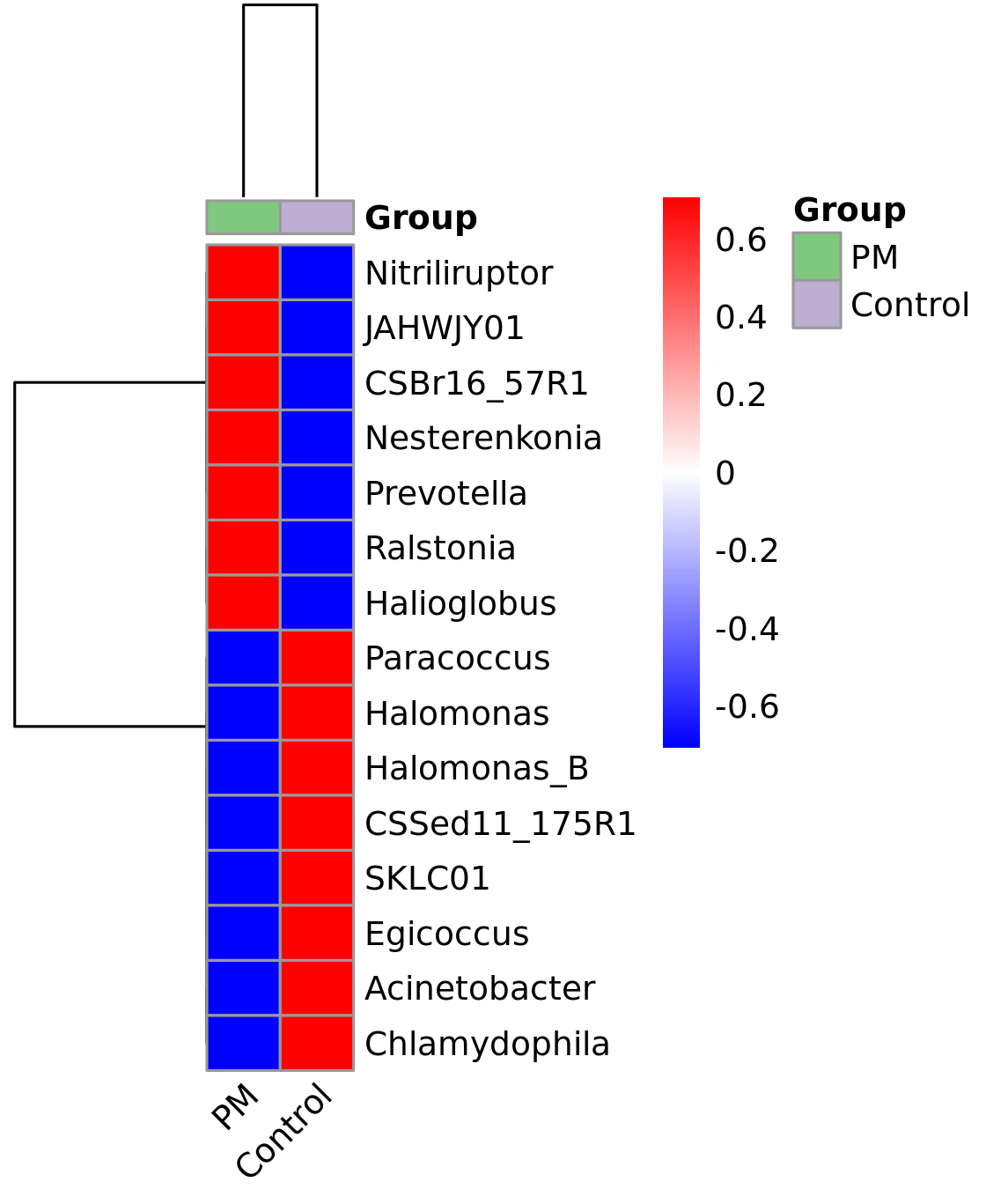

Supplement: Supplementary file 3 [file Data_Sheet_1.zip › 7.Average/1.Community_Structure/heatmap/C372089/Genus_top15_cluster.png]

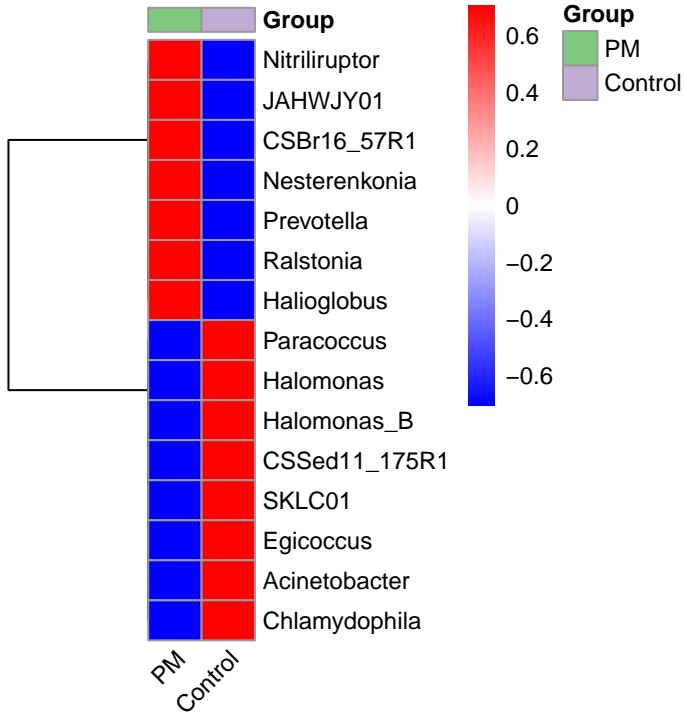

Supplement: Supplementary file 3 [file Data_Sheet_1.zip › 7.Average/1.Community_Structure/heatmap/C372089/Genus_top15_nocluster.pdf]

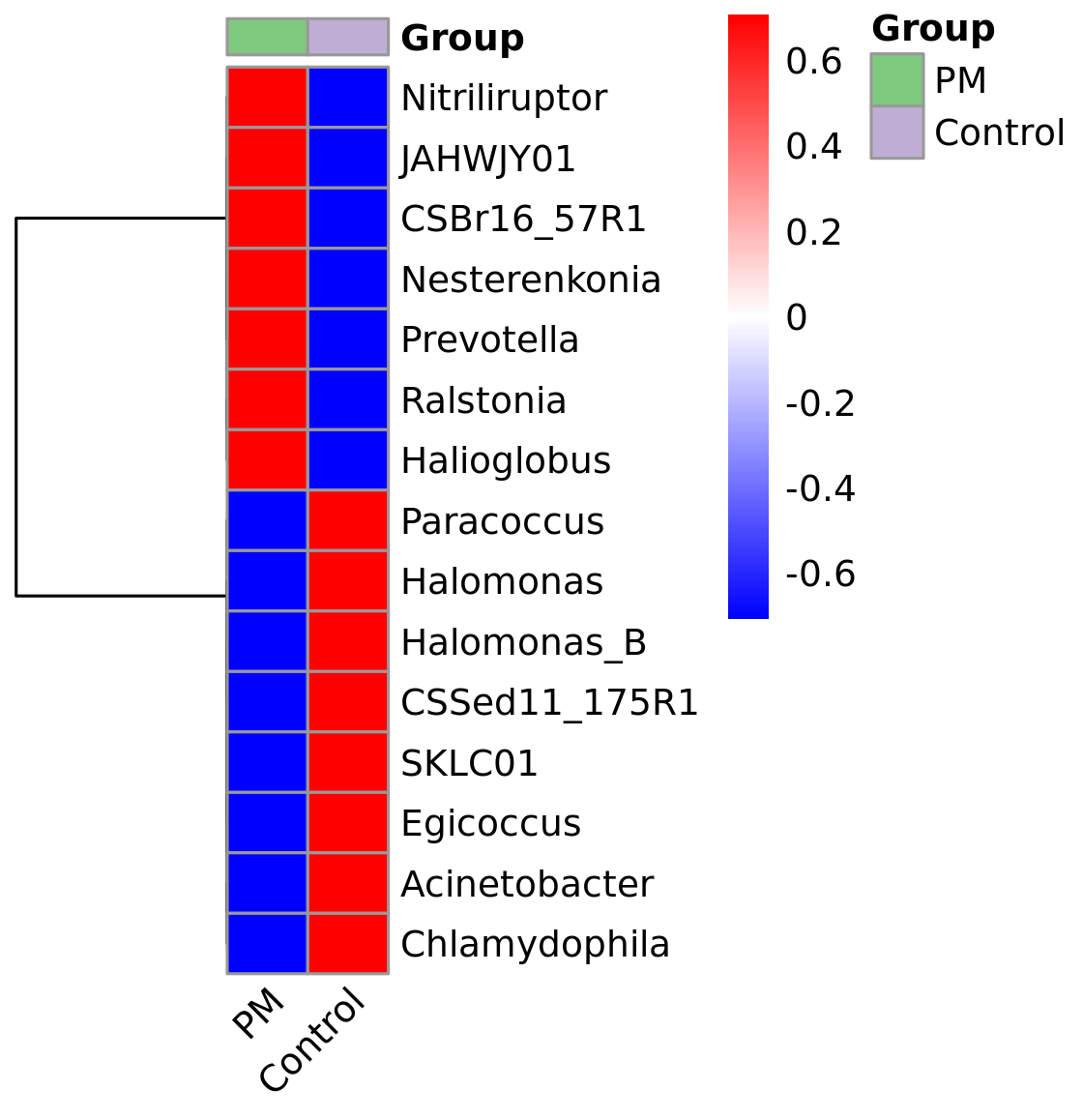

Supplement: Supplementary file 3 [file Data_Sheet_1.zip › 7.Average/1.Community_Structure/heatmap/C372089/Genus_top15_nocluster.png]

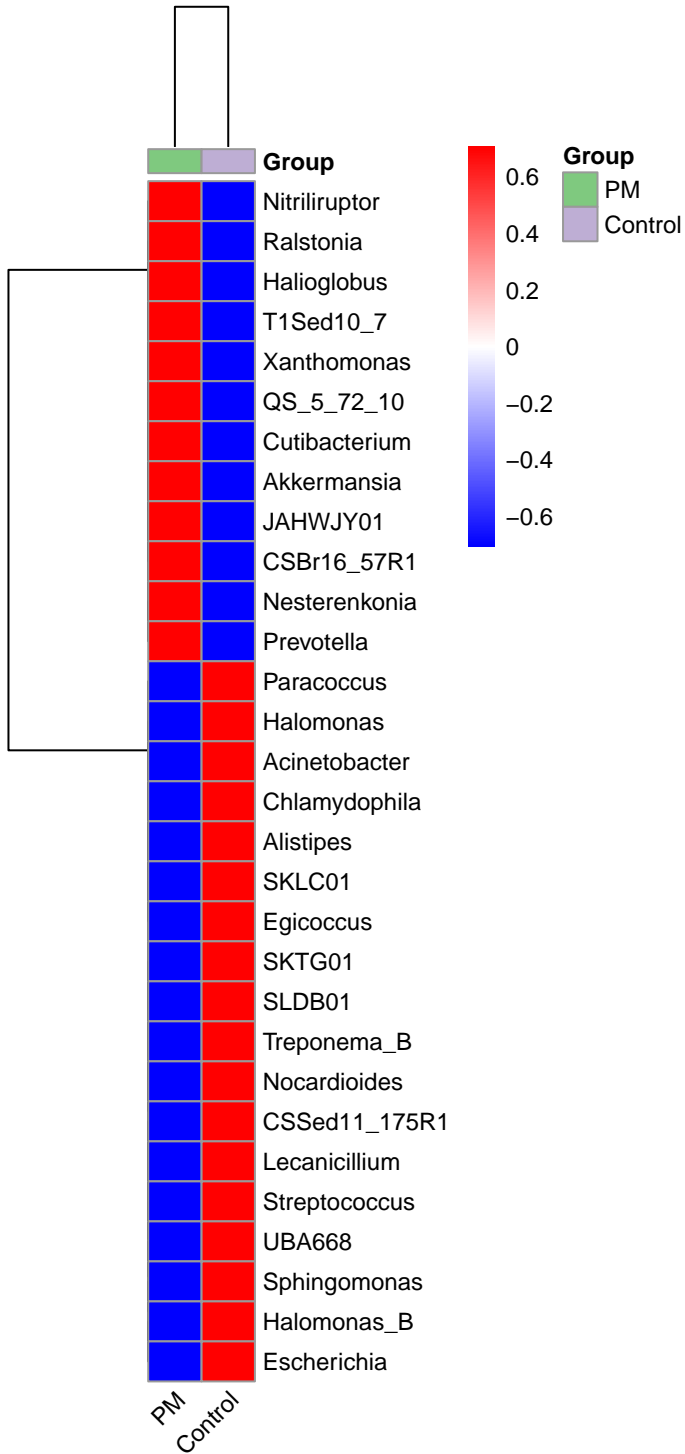

Supplement: Supplementary file 3 [file Data_Sheet_1.zip › 7.Average/1.Community_Structure/heatmap/C372089/Genus_top30_cluster.pdf]

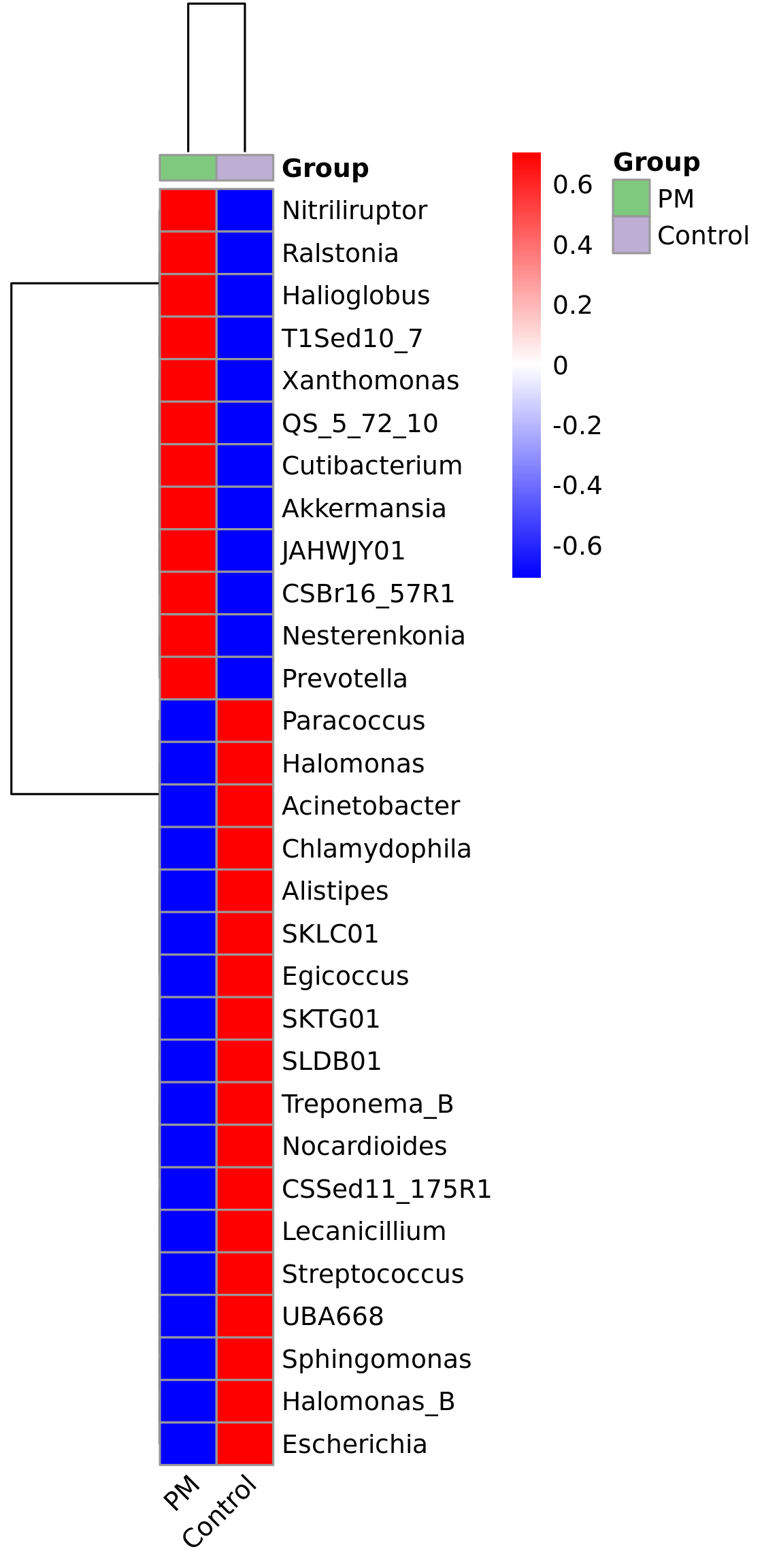

Supplement: Supplementary file 3 [file Data_Sheet_1.zip › 7.Average/1.Community_Structure/heatmap/C372089/Genus_top30_cluster.png]

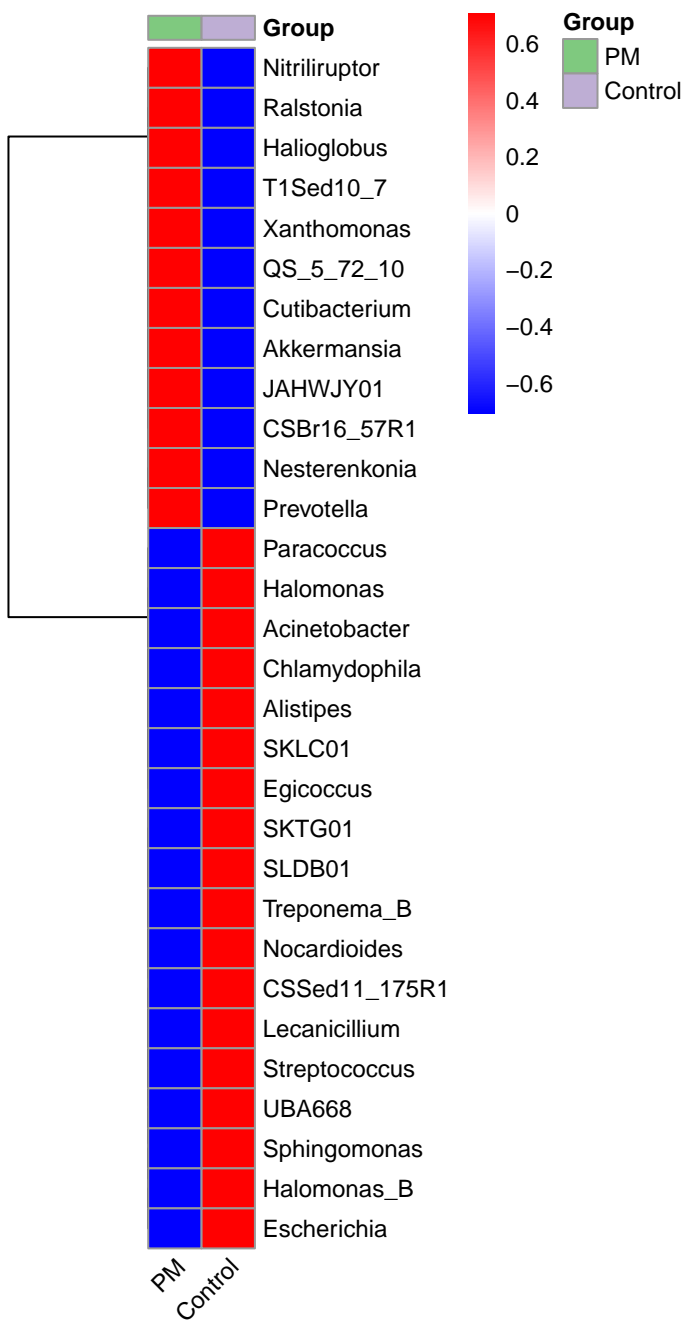

Supplement: Supplementary file 3 [file Data_Sheet_1.zip › 7.Average/1.Community_Structure/heatmap/C372089/Genus_top30_nocluster.pdf]

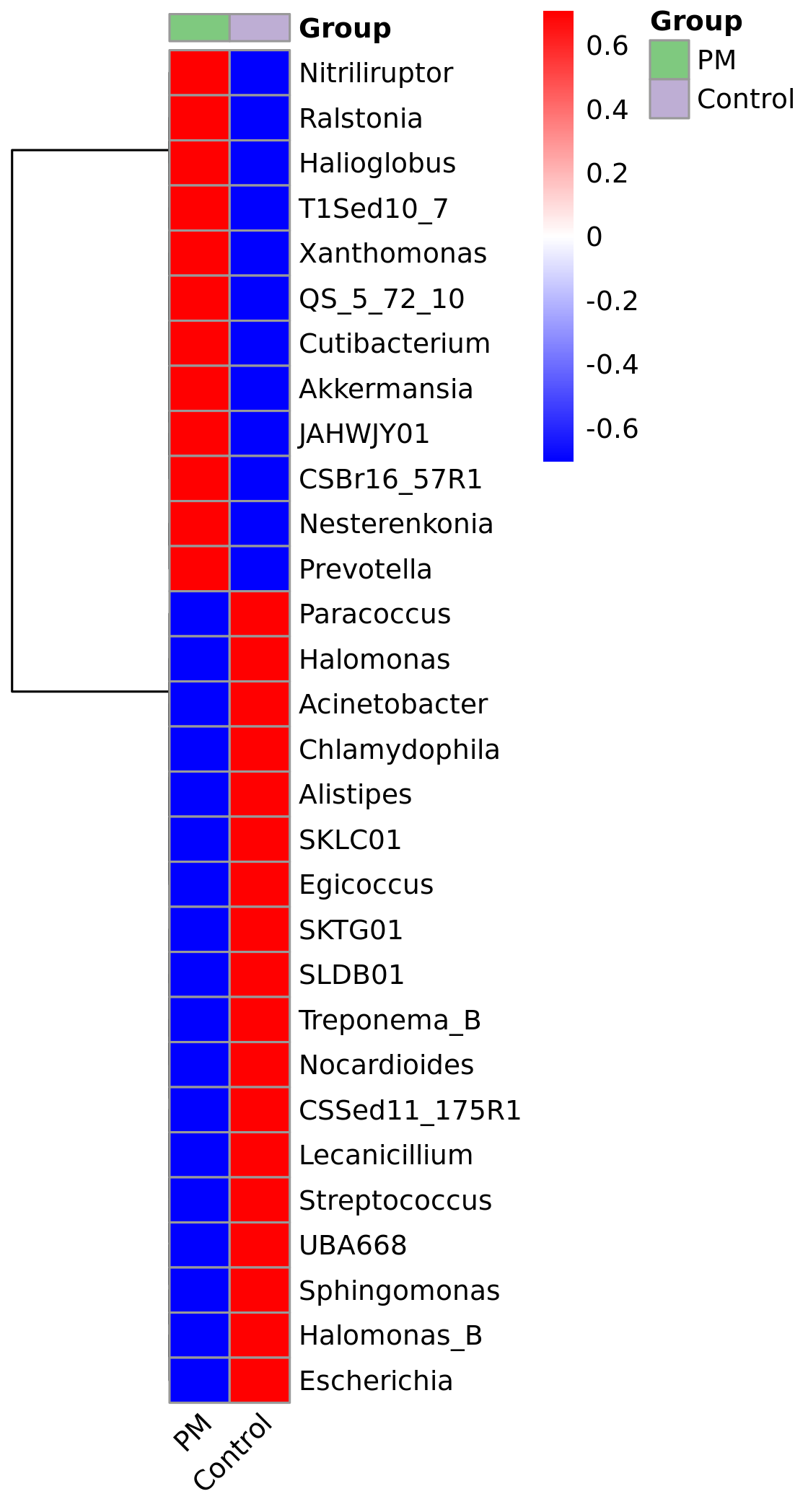

Supplement: Supplementary file 3 [file Data_Sheet_1.zip › 7.Average/1.Community_Structure/heatmap/C372089/Genus_top30_nocluster.png]

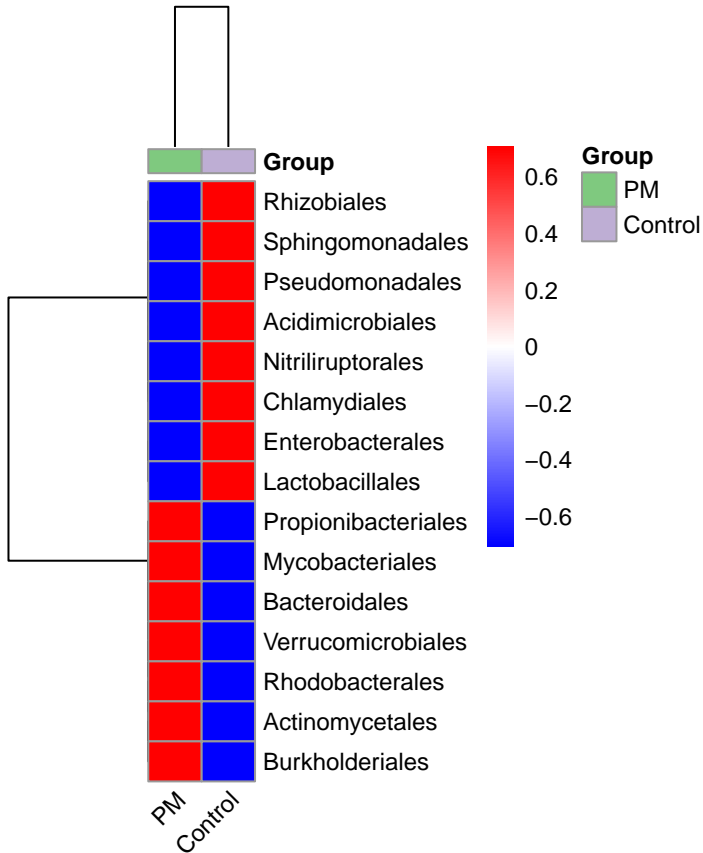

Supplement: Supplementary file 3 [file Data_Sheet_1.zip › 7.Average/1.Community_Structure/heatmap/C372089/Order_top15_cluster.pdf]

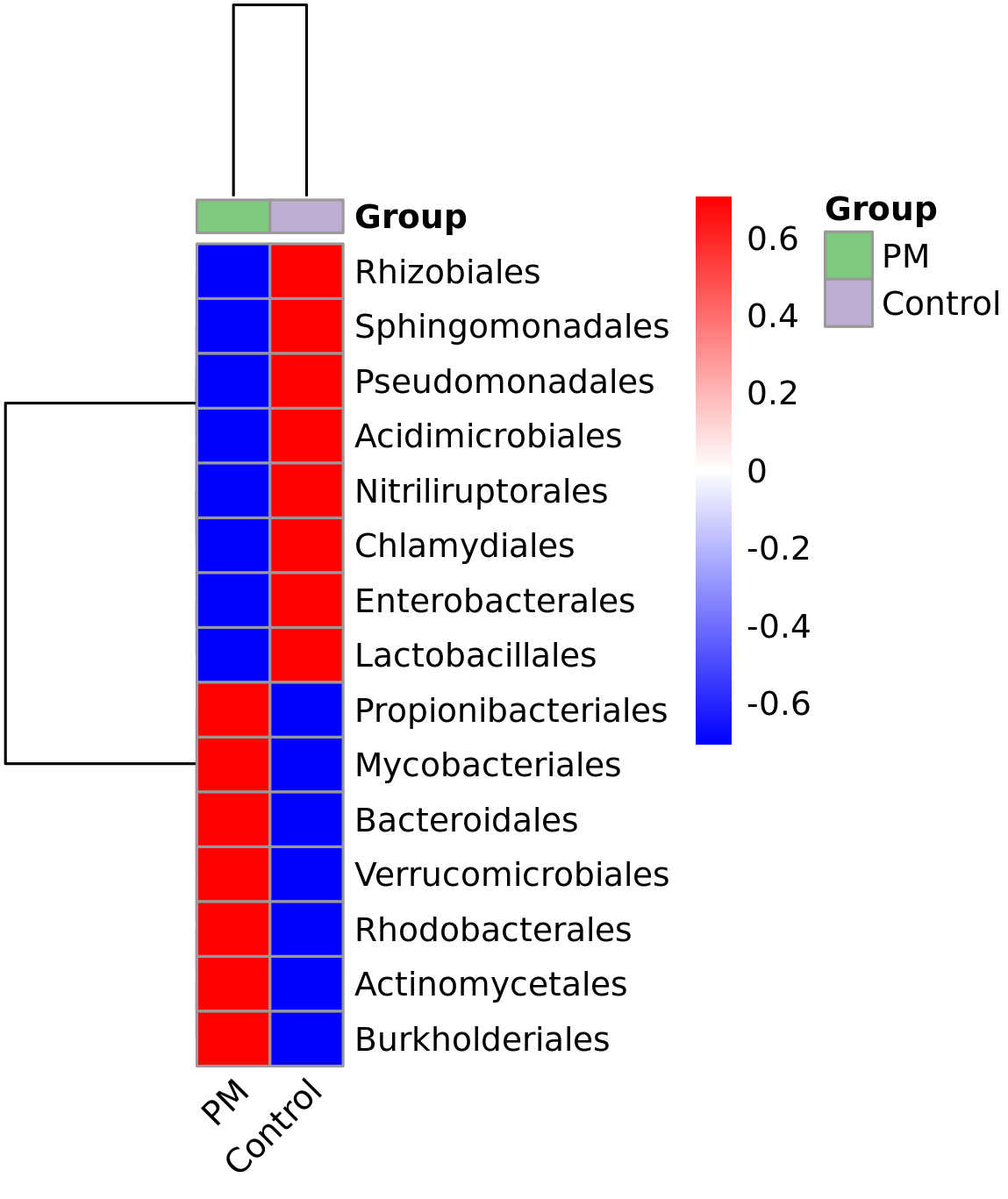

Supplement: Supplementary file 3 [file Data_Sheet_1.zip › 7.Average/1.Community_Structure/heatmap/C372089/Order_top15_cluster.png]

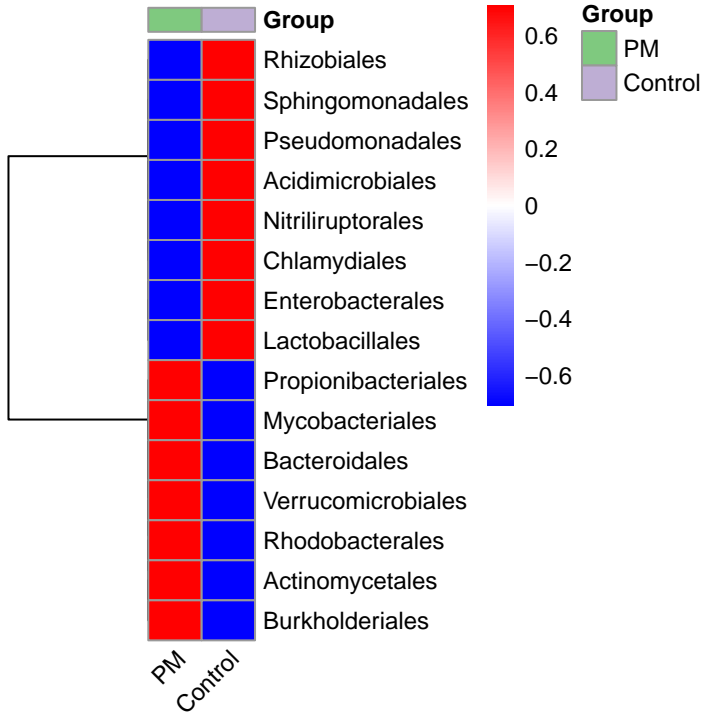

Supplement: Supplementary file 3 [file Data_Sheet_1.zip › 7.Average/1.Community_Structure/heatmap/C372089/Order_top15_nocluster.pdf]

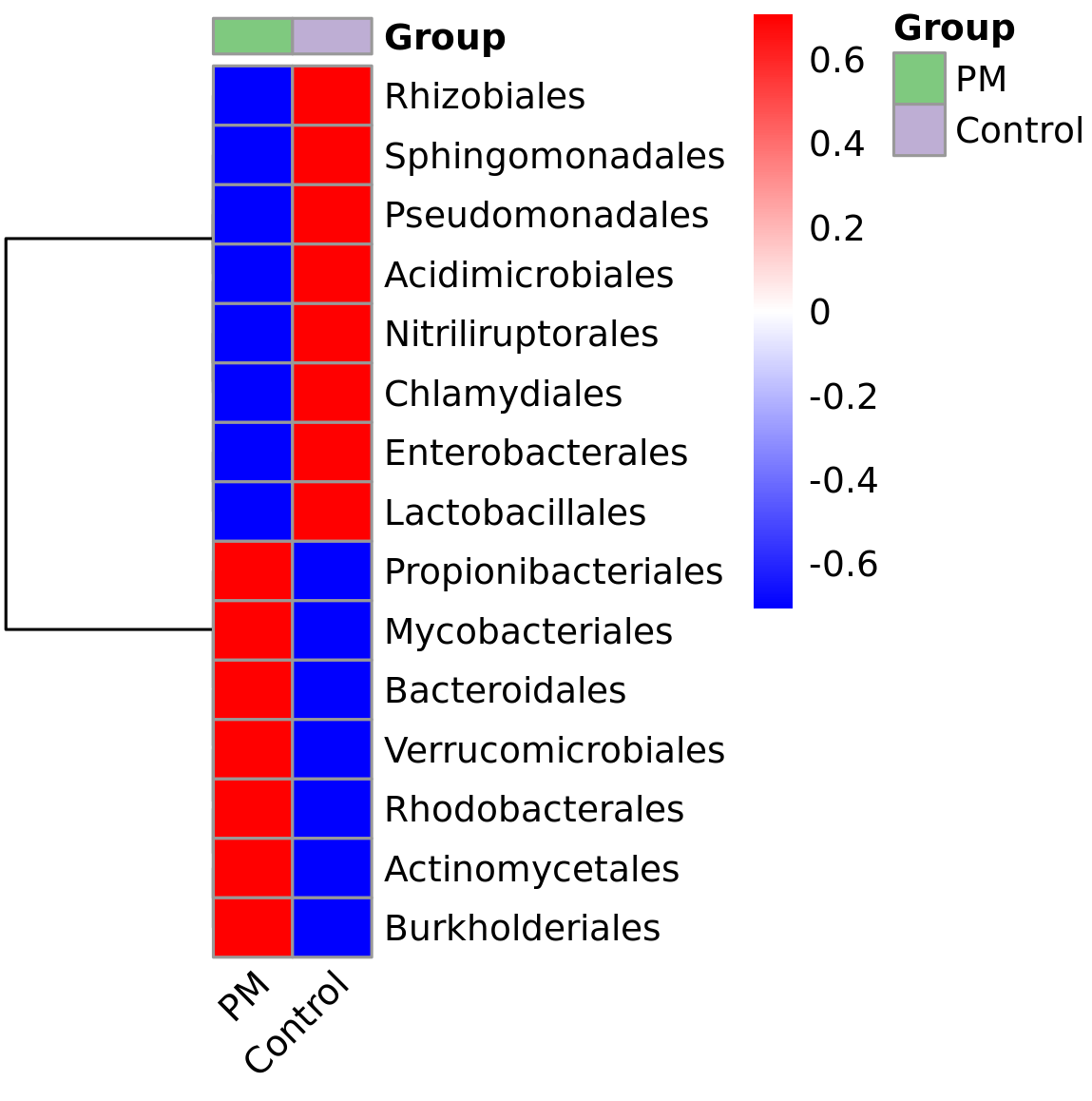

Supplement: Supplementary file 3 [file Data_Sheet_1.zip › 7.Average/1.Community_Structure/heatmap/C372089/Order_top15_nocluster.png]

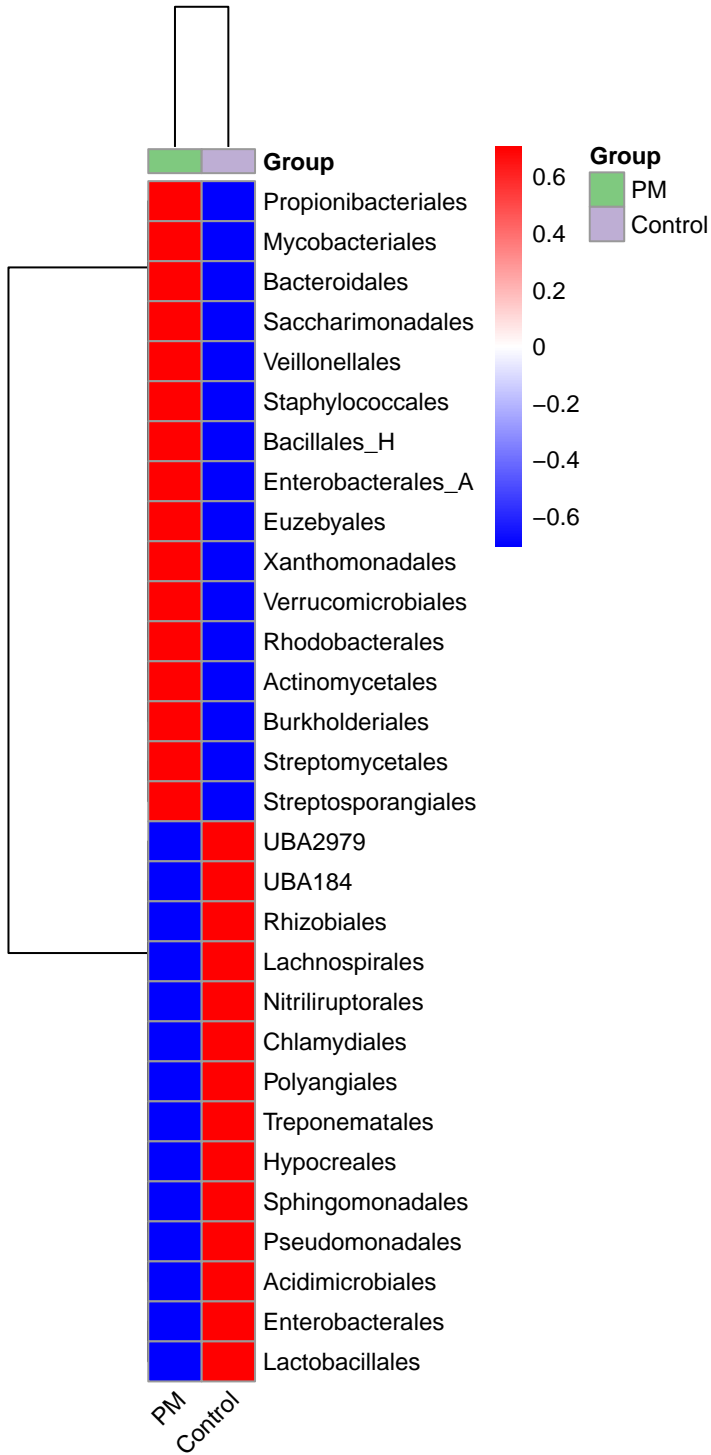

Supplement: Supplementary file 3 [file Data_Sheet_1.zip › 7.Average/1.Community_Structure/heatmap/C372089/Order_top30_cluster.pdf]

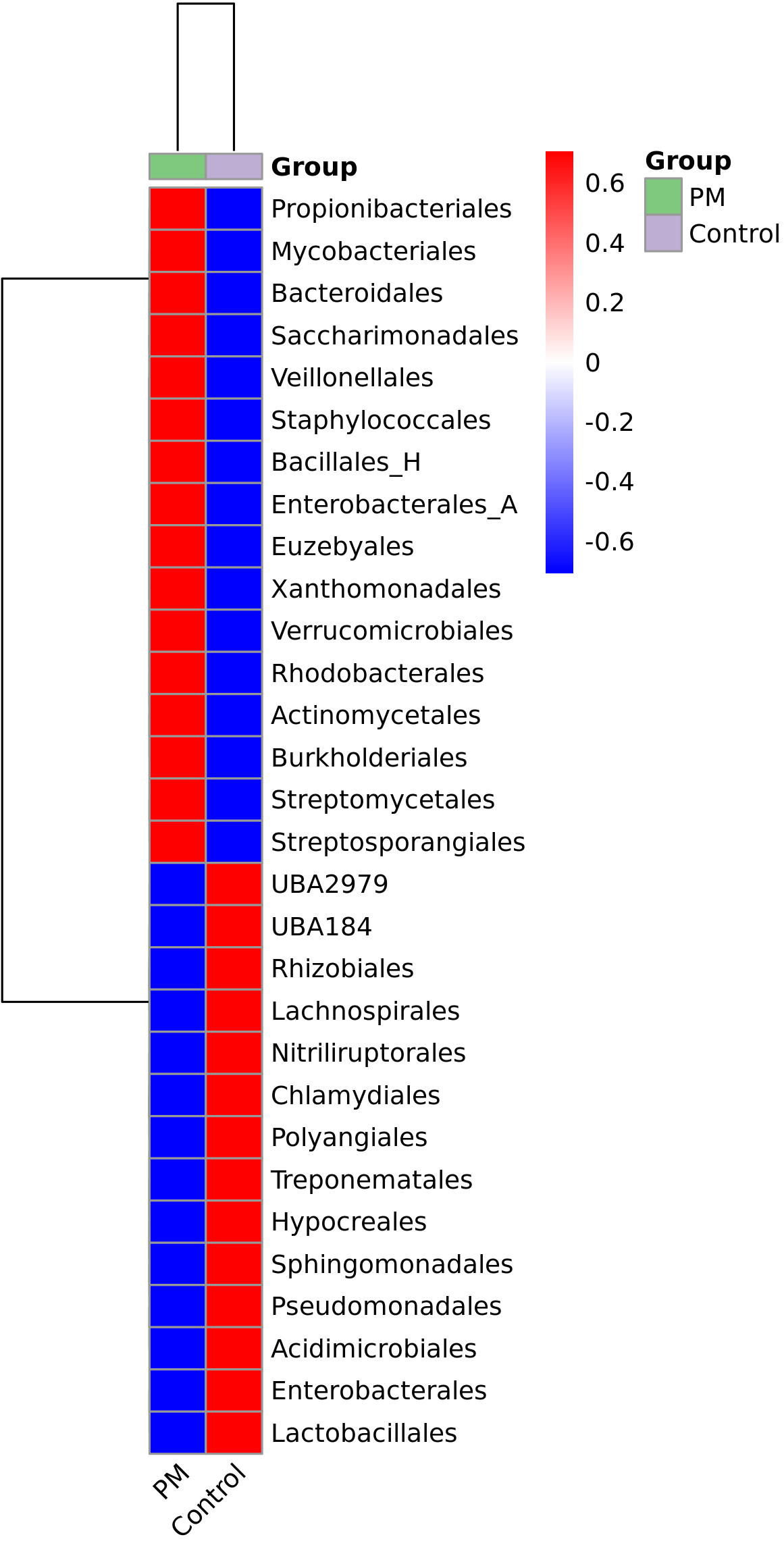

Supplement: Supplementary file 3 [file Data_Sheet_1.zip › 7.Average/1.Community_Structure/heatmap/C372089/Order_top30_cluster.png]

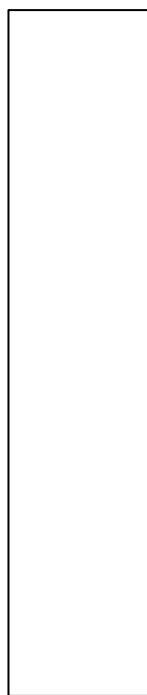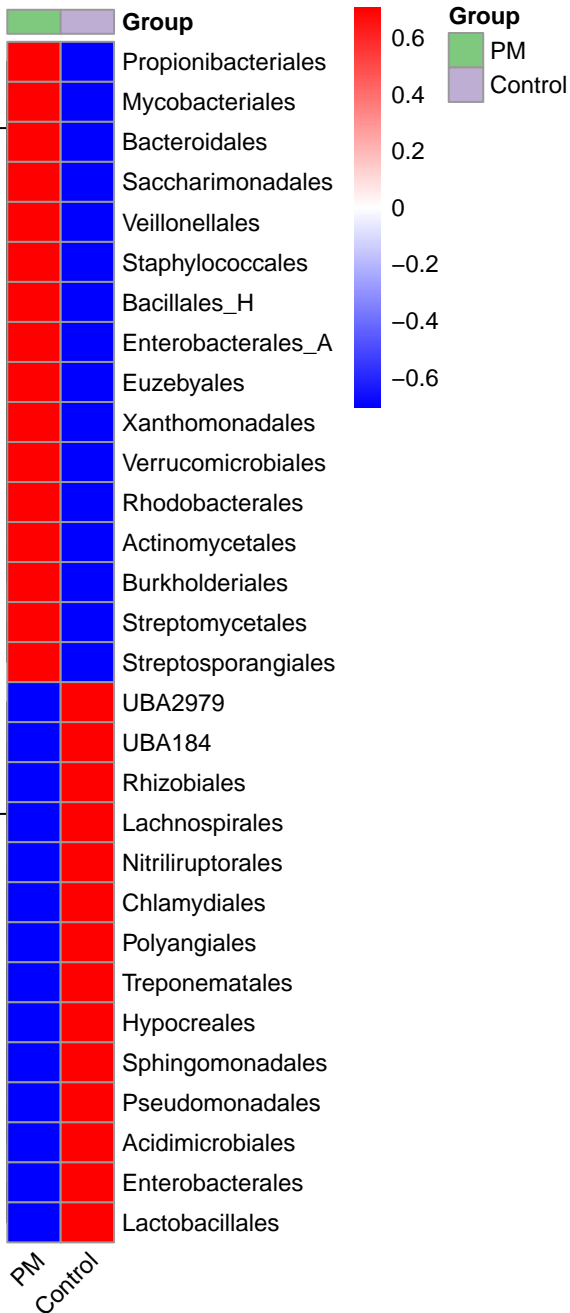

Supplement: Supplementary file 3 [file Data_Sheet_1.zip › 7.Average/1.Community_Structure/heatmap/C372089/Order_top30_nocluster.pdf]

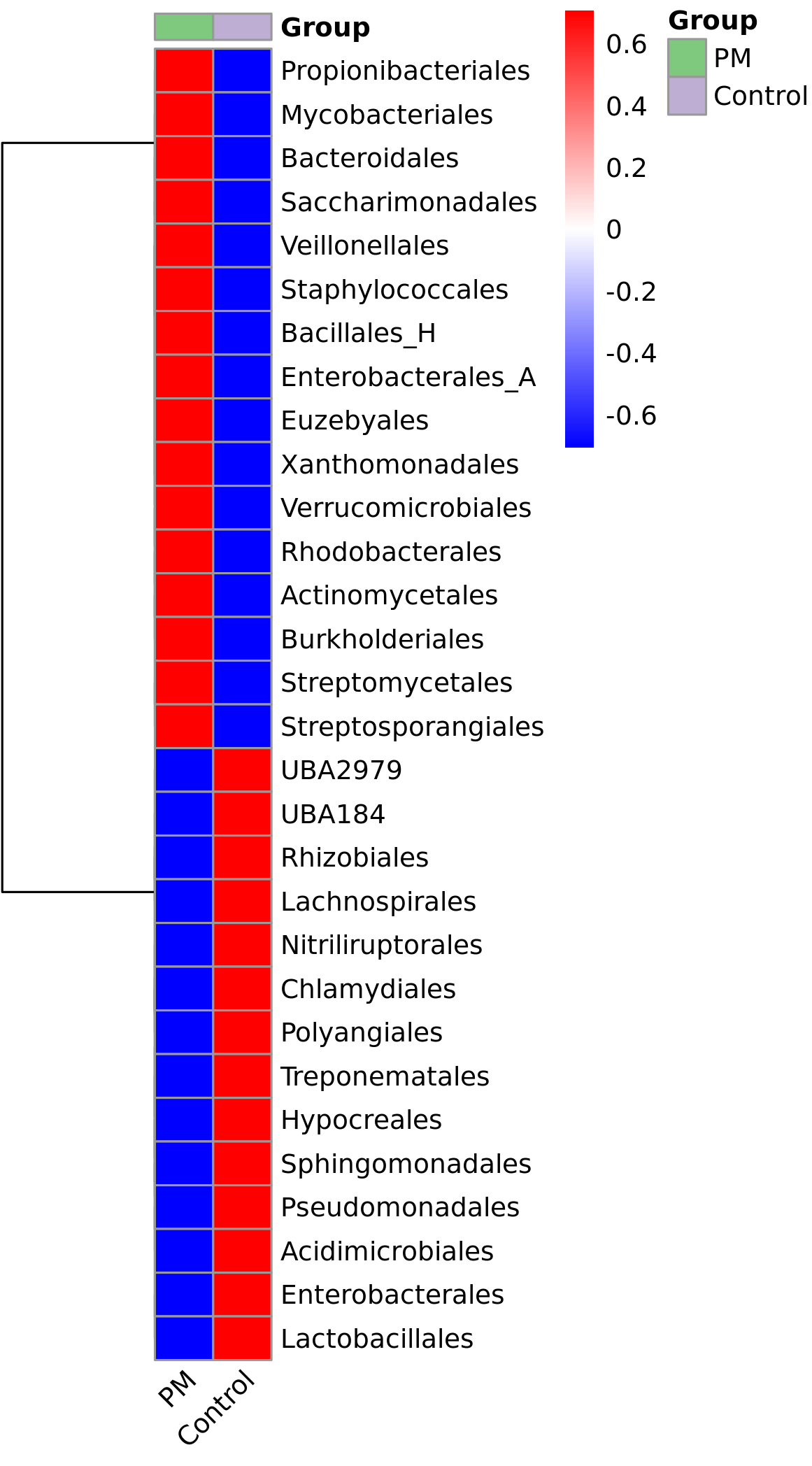

Supplement: Supplementary file 3 [file Data_Sheet_1.zip › 7.Average/1.Community_Structure/heatmap/C372089/Order_top30_nocluster.png]

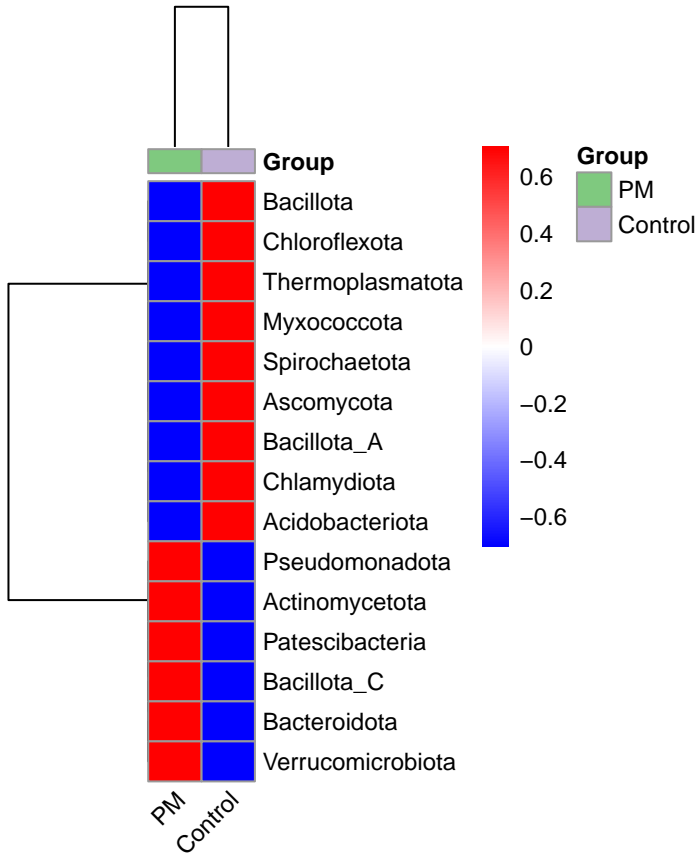

Supplement: Supplementary file 3 [file Data_Sheet_1.zip › 7.Average/1.Community_Structure/heatmap/C372089/Phylum_top15_cluster.pdf]

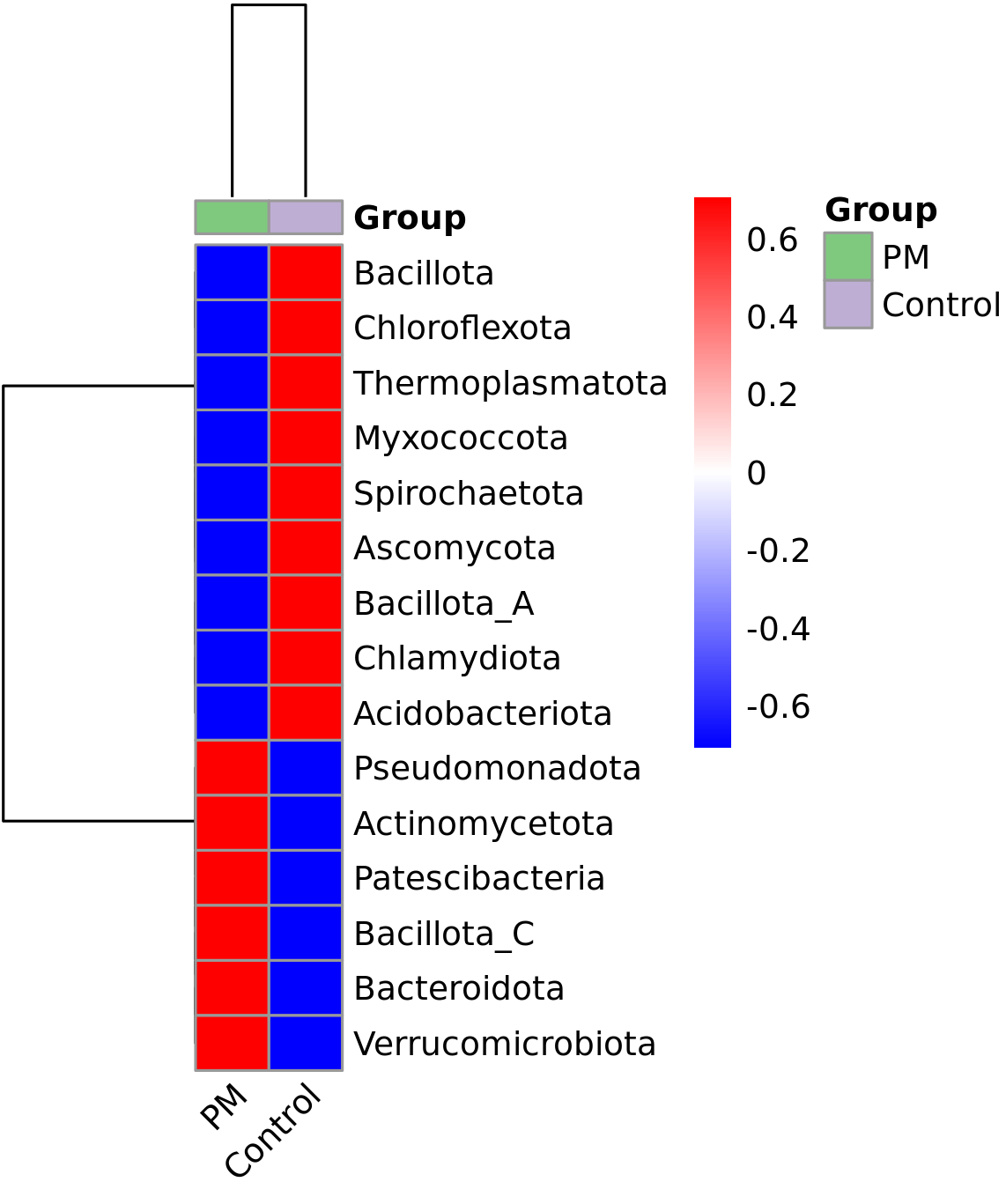

Supplement: Supplementary file 3 [file Data_Sheet_1.zip › 7.Average/1.Community_Structure/heatmap/C372089/Phylum_top15_cluster.png]

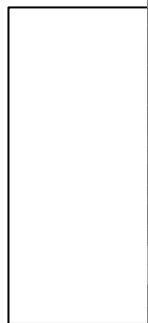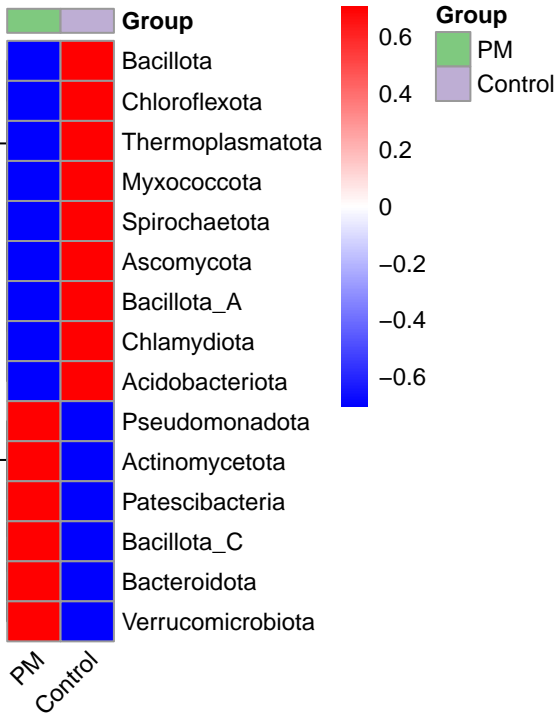

Supplement: Supplementary file 3 [file Data_Sheet_1.zip › 7.Average/1.Community_Structure/heatmap/C372089/Phylum_top15_nocluster.pdf]

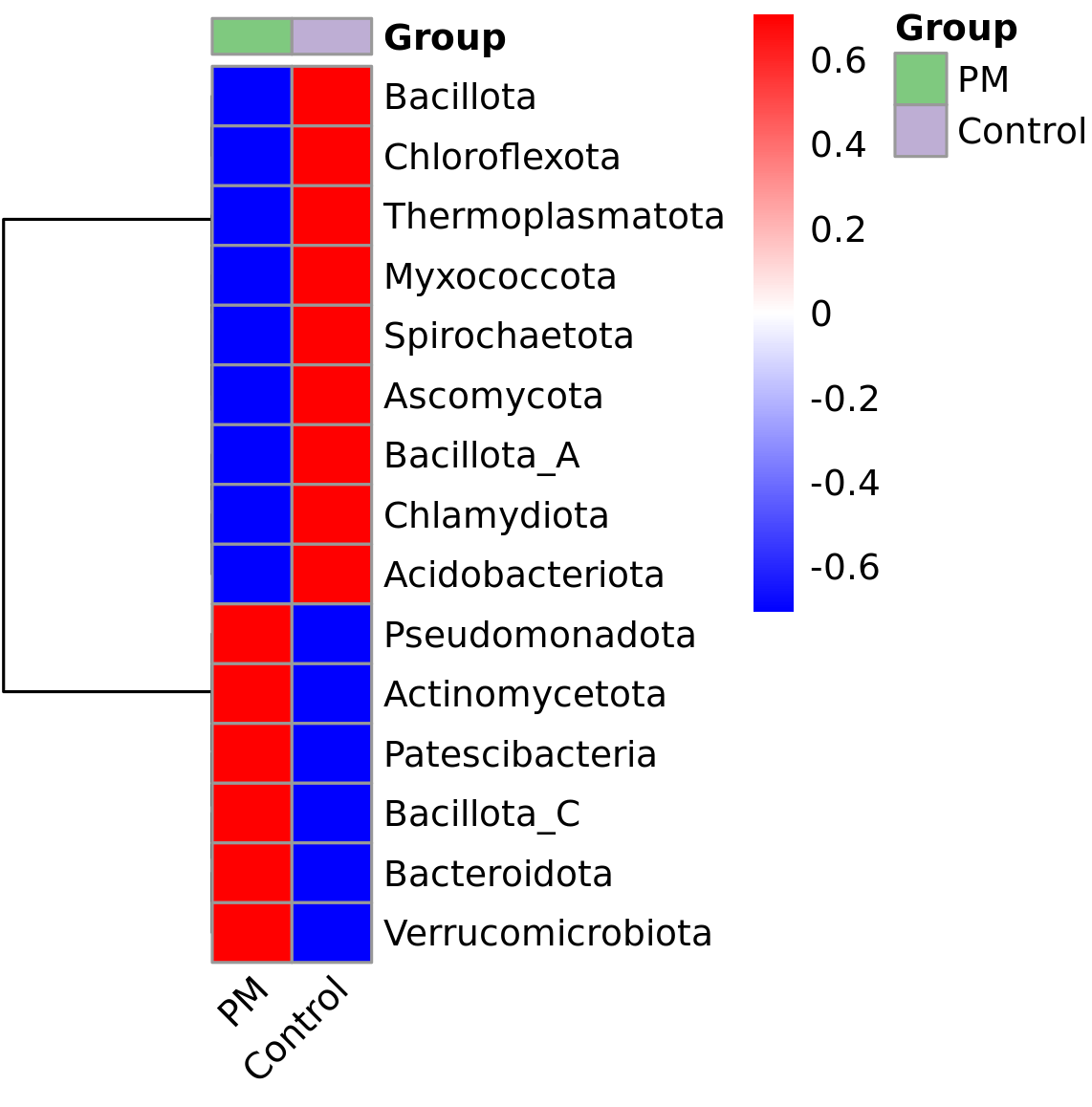

Supplement: Supplementary file 3 [file Data_Sheet_1.zip › 7.Average/1.Community_Structure/heatmap/C372089/Phylum_top15_nocluster.png]

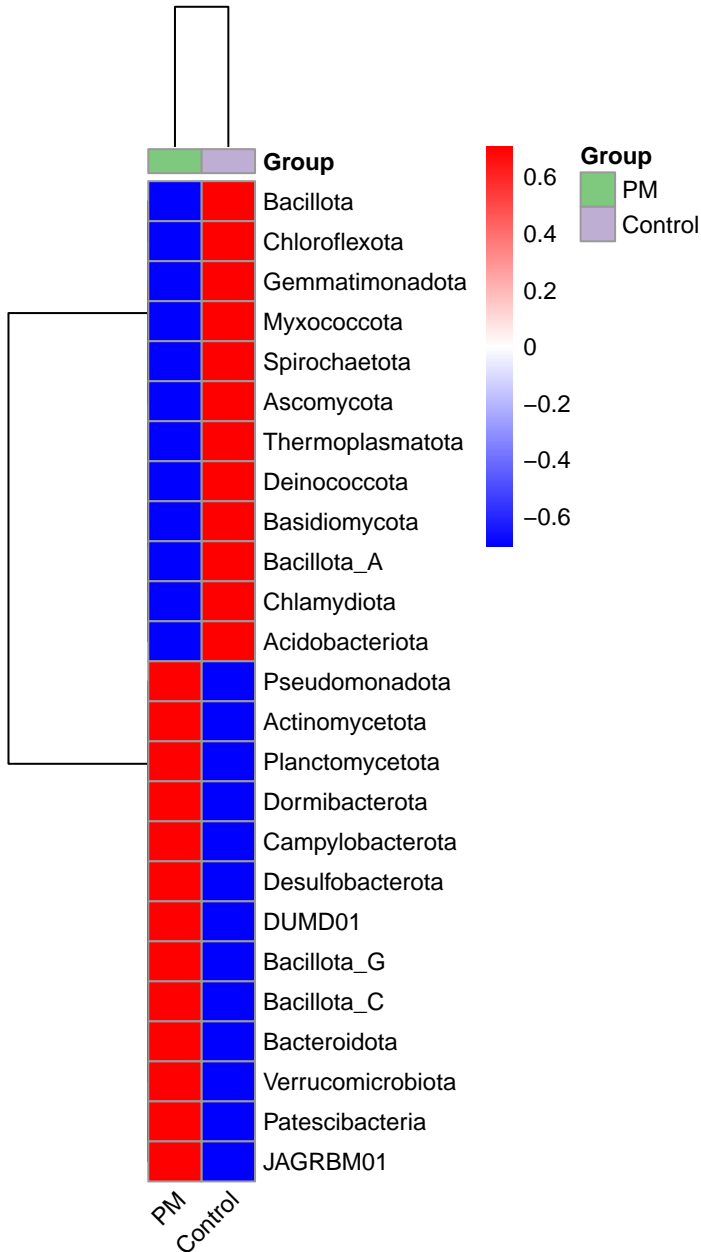

Supplement: Supplementary file 3 [file Data_Sheet_1.zip › 7.Average/1.Community_Structure/heatmap/C372089/Phylum_top30_cluster.pdf]

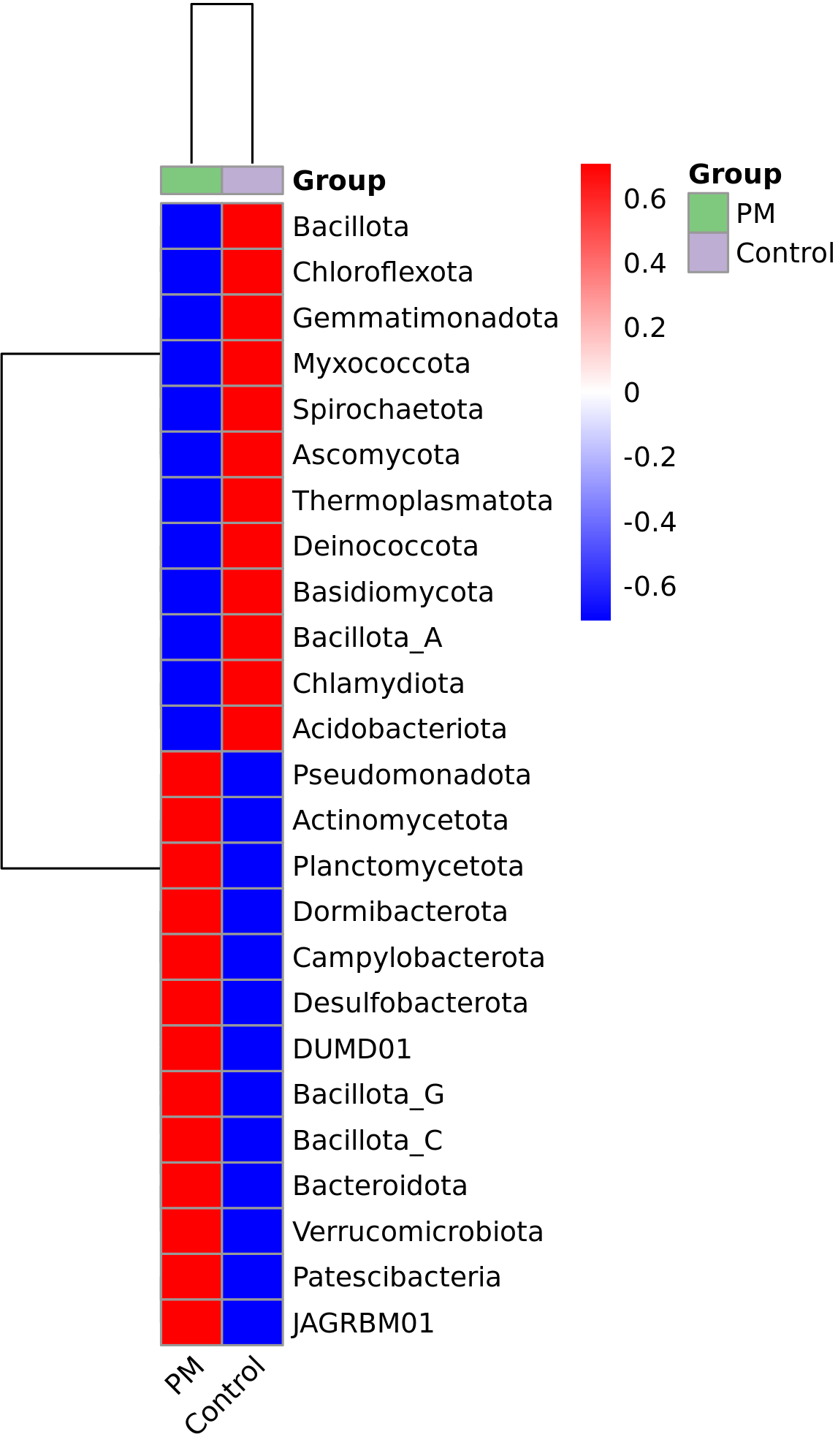

Supplement: Supplementary file 3 [file Data_Sheet_1.zip › 7.Average/1.Community_Structure/heatmap/C372089/Phylum_top30_cluster.png]
